# Reaction discovery using acetylene gas as the chemical feedstock accelerated by the “stop-flow” micro-tubing reactor system†
†Electronic supplementary information (ESI) available. See DOI: 10.1039/c7sc00408g


**DOI:** 10.1039/c7sc00408g

**Published:** 2017-02-27

**Authors:** Fei Xue, Hongping Deng, Chengwen Xue, Dara Khairunnisa Binte Mohamed, Karen Yuanting Tang, Jie Wu

**Affiliations:** a Department of Chemistry , National University of Singapore , 3 Science Drive 3 , Republic of Singapore 117543 . Email: chmjie@nus.edu.sg

## Abstract

Acetylene gas has been applied as a feedstock under transition-metal catalysis and photo-redox conditions to produce important chemicals including terminal alkynes, fulvenes, and fluorinated styrene compounds. The reaction discovery process was accelerated through the use of “stop-flow” micro-tubing reactors. This reactor prototype was developed by joining elements from both continuous micro-flow and conventional batch reactors, which was convenient and effective for gas/liquid reaction screening. Notably, the developed transformations were either inefficient or unsuccessful in conventional batch reactors. Its success relies on the unique advantages provided by this “stop-flow” micro-tubing reactor system.

## Abstract 2

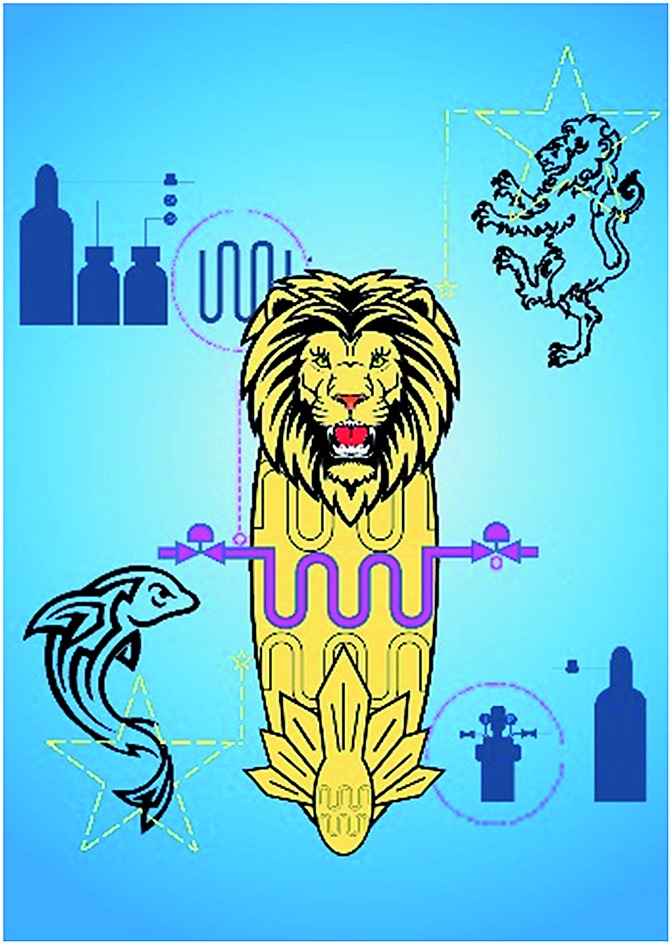
A “stop-flow” micro-tubing reactor system was designed for accelerating reaction discovery using flammable acetylene gas as the feedstock.

## Introduction

**Fig. 1:**
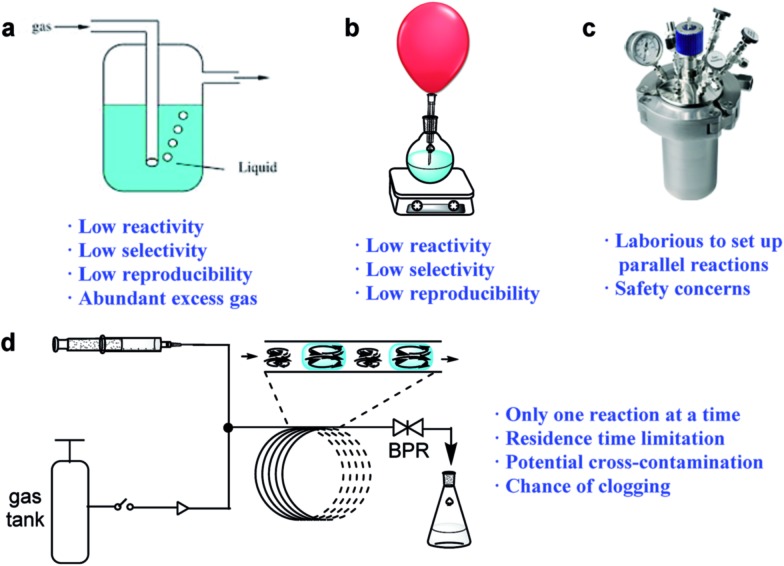
Methods for gas/liquid reaction screening: (a) gas bubbling, (b) gas balloons, (c) pressure gauges and (d) continuous-flow reactors. BPR: back-pressure regulator.

The discovery of new synthetic methodologies is the driving force for the development of organic chemistry,^1^ which normally requires the screening of numerous conditions. The use of reactive gases to generate valuable chemicals is of great concern from an economic and environmental standpoint, as these gases represent the most inexpensive chemical feedstocks and are readily removed after reactions, affording cleaner synthetic processes. However, there is a general reluctance to utilize reactive gases for new reaction discovery in research laboratories, which is mainly due to the lack of convenient, safe and effective ways to handle the reactive species. Gaseous reagents are conventionally introduced into the reaction mixture by bubbling (Fig. 1a) or balloons (Fig. 1b), and the reactions often suffer from low reactivity, poor reproducibility, and the wastage of excess gaseous reagents. High-pressure vessels (Fig. 1c) have been widely used to improve the reaction rate and selectivity of such reactions by enhancing the gas solubility and increasing the solvent’s boiling point.^2^ However, the methods that use these vessels are generally laborious, expensive, and are associated with safety concerns due to the risk of explosions. The normal stainless-steel high-pressure reactors are incompatible with photo-mediated reactions, and the development of new reactions using gaseous reagents under photo-mediated conditions is therefore limited, as these reactions usually require complicated and special apparatus.^3^

The emergence of continuous-flow technologies has provided excellent tools for gas/liquid transformations (Fig. 1d).^4^,^5^ When biphasic gas–liquid reactions are conducted in micro-flow reactors, the interfacial areas can greatly exceed those achieved in conventional batch reactors.^4^ The continuous-flow technologies have also provided effective methods for photo-synthesis utilizing gaseous reagents.^6^ Despite these successes, however, the screening of reaction parameters such as the reagents, catalysts, and solvents is not efficient when using continuous-flow techniques.^5^ A typical continuous-flow system usually does not allow for more than one reaction to be investigated at a time, which is not time-efficient. Moreover, the residence time is limited by the tubing-reactor size and therefore it cannot be too long. A uniform pressure is usually applied to a set of screened reactions as changing the pressure will disrupt the flow equilibrium. Additionally, cross-contamination is a potential problem for continuous screening, especially at high temperatures.^7^ Therefore, there is still a lack of methods that can provide a convenient and efficient screening process for continuous-flow synthesis. Due to its ease and better-defined scale-up routes,^8^ continuous-flow synthesis can be incorporated into virtually all of the phases of drug development. A platform suitable for reaction screening, that can be easily transferred into continuous-flow production, will therefore significantly shorten the processing time from lead discovery to manufacturing, and is highly desirable.

Here, we describe the development of a “stop-flow” micro-tubing (SFMT) parallel reactor system to address this gap which could represent an efficient and effective platform for gas/liquid reaction screening. By taking advantage of this technology, acetylene gas was subjected to transition-metal catalysis or photoredox conditions to construct valuable compounds. Acetylene is the simplest triple bonded hydrocarbon, which serves as an economic feedstock for a wide variety of commodity and speciality chemicals.^9^ However, commercial acetylene regulators do not exceed 30 psi at the outlet because acetylene can decompose explosively at high pressure.^10^ In this scenario, the gas/liquid surface interaction becomes especially important for achieving high reactivity and selectivity. We herein demonstrate the SFMT reactor-assisted development of three novel reactions using acetylene as the chemical feedstock, which are difficult or even impossible to access by conventional batch methods, and are not suitable for screening *via* continuous-flow techniques (requiring long reaction times).

## Results and discussion

### Development of the SFMT parallel reactor platform

**Fig. 2:**
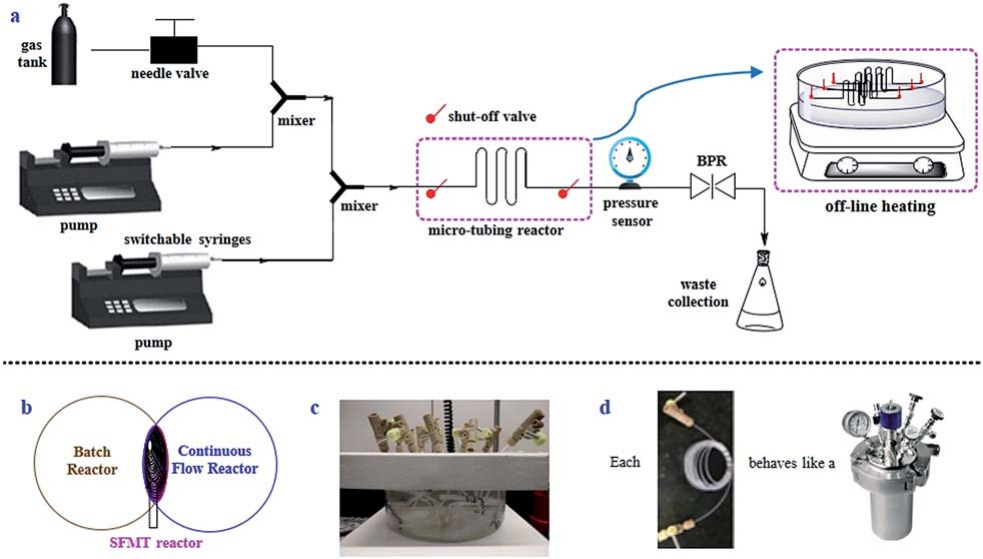
Design of the SFMT platform. (a) Schematic of the SFMT platform. (b) The SFMT platform represents a combination of batch and continuous-flow reactors. (c) Parallel SFMT reactors. (d) Each SFMT reactor behaves as a high-pressure reactor.

The SFMT reactor platform is a design adapted from continuous-flow systems with the addition of batch elements (Fig. 2b). Instead of flowing the reaction mixture continuously, the SFMT platform allows for the flow to be paused at will (Fig. 2a). Different from previous approaches,^11^ the SFMT system is based on a “switch in-and-out” approach through the use of two shut-off valves at each end of the micro-tubing reactor. Specifically, the SFMT platform provides a modular system, in which once a micro-tubing reactor is filled to the desired volume, both of the valves are closed, and the sealed reactor coil is disconnected from the system while maintaining a high pressure as determined by a back-pressure regulator (BPR).^12^ Other SFMT reactors can be similarly “switched in and out” using the same system. The filled reactors are then placed in a parallel arrangement under the determined reaction conditions until the desired reaction time has been achieved (Fig. 2c). Hence, each of the sealed micro-tubing reactors behaves like a high-pressure reactor (Fig. 2d).

We anticipate that the SFMT system is able to provide several benefits for gas/liquid reaction screening. First and foremost, the SFMT system exhibits many advantages through the use of micro-tubing reactors, such as excellent heat transfer, the ease of conducting reactions at high pressures with improved safety, significantly improved gas/liquid interfacial contact, an enhanced light irradiation efficiency,^13^ and a reduced amount of reagents required as well as waste produced. Although the SFMT reactor possesses a lower mixing efficiency in contrast to stirring in batch reactors, or circular flow patterns from Taylor flow,^14^ the excellent gas/liquid interfacial contact may still result in a good reaction efficiency. On the other hand, compared to continuous-flow techniques, reaction screening with SFMT reactors could effectively save time through parallel screening. The residence time is not limited by the reactor size and it can be as long as it needs to be. Moreover, each reactor can hold a different pressure and/or temperature during the screening.

The SFMT system was proven to work effectively at high pressures and high temperatures.^12^ Several reported reactions were performed with the SFMT system to investigate its feasibility through comparison with continuous-flow and batch reactors (ESI Table S1†).^12^ The tested reaction patterns included gas/liquid reactions, liquid/liquid biphasic reactions, photo-promoted reactions, and homogeneous reactions. For the gas/liquid transformations, the yields obtained in the SFMT reactors were better than those in the batch reactors, and were comparable with those in the continuous-flow reactors. For the homogeneous reactions, the yields in the SFMT reactors were in the same range as those from the batch or continuous-flow reactors. These comparison studies prove that the SFMT reactor is effective for reaction optimization/discovery with a wide range of reaction patterns. Most importantly, a successfully developed reaction in an SFMT reactor can be conveniently transferred to continuous-flow synthesis for large-scale production.

### Sonogashira coupling and fulvene synthesis using acetylene

Terminal alkynes are versatile intermediates for drug development and material synthesis. One intriguing way of generating such important moieties is the use of halogenated precursors and acetylene gas. However, constructing terminal alkynes directly from acetylene gas through Sonogashira coupling has proven to be particularly problematic,^15^ suffering from low selectivity between terminal and symmetric internal alkynes in conventional batch reactors.^16^ We anticipated that by using micro-tubing reactors, significant improvements in the reactivity and selectivity could be obtained due to the excellent gas/liquid interfacial contact in these reactors. However, the continuous-flow reactors were not efficient for optimization and substrate screening, as the coupling required a relatively long reaction time (around 2 hours). The SFMT reactor platform was deemed to be a more convenient and time-efficient tool. For instance, the use of SFMT reactors has allowed for the screening of 10 different conditions in less than 3 hours, whereas the continuous-flow reactor required more than 20 hours. A variety of conditions were thus investigated with the SFMT system using 4-iodoanisole **1a** and acetylene (ESI Table S2†).^17^ We were delighted to find that Sonogashira coupling gave terminal alkyne **2a** in 96% yield under ambient conditions using Pd(PPh_3_)_2_Cl_2_ and CuI as catalysts in DMSO, and only trace amounts of the undesired symmetric internal alkyne **3a** were detected (Table 1). This result was even more striking when contrasted with the data obtained from the batch reactor (total conversion 73%, 45% **2a** and 14% **3a**). Using the optimal conditions, the generality of this transformation was examined. As outlined in Table 1, a wide range of aryl iodides can be effectively converted to the corresponding terminal alkynes in good yields using the SFMT reactors (**2a** to **2l**), with the electron-rich substrates giving slightly better selectivities than the electron-poor substrates. Hetero-aryl iodides can also produce the desired mono-substituted alkyne in moderate to good yields and high selectivities (**2m** to **2o**). Less reactive aryl bromides required heating to obtain a high conversion, resulting in a significant amount of byproduct **3**. This was overcome by the *in situ* conversion of aryl bromides to aryl iodides in the batch reactor, followed by the direct introduction of this crude mixture into the SFMT system for Sonogashira coupling, which resulted in a slightly lower yield and selectivity.^12^ Interestingly, unlike aryl bromides, vinyl bromides can be directly used to access enyne products (**2p**) with high selectivity. More importantly, the developed optimal conditions can be applied to continuous-flow reactors to achieve gram-scale synthesis (*e.g.***2a**), indicating the potential for industrial-scale production through further scaling-out or numbering-up.^12^

**Table 1:** Utilization of acetylene as a feedstock in transition-metal catalysis^*a*^

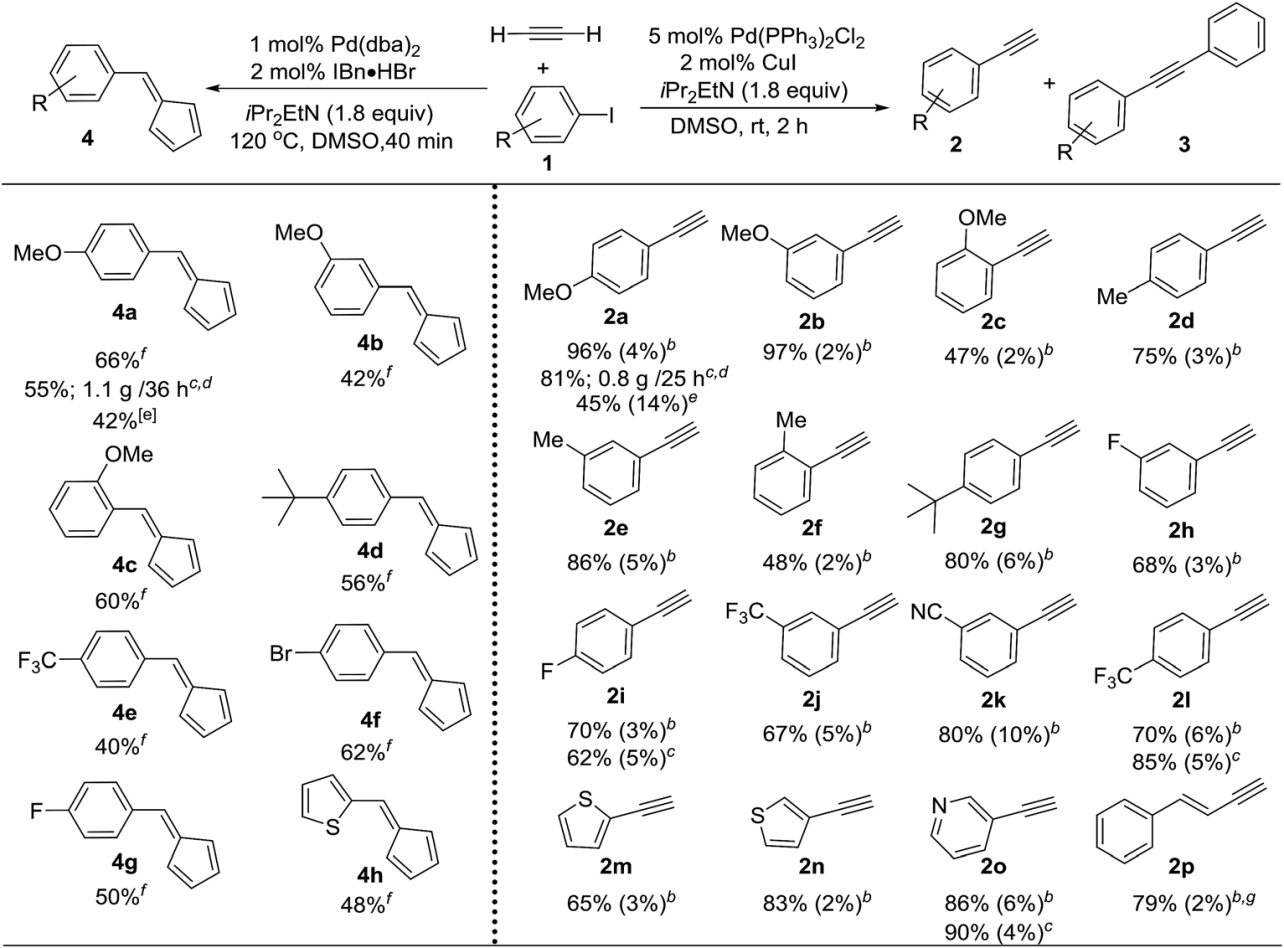

^*a*^The yield was determined using GC analysis of the crude mixture using an internal standard.

^*b*^The reactions were carried out in a SMFT reactor; the yield is shown as the yield of **2** (the yield of **3**).

^*c*^The reactions were carried out in a continuous flow reactor.

^*d*^The isolated yield.

^*e*^The reactions were carried out in a batch reactor.

^*f*^The reactions were carried out in a SMFT reactor.

^*g*^Vinyl bromide was used as the starting material.

Interestingly, when the reaction was conducted without a copper co-catalyst, Sonogashira coupling was not observed, and fulvene **4** was the only product that was detected.^12^ Three molecules of acetylene were incorporated in each molecule of fulvene **4**. Fulvenes have attractive applications in the synthesis of natural bioactive compounds and metallocene compounds,^18^ but the diversity of the structures of previously reported fulvenes is rather limited.^19^ This method provides a novel strategy for the synthesis of a wide range of mono-substituted fulvenes using inexpensive acetylene as a feedstock in a convenient manner.

### Fluorinated styrene-type product synthesis using acetylene under photo-redox conditions

Visible-light-mediated photoredox catalysis has attracted intense interest in the past ten years. The capacity of photocatalysts to act as strong single-electron oxidants or reductants upon light irradiation has enabled the development of transformations that are difficult or even unattainable *via* conventional pathways.^20^ However, the development of such kinds of reactions using gaseous reagents is hampered by the lack of appropriate convenient apparatus. In this context, we envision that the SFMT system provides an effective tool for developing visible-light promoted gas/liquid reactions and would be more suitable than the continuous-flow technique for screening, as visible-light promoted photoredox transformations require a considerable amount of time in many cases.^20^ Our initial screening using SFMT reactors for the photocatalytic vinylation of fluorinated aryl bromides using acetylene gas revealed that a range of catalysts could promote the reaction, except for the acridinium catalyst (ESI Table S4†). Ir(ppy)_2_(dtbbpy)PF_6_ was the most effective catalyst. Hünig’s base was found to be the most optimal base among the bases screened,^12^ with a combined yield of 94% for **6a** and **7a** in moderate selectivity (**6a** : **7a** = 1.8 : 1, Table 2, entry 1). This selectivity can be explained by the competing reaction pathways of the generated transient aryl radical, which can subsequently be added to acetylene to afford the desired vinyl radical species or capture a hydrogen atom from the *in situ* generated amine cation radical intermediate to give **7a**.^21^ Notably, when TEMPO was used as a stoichiometric additive (entry 2), the product selectivity was significantly enhanced, probably because TEMPO is able to abstract the H-atom from the amine cation radical and force the aryl radical to react with acetylene gas (ESI Fig. S9†).^12^ The use of more than one equivalent of TEMPO diminished the reactivity probably through the quenching of necessary aryl radical intermediates (entry 4). Importantly, when the same condition (entry 2) was applied in batch flasks, only trace amounts of the product were detected after a 3 hour reaction period (entry 5), which highlights the importance of the SFMT reactor in our study. An increase in reaction pressure to 20 psi slightly improved the product selectivity (entry 3). The optimized conditions were successfully extended to the vinylation of a variety of fluorinated aryl bromides. As shown in Table 2 (bottom), styrenes **6a–6k** were obtained in moderate to good yields. These obtained fluorinated styrene compounds have great promise for the facile preparation of fluorinated polymers. It is worth noting that in the midst of our study, the Weaver group published some nice work on the photocatalytic synthesis of alkenylated fluoroarenes *via* the formal hydrofluoroarylation of substituted alkynes from perfluoroarenes.^22^ However, to the best of our knowledge, our study represents the first example of the vinylation of aryl halogen compounds utilizing acetylene gas. The successful development of this reaction highlights the ease and effectiveness of SFMT systems for the screening of visible-light-mediated photoredox reactions using gaseous reagents.

**Table 2:** Utilization of acetylene as a feedstock in photo-redox catalysis^*a*^

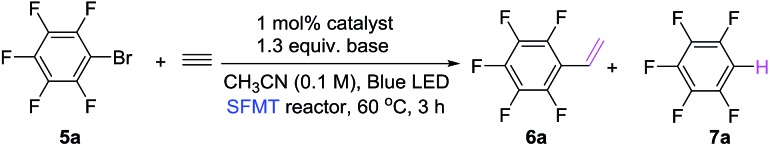					
Entry	Catalyst	Base	Conversion [%]	TEMPO	**6a** : **7a**
1	lr(ppy)_2_(dtbbpy)PF_6_	iPr_2_NEt	94	—	1.8 : 1
2	lr(ppy)_2_(dtbbpy)PF_6_	iPr_2_NEt	93	1 equiv.	3.2 : 1
**3** ^*b*^	**lr(ppy)**_**2**_ **(dtbbpy)PF**_**6**_	**iPr**_**2**_ **NEt**	**97**	**1 equiv.**	**3.6 : 1**
4	lr(ppy)_2_(dtbbpy)PF_6_	iPr_2_NEt	23	2 equiv.	2.3 : 1
5^*c*^	lr(ppy)_2_(dtbbpy)PF_6_	iPr_2_NEt	< 5	1 equiv.	—
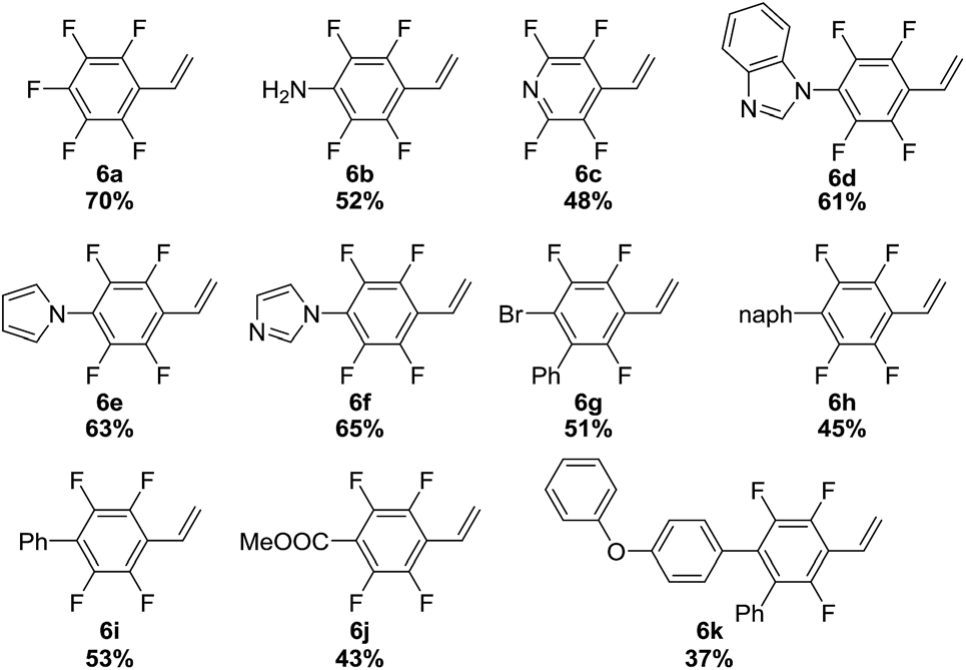					

^*a*^2 × 5-psi BPRs were used when setting up the reaction. The conversions and selectivities were based on the ^19^F-NMR analysis of the crude reaction mixture. Isolated yields.

^*b*^With 20-psi BPR.

^*c*^Conducted in the batch reactor.

## Conclusions

In summary, acetylene gas was utilized as a chemical feedstock to selectively produce value-added compounds including terminal alkynes, fulvenes, and styrene derivatives with the assistance of SFMT reactors. The developed transformations were ineffective in conventional batch reactors. The introduced SFMT platform has significantly accelerated the reaction development process and a successful reaction developed in SFMT reactors can be transferred to continuous-flow reactors for scaling-up the reaction. The use of SFMT reactors can therefore better support the synthetic community in taking full advantage of inexpensive gaseous reagents to build complex molecules. Owing to its ease of screening and safe operation at high temperatures and pressures, and its improved efficiency for gas/liquid and photo-promoted reactions, the SFMT platform promises to become an important method to supplement the current ways of setting up reactions in synthetic laboratories. Further optimization of the SFMT system for an automated system,^12^ analysis on the mixing and reaction kinetic behavior, and further applications for new reaction discovery are currently ongoing.

## Supplementary Material

Supplementary information
