# Supplementary material for: Reaction discovery using acetylene gas as the chemical feedstock accelerated by the “stop-flow” micro-tubing reactor system
Source: Chem Sci. 2017 Feb 27;8(5):3623–7. doi: 10.1039/c7sc00408g (PMC6094156; doi:10.1039/c7sc00408g)
Supplement: Supplementary file 1 [file SC-008-C7SC00408G-s001.pdf]

Electronic Supplementary Material (ESI) for Chemical Science.
This journal is © The Royal Society of Chemistry 2017

Reaction Discovery Using Acetylene Gas as the Chemical Feedstock Accelerated by the "Stop-Flow" Micro-Tubing Reactor System

Fei Xue,† Hongping Deng,† Chengwen Xue, Dara Khairunnisa Binte Mohamed, Karen Yuantang Tang, Jie Wu*

Department of Chemistry, National University of Singapore, 3 Science Drive 3, Republic of Singapore, 117543

†These authors contributed equally to this work.

Supporting Information

Table of Contents

1. General Experimental……………………………………………………………..…..SI-3

2. "Stop-Flow" Micro-Tubing (SFMT) Reactor Platform…..……………………………..SI-4

2.1 Graphical supporting information for the SFMT platform…..………………...…..SI-4

2.2 Graphical supporting information for individual components……….....………..SI-4

3. Mechanism Proposal……………………………………………………………………..SI-6

3.1 Proposed mechanism for fulvene synthesis using acetylene……………………..…..SI-6

3.2 Mechanism study for photo-promoted styrene derivative synthesis……….……...SI-6

4. Graphical Supporting Information for Reaction Screening Using the SFMT Platform………………………………………………………………………..………..SI-10

5. Feasibility Test for SFMT Reactors with Reported Transformations………………..……………………………………………………..……...SI-14

5.1 Pressure test in the SFMT reactor……………………………………..…..……...SI-14

5.2 Summary of comparison experimental results……………………………………SI-15

5.3 General procedure for batch, continuous-flow, SFMT reactions ……..………….SI-17

6. Sonogashira Coupling with Acetylene ………...…………………..………..……...SI-26

6.1 Reaction optimization………………………………………………....…..…..SI-26

6.2 General procedure for batch, SFMT, and continuous-flow reactions…...…...…..SI-27

6.3 Large-scale continuous production……………………..………………………...SI-28

6.4 One-pot Sonogashira coupling of bromoanisole with acetylene……….…..…...SI-29

6.5 New compound characterization……………………...……………..…………SI-30

7. Fulvene Synthesis ………...………………………………………….....…………...SI-36

7.1 Reaction optimization……………………………………..……...……..…..SI-36

7.2 General procedure for batch, SFMT, and continuous-flow reactions…...…...…..SI-37

7.3 Large-scale continuous production……………………..………..………..……...SI-39

7.4 New compound characterization……………………...……………….………SI-40

8. Visible-Light Promoted Synthesis of Styrene Derivatives Using Acetylene …..……....SI-43

8.1 SFMT Reactor Setup…….………..…………………………………....…..…..SI-43

8.2 Reaction optimization…………………………..…………………...……..…..SI-43

8.3 General procedure for batch, and SFMT, reactions…...……………………...…..SI-44

8.4 New compound characterization……………………...……………..…………SI-45

9. Trouble Shooting : Frequently Asked Questions ……………………...…....…..…...SI-49

9.1 The SFMT reactor platform…………………..……………………….……..…..SI-49

9.2 Sonogashira coupling…...……………………………………………...…..…..SI-52

9.3 Fulvene synthesis……………………..………………………....……....……...SI-53

9.4 Fluorinated styrene derivate synthesis……………………………………………SI-54

10. References and Notes……….......………..……………...……………......…...……...SI-54

11. NMR Spectra for New Compounds……….......………………………........…………...SI-55

1. General Experimental

Chemicals and solvents were purchased from commercial suppliers and used as received. Acetylene cylinder was purchase from Chem-Gas Pte Ltd (Singapore). ^{1}H NMR, ^{13}C NMR and ^{19}F NMR spectra were recorded on a Bruker ACF300 (300 MHz), AV-III400 (400 MHZ) or AMX500 (500 MHz) spectrometer. Chemical shifts were calibrated using residual undeuterated solvent as an internal reference ($CDCl_3$: 7.26 ppm ^{1}H NMR, 77 ppm ^{13}C NMR; 376 ppm ^{19}F NMR; CD_3CN: 1.94 ppm ^{1}H NMR, 1.3 ppm, 118.3 ppm ^{13}C NMR). Multiplicity was indicated as follows: s (singlet), d (doublet), t (triplet), q (quartet), m (multiplet), dd (doublet of doublet), bs (broad singlet). All high resolution mass spectra (HRMS) were obtained on a Finnigan/MAT 95XL-T spectrometer. All GC analysis was performed on Aglilent 7820A&5977E GC-MS. The 20 W LED bulb was purchased from Vtx Solution Pte Ltd (Singapore). The Blue LED strips (1 meter, 10 W; and 2 meter, 20 W) were purchased from Inwares Pte Ltd (Singapore). Further visualization was achieved by staining with iodine, potassium permanganate solution or Dragendorff's reagent followed by heating using a heat gun. Flash chromatography separations were performed on Merck 60 (0.040-0.063 mm) mesh silica gel. Purification of fluorinated styrene products was conducted on Recycling Preparative HPLC equipped with JAIGEL -2H columns (GPC, $CHCl_3$, as an eluent) from Japan Analytical Industry Co.,Ltd.

The Asia Syringe Pump was purchased from Syrris Company (UK) for continuous flow setup. The Logato$^{@}$ 200 series Syringe pumps were purchased from KD Scientific Inc. (Holliston, MA). The Harvard Syringe Pump was purchased from Harvard Apparatus (Holliston, MA). The Tefzel shut-off valves, HPFA and PFA micro tubings were purchased from IDEX Health&Science (Oak Harbor, WA). The stainless steel shut-off valves and stainless steel micro tubing was purchased from Swagelok (USA). The Digital Mass-Flow controller (SmartTrak 100) was purchased from Sierra Instruments. The membrane-based back pressure regulator was purchased from Zaiput Flow Technologies.

2. "Stop-Flow" Micro-Tubing (SFMT) Reactor Platform

2.1 Graphical supporting information for the SFMT platform

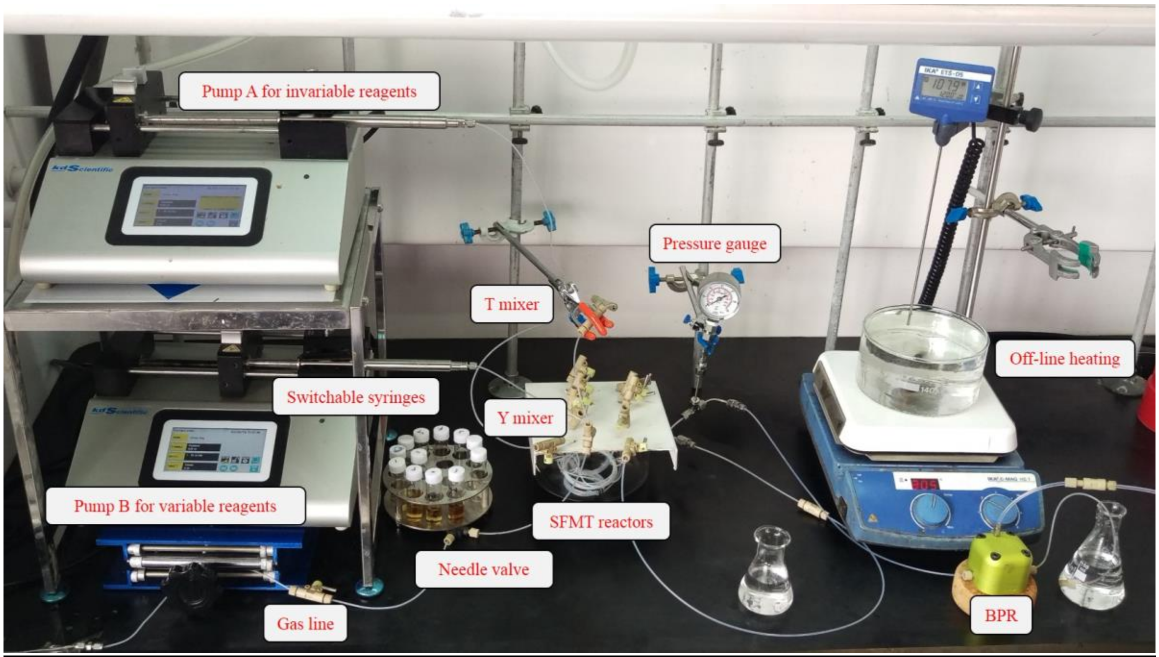

Figure S1. A general setup of the SFMT platform for the gas/liquid reaction. The system consists of two syringe pumps (**A** for invariable reagents, **B** for variable reagents; syringes containing different reagents/solvents/catalysts are switchable at **B**), one needle valve for controlling gas rate, a SFMT parallel reactor, one pressure gauge, one back-pressure regulator (BPR) and an off-line heating bath.

2.2 Graphical supporting information for individual components

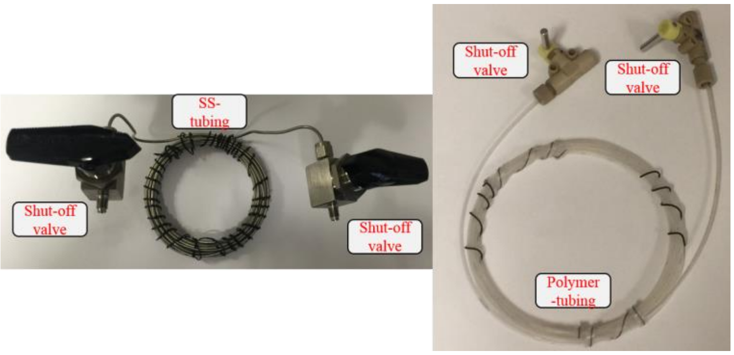

Figure S2. SFMT reactors. The left image shows a stainless-steel micro-tubing reactor whereas the right image shows a polymer micro-tubing reactor. The SS-tubing and ball valves were purchased from Swagelok (SS-T1-S-014-6ME, SS-41GS1). The polymer-tubing and shut-off valves were purchased from IDEX (1912L, P-732).

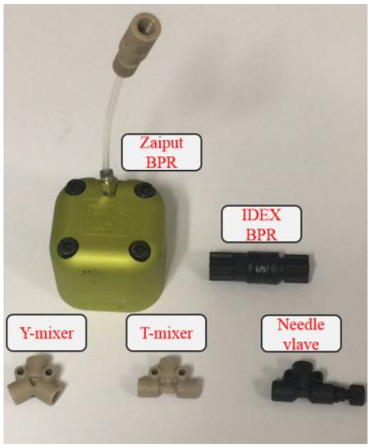

Figure S3. Parts required in the SFMT reactor system. The Zaiput BPR was purchased from Zaiput Flow Technologies. The IDEX BPR, Y-mixer, T-mixer, and needle valves were purchased from IDEX.

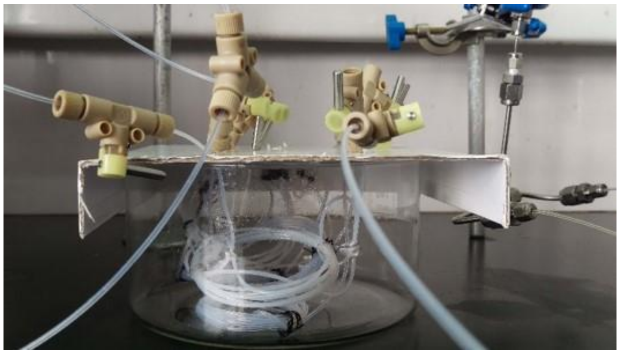

Figure S4. A close look at the SFMT parallel reactors.

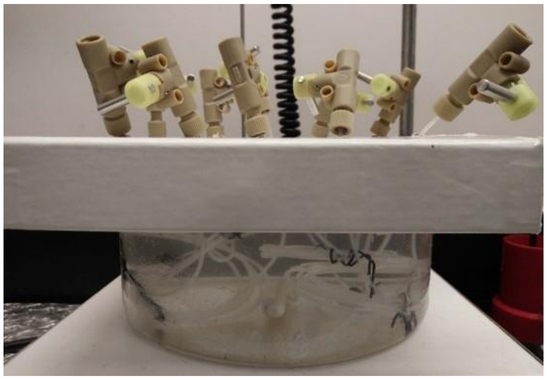

Figure S5. The SFMT parallel reactors in an off-line heating bath.

3. Mechanism Proposal

3.1 Proposed mechanism for fulvene synthesis using acetylene

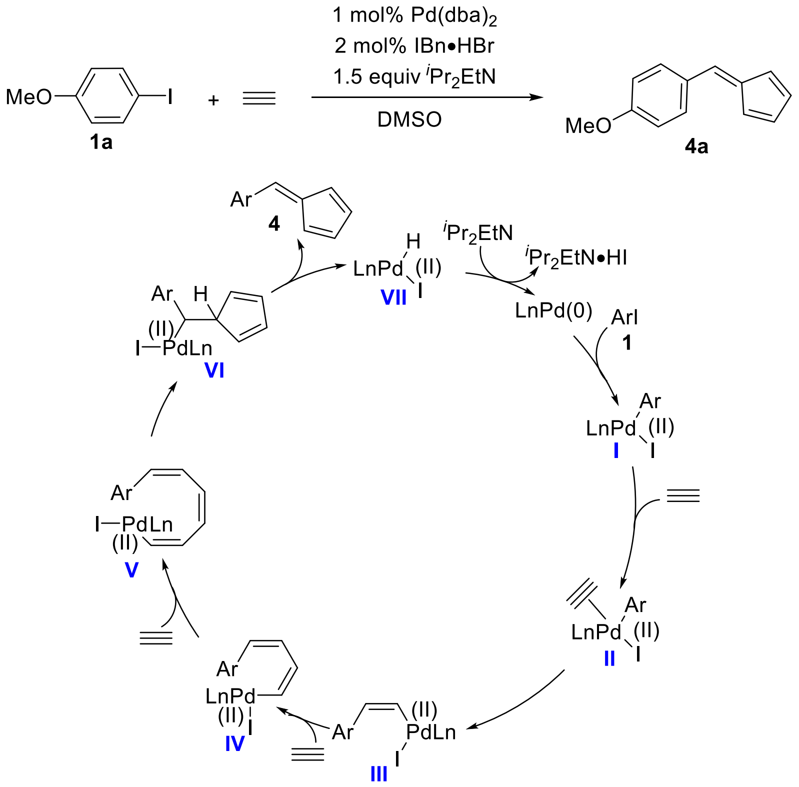

Figure S6. Proposed mechanism of Pd-catalyzed fulvene synthesis.

A possible mechanism for the fulvene formation is proposed in Figure S6. Oxidative addition of Pd(0) with aryl iodide forms Pd(II) iodide **I**, which will undergo *syn* addition to acetylene to give vinylpalladium iodide **III**. In the presence of excess acetylene, **III** is converted to triene **V** by two subsequent *syn* additions to acetylene. Subsequently, triene **V** undergoes a suprafacial insertion to give **VI**, followed by a β-hydride elimination to afford the fulvene product. Finally, a reductive elimination of Pd(II) **VII** in the presence of amine bases will regenerate the Pd(0) catalyst.

3.2 Mechanism study for photo-promoted styrene derivative synthesis

1) Does this transformation undergo an oxidative quenching cycle or reductive quenching cycle-based mechanism?

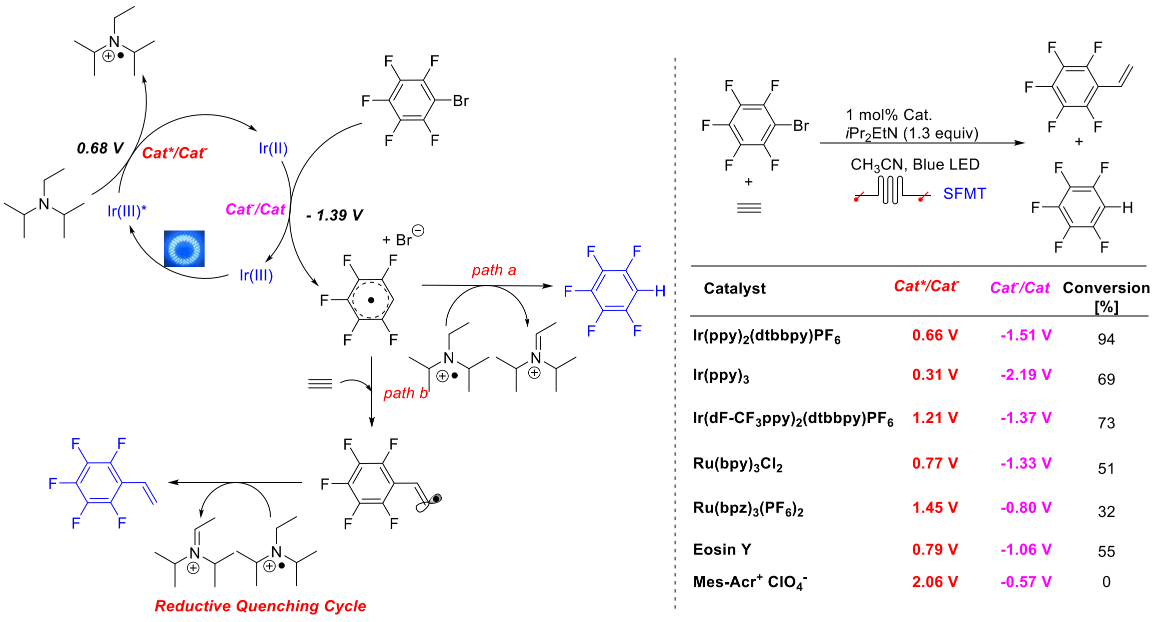

Catalyst	Cat^*/Cat^-	Cat^-/Cat	Conversion [%]
$Ir(ppy)_2(dtbbpy)PF_6$	0.66 V	-1.51 V	94
$Ir(ppy)_3$	0.31 V	-2.19 V	69
$Ir(dF\text{-}CF_3ppy)_2(dtbbpy)PF_6$	1.21 V	-1.37 V	73
$Ru(bpy)_3Cl_2$	0.77 V	-1.33 V	51
$Ru(bpz)_3(PF_6)_2$	1.45 V	-0.80 V	32
Eosin Y	0.79 V	-1.06 V	55
$Mes\text{-}Acr^+ ClO_4^-$	2.06 V	-0.57 V	0

Figure S7. Proposed reductive quenching cycle-based mechanism.

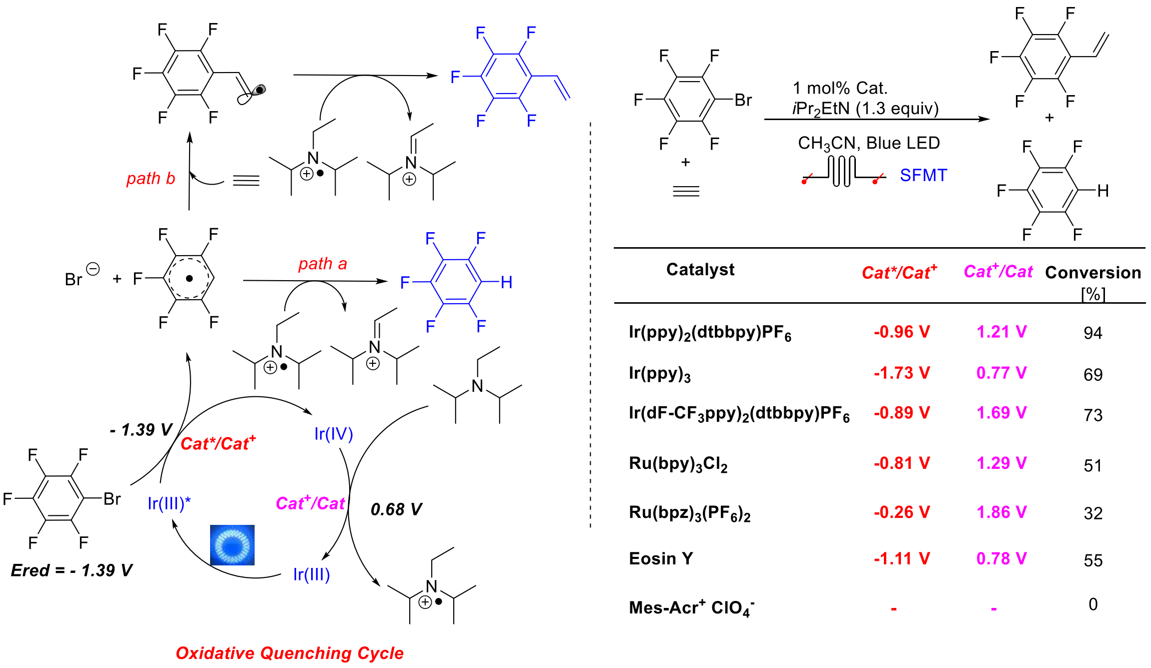

Catalyst	Cat^*/Cat^+	Cat^+/Cat	Conversion [%]
$Ir(ppy)_2(dtbbpy)PF_6$	-0.96 V	1.21 V	94
$Ir(ppy)_3$	-1.73 V	0.77 V	69
$Ir(dF\text{-}CF_3ppy)_2(dtbbpy)PF_6$	-0.89 V	1.69 V	73
$Ru(bpy)_3Cl_2$	-0.81 V	1.29 V	51
$Ru(bpz)_3(PF_6)_2$	-0.26 V	1.86 V	32
Eosin Y	-1.11 V	0.78 V	55
$Mes\text{-}Acr^+ ClO_4^-$	-	-	0

Figure S8. Proposed oxidative quenching cycle-based mechanism.

The reductive potential of pentafluorophenylbromide is reported ~ -1.39 V vs SCE.[1] The redox potential of iPr$_2$EtN$^{\cdot+}$/iPr$_2$EtN is ~ +0.68 V vs SCE. As shown in Figure S7, the reductive quenching cycle involves a photoinduced electron transfer from iPr$_2$EtN to the

excited photocatalyst and subsequent reoxidation of the generated Cat^- by bromopentafluorobenzene. The reduced fluorinated arene cleaves the C_{Ar}–Br bond yielding the pentafluorophenyl radical, which can be quenched by acetylene (path b) to generate the desired vinyl radical intermediate, or quenched by the amine radical cation (path a) to give debromination byproduct. However, the oxidative quenching cycle is based on photo-induced electron transfer from excited photocatalyst to bromopentafluorobenzene (Figure S8). The reaction proceeded with most of the photocatalysts screened despite the strong oxidative catalyst Mes-$Arc^+ClO_4^-$. Comparing the redox potential of catalyst triplet states (Cat^*/Cat^-) and $i$$Pr_2$EtN, only $Ir(ppy)_3$ is not thermodynamically feasible for the reductive quenching cycle. The next step, reoxidation of the catalyst by the bromopentafluorobenzene ($E_{1/2}^{red}$ of Cat^-/Cat vs $E_{1/2}^{red}$ of bromopentafluorobenzene = -1.39 V), only $Ru(bpz)_3(PF_6)_2$ is not thermodynamically feasible. However, in the oxidative quenching cycle, comparing the redox potential of catalyst triplet states (Cat^*/Cat^+) and bromopentafluorobenzene, only $Ir(ppy)_3$ is thermodynamically favorable, which does not correspond to the experimental results. Therefore, this transformation is more likely proceeded through the reductive quenching cycle. It should be noted, however, that the possibility of the reaction taking place via both reductive and oxidative quenching cycles simultaneously cannot be ruled out.

2) What is the role of TEMPO that leads to an improvement of the product selectivity?

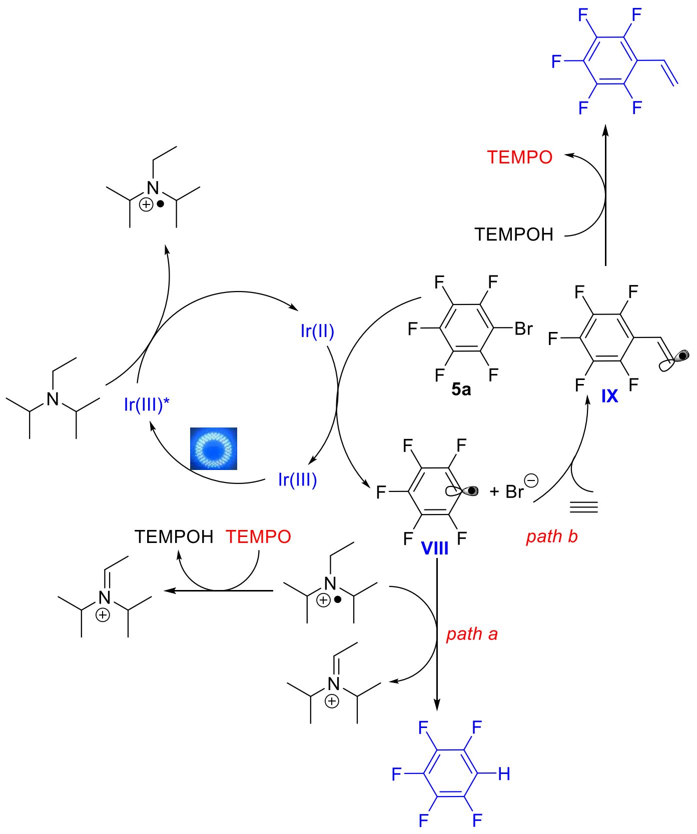

Figure S9. Proposed mechanism for photo-promoted styrene derivative synthesis.

As shown in Figure S9, when one equiv TEMPO was used as an additive, the product selectivity was significantly enhanced, probably because that TEMPO can abstract the H-atom from the amine cation radical and force the aryl radical **VIII** to undergo path *b* to react with acetylene gas.

3) Trap the pentafluorophenyl radical by 2,6-di-tert-butyl-4-methylphenol

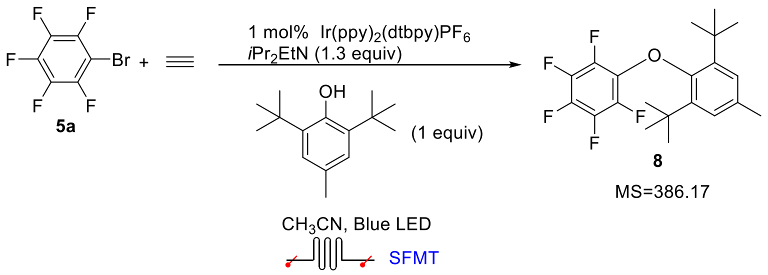

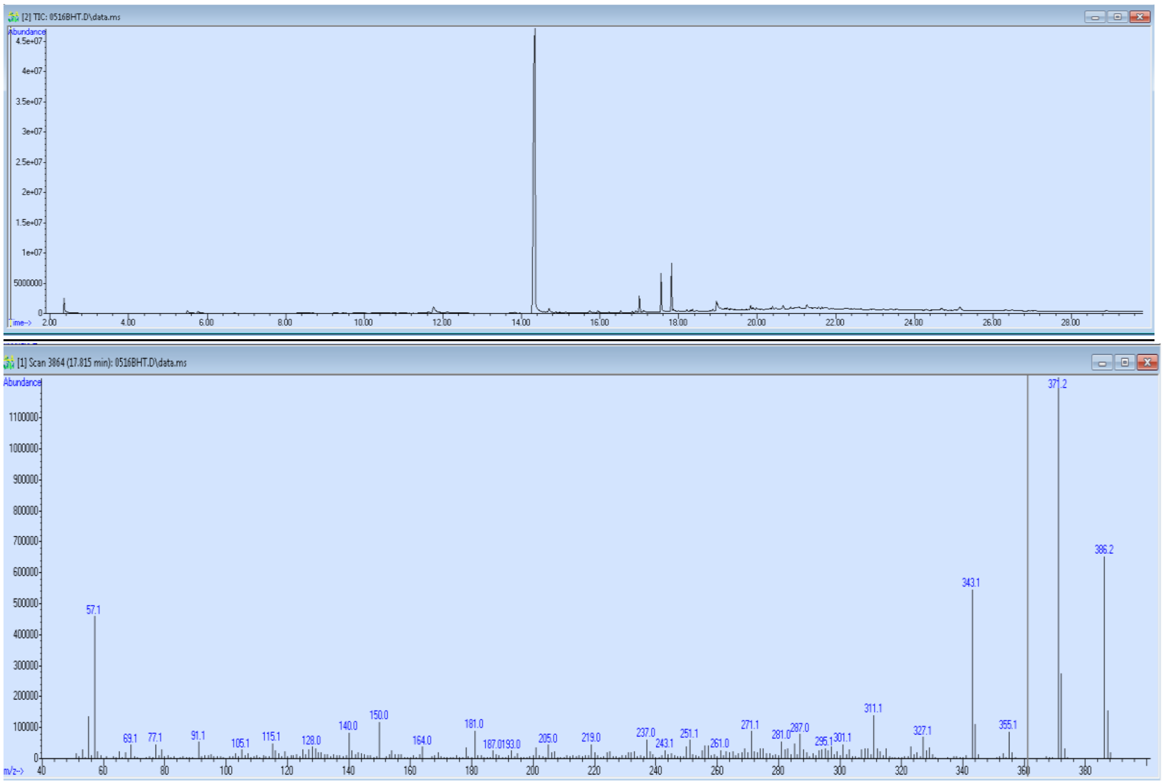

Figure S10. Trapping reaction intermediates with 2,6-di-tert-butyl-4-methylphenol.

2,6-Di-tert-butyl-4-methylphenol was added to attempt to trap the reaction intermediates. As indicated by GC-MS analysis of the crude reaction mixture, compound **8** was produced, which indicated the presence of aryl radical **VIII** in the reaction process.

4. Graphical Supporting Information for Reaction Screening Using the SFMT Platform

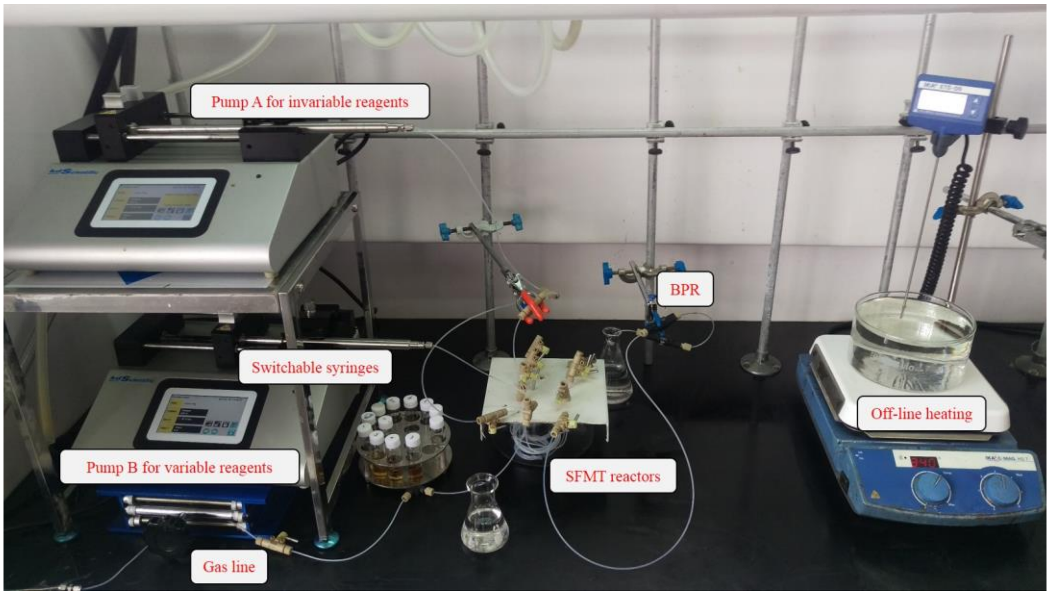

Step 1: Setup of the SFMT platform. Fill syringe A with invariable reagents. Fill syringe B with screening targets (variable reagents, catalysts, or solvents, etc).

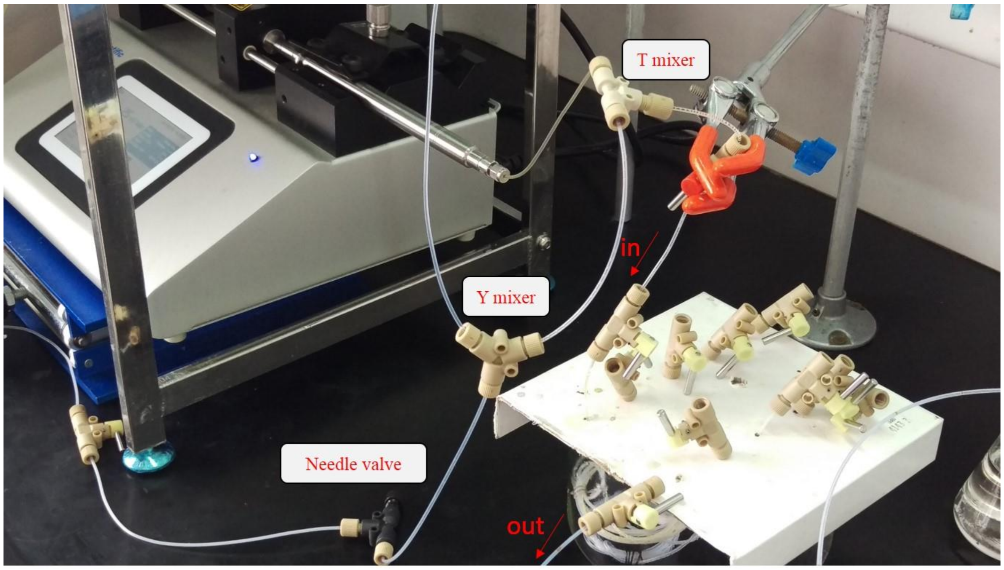

Step 2: Start pumping, and open the needle valve to adjust the gas bubbles. Fill in the first micro-tubing reactor with starting gas/liquid reagents.

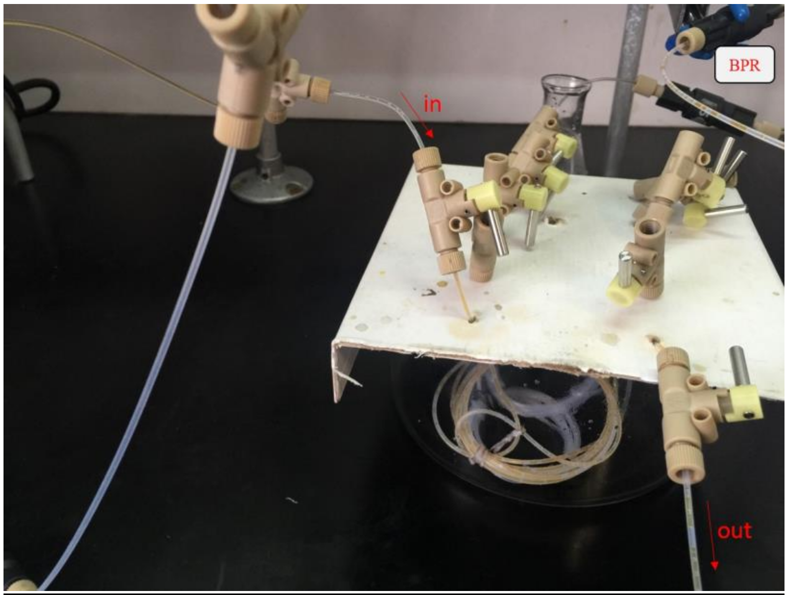

Step 3: Fill in the first micro-tubing reactor and let the reagent mixture go through the BPR.

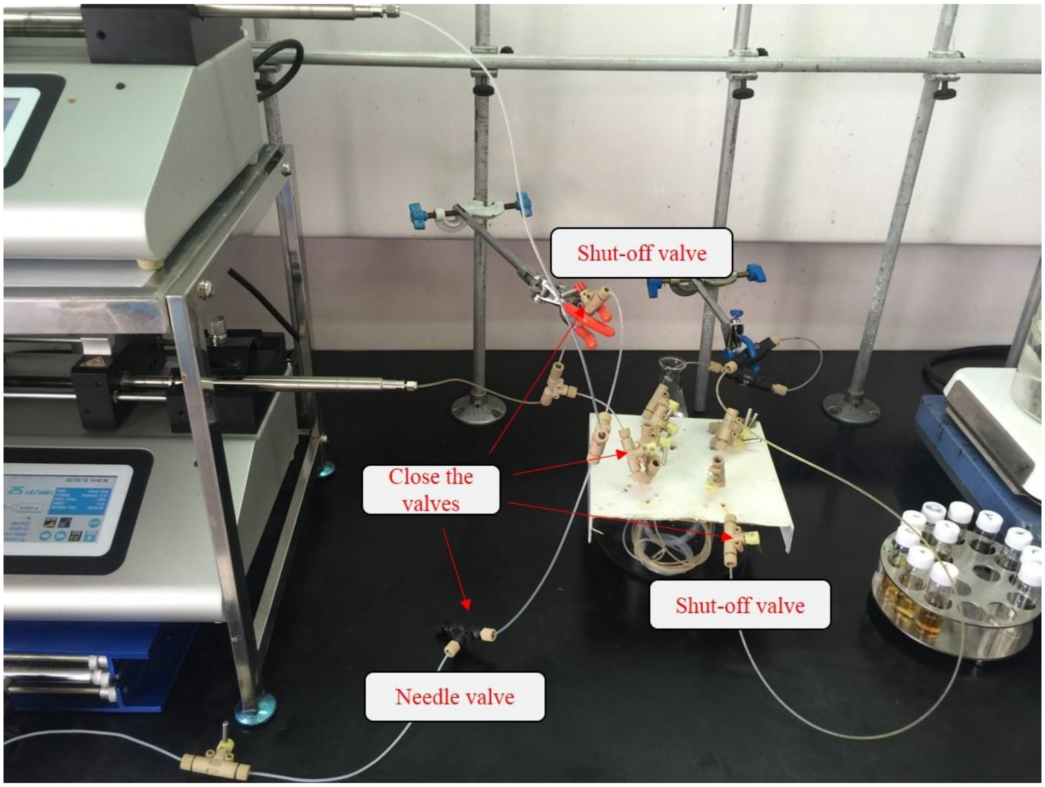

Step 4: Close the shut-off valves. Stop pumping and close the gas needle valve.

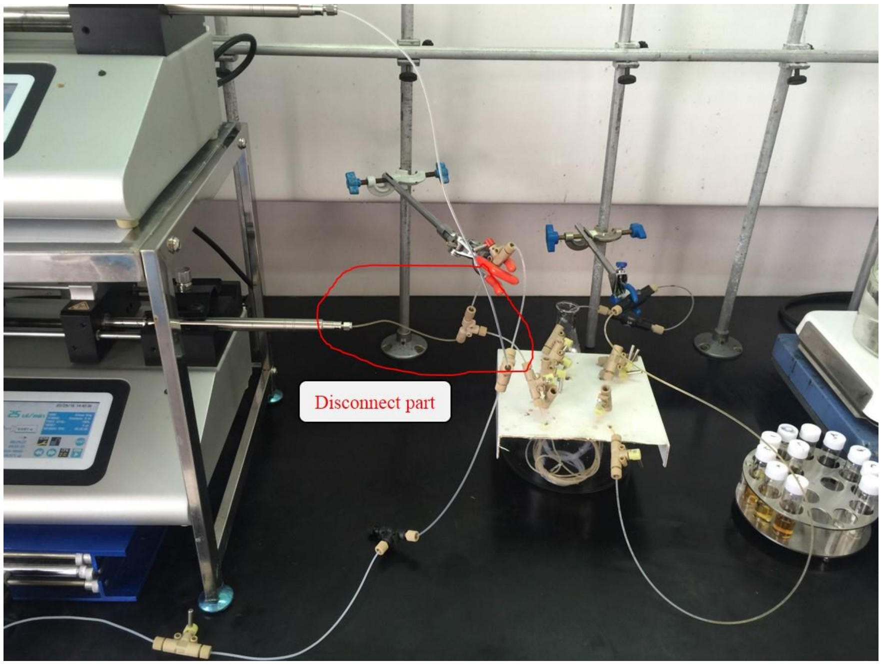

Step 5: Disconnect the switchable syringe and jointers. Switch to the syringe containing next screening reagents/catalyst/solvents.

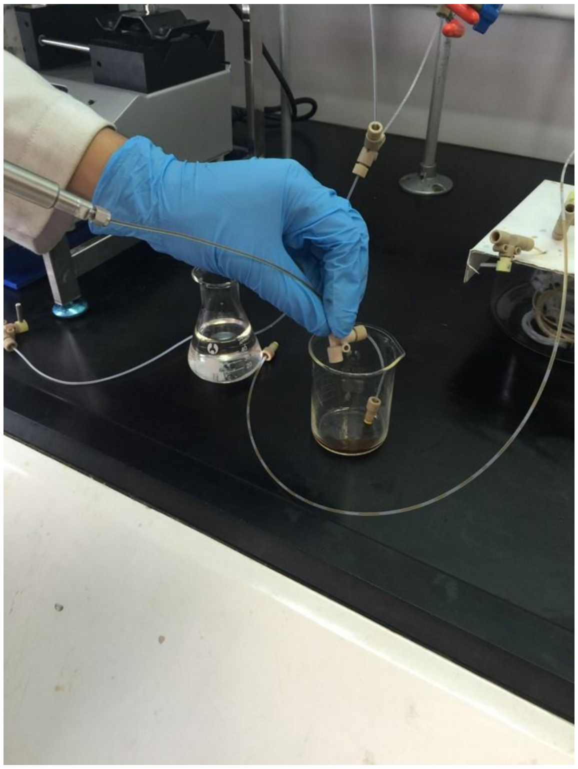

Step 6: The changed parts are rinsed with the washing solvent.

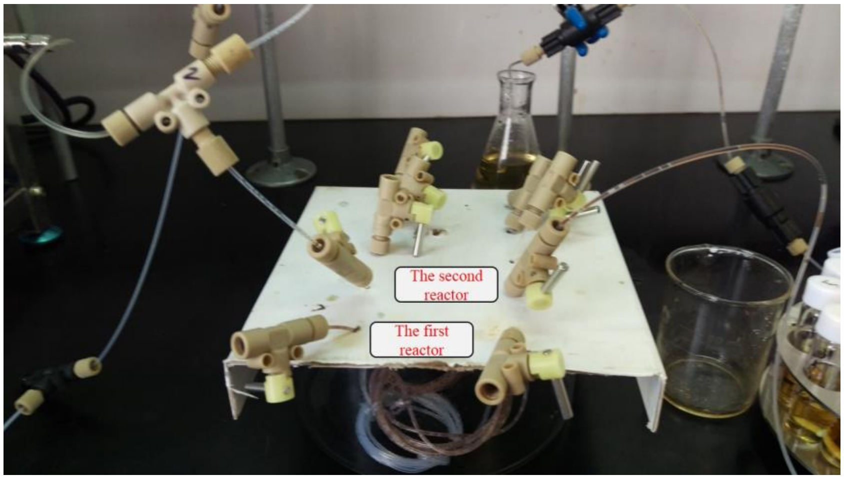

Step 7: The second reactor is filled and closed in the same way.

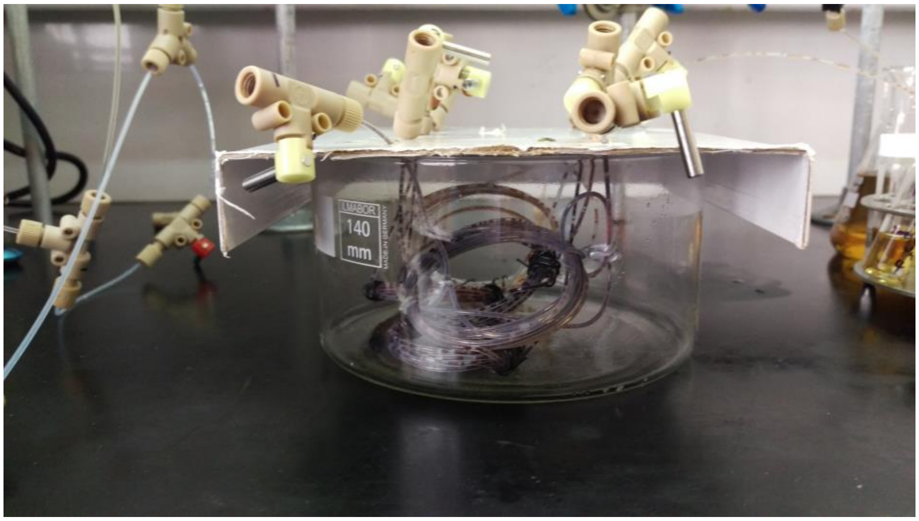

Step 8: All reactors are filled and disconnected from the flow system.

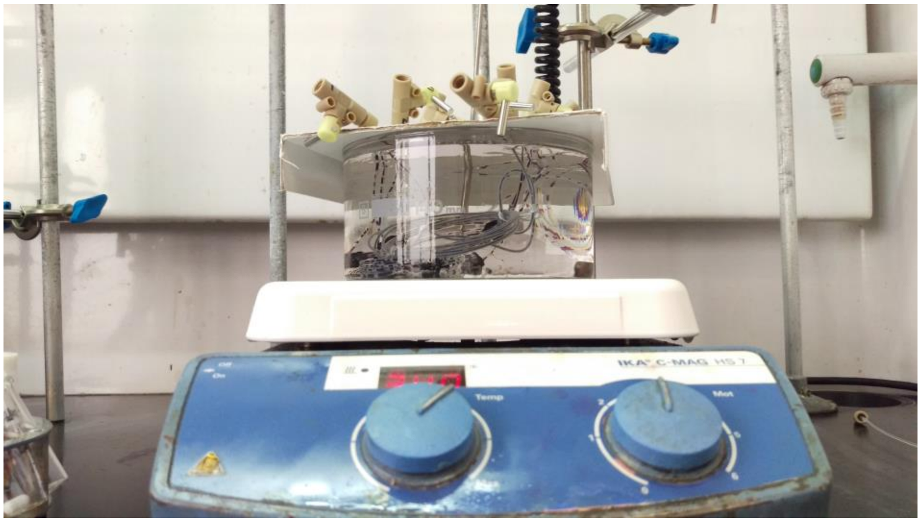

Step 9: The SFMT reactors are moved to an off-line heating bath with desired temperature.

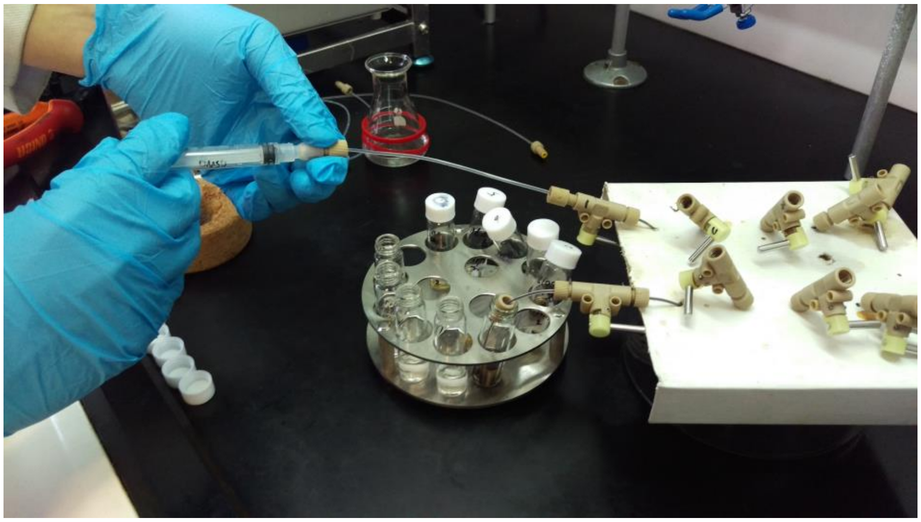

<u>Step 10</u>: After desired reaction period, the crude product mixtures are rinsed out to collection vials.

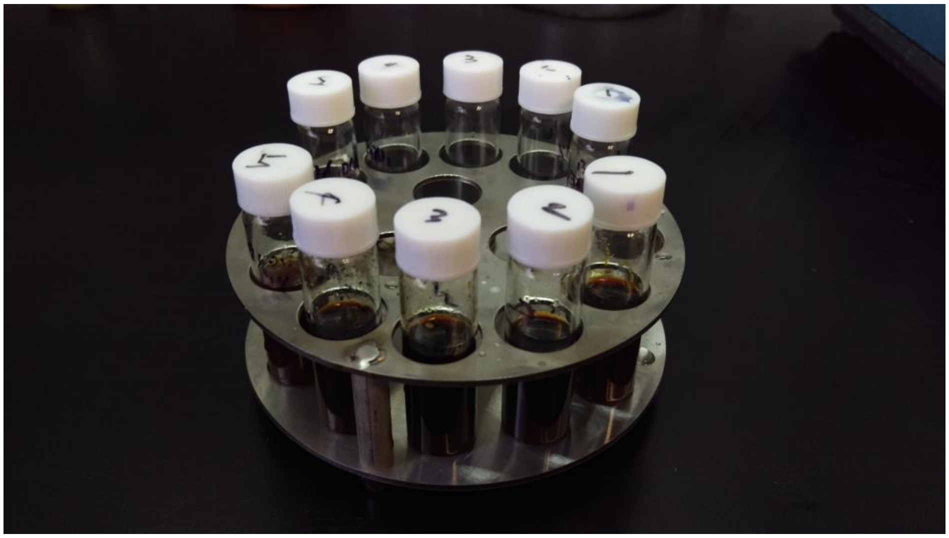

<u>Step 11</u>: Collected crude products will be subjected to GCMS and ^{1}HNMR for analysis.

5. Feasibility Test for SFMT Reactors with Reported Transformations

<u>5.1 Pressure test in the SFMT reactor</u>

The first pressure test conducted was to examine whether the SFMT reactor can maintain a high pressure during heating. As shown in Figure S11a, an SFMT reactor (made by HPFA, 1/16 inch O.D., 0.03 inch I.D., 100 cm) was connected to a pressure gauge to indicate the pressure inside the reactor. A gas-liquid flow of DCM and argon was pumped through the tubing, using a 100 psi back-pressure regulator to pressurize the system (Figure S11a and b). When the SFMT reactor was heated at 100 ^{o}C, the pressure of SFMT reactor was slightly increased from 100 psi to 125 psi. After heating for 1 hour, this pressure was maintained for the duration of the heating period (Figure S11c), and the gas/liquid slug shape was not changed.

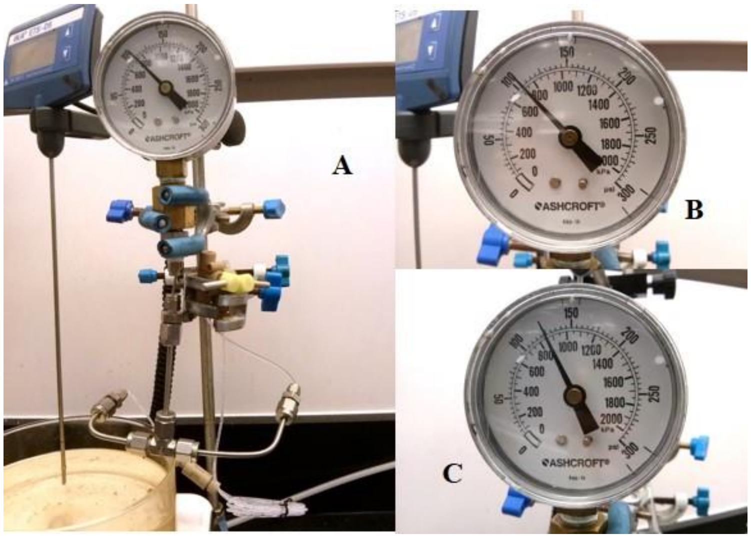

Figure S11. Pressure test in the SFMT reactor. a, Setup for the SFMT reactor pressure study. **b,** Pressure before heating. **c,** Pressure after heating for 1 hour.

The ability of the SFMT platform to hold pressure was further tested by increasing the pressure to 1000 psi. Since the pressure limit of the Tefzel shut-off valves were 500 psi, stainless steel valves were used for this test. With a 500 psi and two 250 psi BPRs, for a total of 1000 psi back-pressure, water was pumped into the SFMT reactor (made by HPFA tubing, 1/16 inch O.D., 0.03 I.D., 100 cm) until the pressure gauge read 1000 psi. The valves were then closed, and the tubing reactor was left in room temperature for several hours. It was observed that the pressure decreased slowly to 860 psi within 2 hours and maintained at 860 psi for another 2 hours. This pressure drop can be explained due to the dissolution of pressurized air, which was left in the pressure gauge, into water at high pressure (Figure S12). However, the study supported that the SFMT system can work effectively at pressure as high as 800 psi.

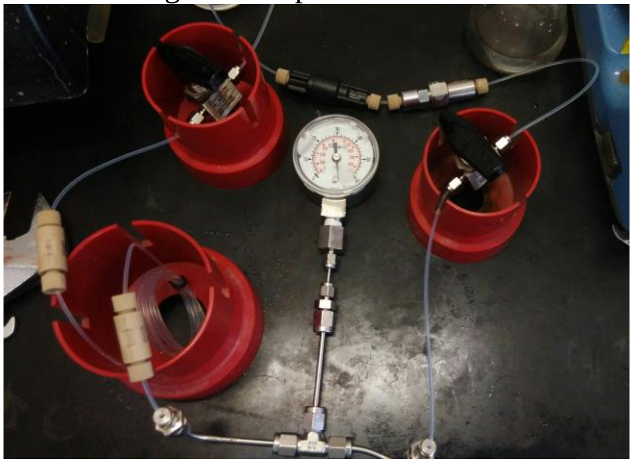

Figure S12. SFMT reactor pressure study at high pressure.

5.2 Summary of comparison experimental results

The following reported reactions were tested as template reactions to investigate the versatility and feasibility of the SFMT reactor platform (Scheme S1). Reactions were

attempted to perform in batch, continuous-flow and stop-flow reactors with the same reaction parameters (temperature, pressure, concentration, and reaction time) for comparison. The results are summarized in Table S1.

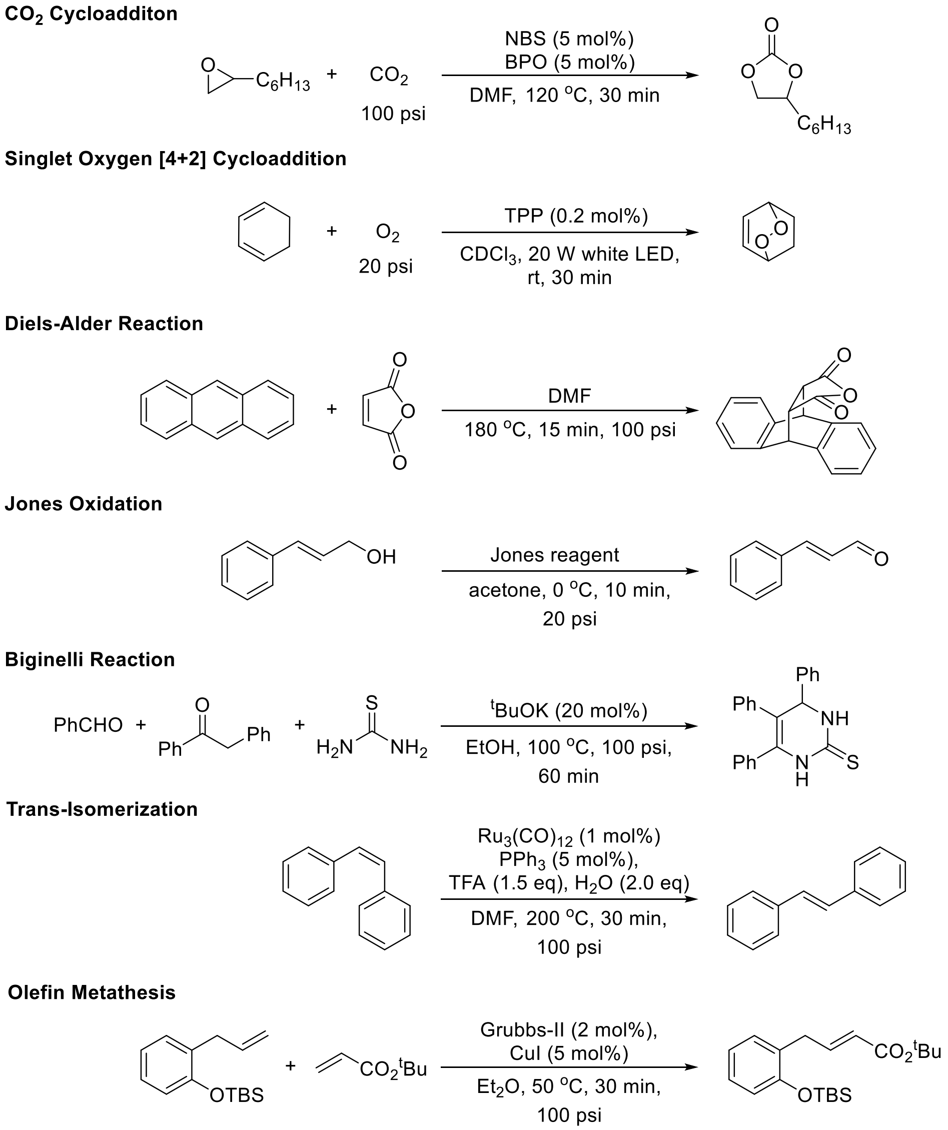

Scheme S1. Template reactions for comparison studies.

As shown in Table S1, reaction patterns tested included gas/liquid reactions, liquid/liquid heterogeneous reactions, photo-promoted reactions, and homogeneous reactions. In our hands, reactions in SFMT reactors gave comparable or better yields than those in batch reactors, and were slightly lower than those in continuous-flow reactors, except for the Biginelli reaction. For the Biginelli reaction (entry 5), the product was solid and was insoluble in ethanol, which was found to accumulate in the back-pressure regulator (BPR), causing blockage. In comparison, the solid product was easily removed from the SFMT reactor simply by pressurizing the tubing after the reaction. These comparison studies prove that the SFMT reactor is effective with a wide range of reaction patterns and is instructive enough for reaction optimization/discovery. It also indicates that a

successfully developed reaction in the SFMT reactor can be conveniently transferred to continuous-flow synthesis for large-scale production.

Table S1. Comparison of batch, continuous-flow, and SFMT reactors.

Entry	Reaction	Yield [%][a]			Reaction Type	Remarks
		Batch	Continuous -Flow	Stop-Flow		
1	CO_2 cycloaddition	4	64	48	gas/liquid	high-temp/ high-pressure
2	[4+2] with singlet oxygen	49	65	62	gas/liquid	photo-promoted
3	DA-reaction	11	56	53	homogeneous	high-temp/ high-pressure
4	Jones Oxidation	70[b]	76 [b]	77[b]	organic/inorganic	strong oxidant
5	Biginelli reaction	67	49	70	homogeneous	multi-component
6	trans-isomerization	72	73	62	homogeneous	high-temp/ high-pressure
7	olefin metathesis	60	73	68	homogeneous	reversible reaction

[a] All yields were determined by ^{1}H NMR spectrum analysis with 1,3,5-trimethoxybenzene or mesitylene as an internal standard. [b] Isolated yields.

5.3 General procedure for batch, continuous-flow, SFMT reactions

1) General setup for SFMT reactions

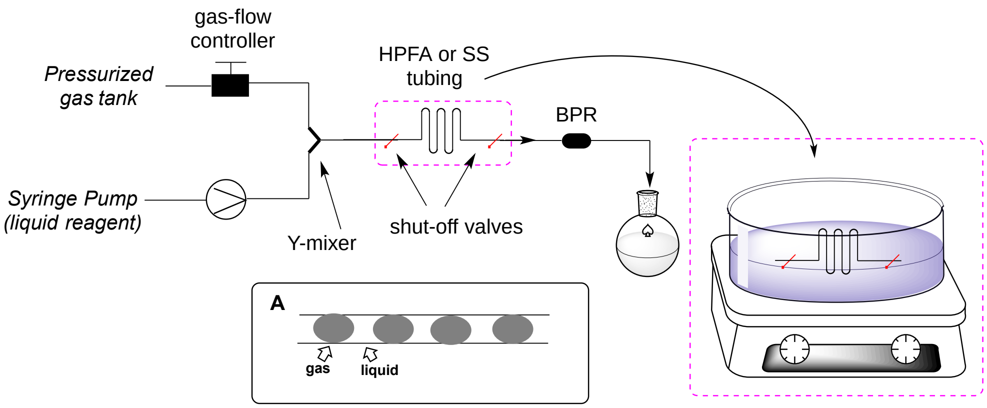

Figure S13. Schematic setup of gas-liquid stop-flow reactions.

Setup A: As shown in Figure S13, a stainless steel syringe was fixed to a syringe pump and a pressurized gas cylinder was attached to a Y-mixer through HPFA tubing (O.D. 1/16”, I.D. 0.03”) as inlets. The outlet of Y-mixer was connected to the reactor (HPFA or stainless steel, O.D. 1/16”, I.D. 0.03”) and the gas-line. The ends of the reactor were connected with two shut-off valves. The outlet of reactor was connected to a back-

pressure regulator (BPR). The gas cylinder was pressurized to ~20 psi higher than the desired back-pressure of the system. A stainless steel syringe was filled with the homogeneous mixture of substrates, catalysts, additives and solvents, and attached to the flow apparatus (syringe pump). The flow apparatus itself was set up with a certain flow rate, and flow rate of gas was adjusted to create 1:1 liquid/gas plugs. After filling the reactor and approximately 2 mins of equilibration, the valves were closed and the tubing reactor was placed into the hot oil bath or under a light source for further reaction.

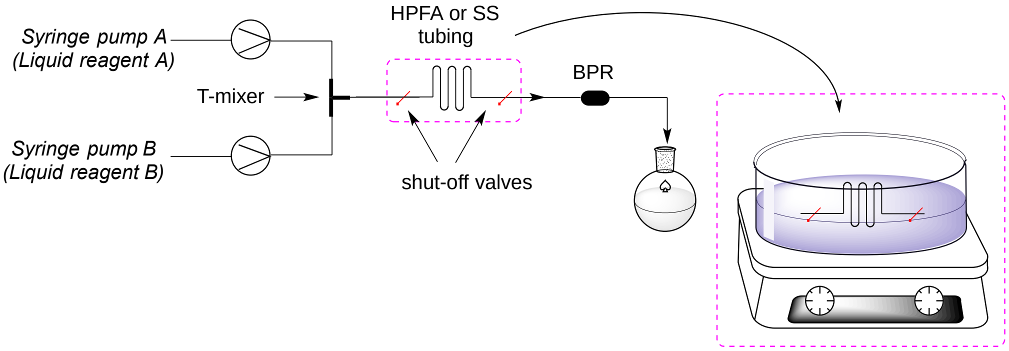

Figure S14. Schematic setup of liquid-liquid stop-flow reactions.

Setup B: As shown in Figure S14, two stainless steel syringes filled with a solution of substrates A and a solution of B separately were fixed to two syringe pumps (pump A with substrate A, pump B with substrate B). The syringes were attached to a T-mixer through an HPFA tubing (1/16”, I.D. 0.03”). The outlet of T-mixer was connected to the reactor (HPFA or stainless steel, 1/16”, I.D. 0.03”). The ends of the reactor were attached to two shut-off valves. The outlet of reactor was connected to a back-pressure regulator (BPR). Each syringe pump was set up with different flow rates, and the substrates were introduced into the flow system. After filling the tubing reactor and approximately 2 mins of equilibration, the valves were closed and the tubing reactor was placed into the hot oil bath or under a light source for further reaction.

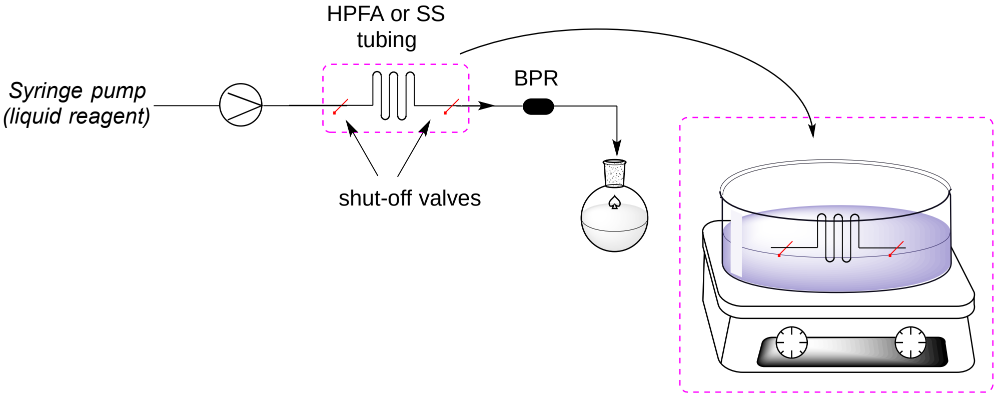

Figure S15. Schematic setup of one-stream stop-flow reactions.

Setup C: As shown in Figure S15, a stainless steel syringe filled with the reaction mixture was fixed to a syringe pump and directly attached to the reactor (HPFA or stainless steel, 1/16”, I.D. 0.03”). The ends of the reactor were attached to two shut-off valves. The outlet of reactor was connected to a back-pressure regulator (BPR). A stainless steel syringe was filled with the homogeneous mixture of substrates, catalysts,

additives and solvents, and attached to the flow apparatus (syringe pump). The flow apparatus was set up with a determined flow rate before the reaction mixture was pumped through the flow system. After filling the reactor and approximately 2 mins of equilibration, the valves were closed and the tubing reactor was placed into the hot oil bath or under a light source for further reactions.

2) General setup for continuous-flow reactions

All continuous flow reactions were set up in the same manner as the "stop-flow" setups without the use of shut-off valves at the ends of the reactors. The reactors (HPFA or stainless steel) were attached directly to the main system, and directly immersed in an oil bath, ice bath or exposed to a light source. The gas cylinder was pressurized to ~20 psi higher than the desired back-pressure of the system. A stainless steel syringe was filled with the homogeneous mixture of substrates, catalysts, additives and solvents, and attached to the flow apparatus (syringe pump). The tubing reactor was placed into the oil bath or ice bath or under a light source. The flow apparatus itself was set up at a certain flow rate (according to t_R and the tubing size), and flow rate of gas was adjusted to create 1:1 liquid/gas plugs. After approximately three residence time period to equilibrate, the solution was collected for the desired time for further work-up.

3) General procedure for template reactions in batch, continuous-flow and stop-flow

CO_2 Cycloaddition Reaction[2]

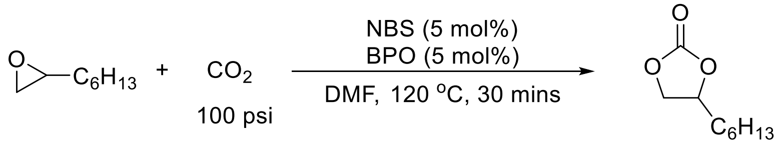

Batch Reaction

Under CO_2 atmosphere, a Parr reactor charged with a solution of 1,2-epoxyoctane (1.5 mL, 10 mmol), NBS (89 mg, 0.5 mmol), benzoyl peroxide (120 mg, 0.5 mmol), mesitylene (400 mg, 3.3 mmol) in DMF (4.5 mL) was pressurized to 100 psi with CO_2. The reaction mixture was heated to 120 °C and stirred for 30 mins. After reaction was completed, cooled in an ice bath, and pressure was released slowly. The reaction mixture was then poured into water and extracted twice with diethyl ether. The combined organic phase was washed with water, dried over sodium sulfate, filtered, and concentrated under reduce pressure with the temperature below 20 °C to avoid loss of internal standard mesitylene, afforded the product in 4% yield according to crude ^{1}H NMR analysis.

Continuous-Flow Reaction

The CO_2 gas cylinder was pressurized to ~20 psi higher than the desired back-pressure of the system (120 psi). A 10 mL volumetric flask was charged with a solution of 1,2-epoxyoctane (1.5 mL, 10 mmol), NBS (89 mg, 0.5 mmol), benzoyl peroxide (120 mg, 0.5 mmol), mesitylene (400 mg, 3.3 mmol) in anhydrous DMF (5.0 mL). An 8 mL, stainless steel Harvard Apparatus syringe was filled with the solution and then attached to the flow apparatus according to setup A. The tubing reactor (stainless steel, 1/16", I.D. 0.03", 762 cm, volume = 3.47 mL) was placed into the oil bath at 120 °C. The flow apparatus itself

was set up with t_R = 30 mins, flow rate = 22.3 µL/min and flow rate of CO_2 was adjusted to create 1:1 liquid/gas plugs. After approximately 1.5 hour to equilibrate, the solution was collected for 30 mins and quenched with distilled water, extracted twice with diethyl ether and washed with water. The organic phase was combined and the solvent was removed under reduced pressure with the temperature below 20 °C to avoid loss of internal standard mesitylene, indicated the desired product in 64% yield according to crude ^{1}H NMR analysis.

Stop-Flow Reaction

The CO_2 gas cylinder was pressurized to ~20 psi higher than the desired back-pressure of the system (120 psi). A 10 mL volumetric flask was charged with a solution of 1,2-epoxyoctane (1.5 mL, 10 mmol), NBS (89 mg, 0.5 mmol), benzoyl peroxide (120 mg, 0.5 mmol), mesitylene (400 mg, 3.3 mmol) in anhydrous DMF (5.0 mL). An 8 mL, stainless steel Harvard Apparatus syringe was filled with the solution and then attached to the flow apparatus according to setup A. The flow apparatus itself was set up at a flow rate = 22.3 µL/min and flow rate of CO_2 was adjusted to create 1:1 liquid/gas plugs. After filling the reactor and 2 mins to equilibrate, the valves were closed and the tubing reactor (stainless steel, 1/16", I.D. 0.03", 762 cm, volume = 3.47 mL) was placed into the oil bath at 120 °C for 30 mins. Then the solution in the SFMT reactor was washed out by diethyl ether and quenched with distilled water, extracted twice with diethyl ether and washed with water. The organic phase was combined and the solvent removed under reduced pressure with the temperature set below 20 °C to avoid loss of internal standard mesitylene, indicated the desired product in 48% yield according to crude ^{1}H NMR analysis.

[4+2] Singlet Oxygen Cycloaddition[3]

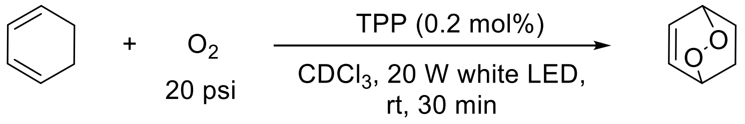

Batch Reaction

To a round-bottomed flask was added a solution of 1,3-cyclohexadiene (83.3 mg, 1.4 mmol), tetraphenylporphyrin (TPP) (2 mg, 2.8x10^{-3} mmol), and 1,3,5-trimethoxybenzene (79 mg, 0.47 mmol) in $CDCl_3$ (5.0 mL). The reaction solution was bubbled with oxygen for 5 mins while protected from light. An oxygen balloon was attached to the flask and the reaction mixture was stirred at rt with a distance of 3-5 cm from a 20 W white LED lamp. After 30 mins, the reaction was analyzed via crude ^{1}H NMR which indicated the desired product was obtained in 49% yield.

Continuous-Flow Reaction

The O_2 gas cylinder is pressurized to ~20 psi higher than the desired back-pressure (20 psi) of the system. A 10 mL volumetric flask was charged with a solution of 1,3-cyclohexadiene (83.3 mg, 1.4 mmol), tetraphenylporphyrin (TPP) (2 mg, 2.8x10^{-3} mmol), and 1,3,5-trimethoxybenzene (79 mg, 0.47 mmol) in $CDCl_3$ (5.0 mL). An 8 mL, stainless steel Harvard Apparatus syringe was filled with the solution and then attached to the flow

apparatus according to setup A. The tubing reactor (HPFA, 1/16”, I.D. 0.03”, 105 cm, volume = 0.48 mL) was placed at a distance of 3-5 cm to a 20 W white LED lamp. The flow apparatus itself was set up at t_R = 30 mins, flow rate = 7.98 µL/min and flow rate of O_2 was adjusted to create a 1:1 liquid/gas plugs. After approximately 1.5 hours to equilibrate, the solution was collected for 30 mins, and analyzed via crude ^{1}H NMR analysis, which indicated the desired product was obtained in 65% yield.

Stop-Flow Reaction

The O_2 gas cylinder is pressurized to ~20 psi higher than the desired back-pressure of the system (20 psi). A 10 mL volumetric flask was charged with a solution of 1,3-cyclohexadiene (83.3 mg, 1.4 mmol), tetraphenylporphyrin (TPP) (2 mg, 2.8x10^{-3} mmol), and 1,3,5-trimethoxybenzene (79 mg, 0.47 mmol) in $CDCl_3$ (5.0 mL). An 8 mL, stainless steel Harvard Apparatus syringe was filled with the solution and then attached to the flow apparatus according to setup A. The flow apparatus itself was set up at a flow rate = 7.98 µL/min and flow rate of O_2 was adjusted to create 1:1 liquid/gas plugs. After filling the tubing followed by approximately 2 mins equilibration, valves were closed and the tubing reactor (HPFA, 1/16”, I.D. 0.03”, 105 cm, volume = 0.48 mL) was placed at a distance of 3-5 cm to a 20 W white LED lamp for 30 mins. Then the crude mixture was analyzed via crude ^{1}H NMR, which indicated the desired product was obtained in 62% yield.

Diels Alder Reaction[4]

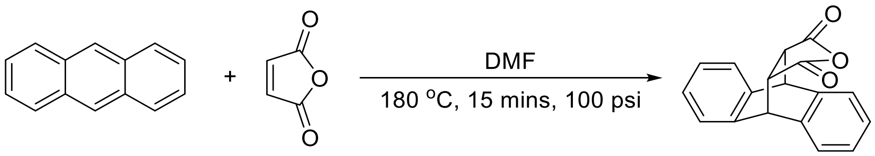

Batch Reaction

Under N_2 atmosphere, a seal tube reactor was charged with a solution of anthracene (0.8 g, 4.48 mmol), maleic anhydride (0.4 g, 4.08 mmol) and mesitylene (163 mg, 1.16 mmol) in DMF (10.0 mL) was pressurized to 100 psi with N_2 and the reaction was heated at 180 °C for 15 min. Then reaction mixture was cooled in an ice bath and the pressure was released slowly. The reaction mixture was poured into water and extracted twice with diethyl ether, and washed with water. The organic phase was combined and concentrated in vacuo with the temperature below 20 °C. The crude residue was analyzed via ^{1}H NMR, which indicated an 11% yield of the Diels-Alder product.

Continuous-Flow Reaction

A 10 mL volumetric flask was charged with a solution of anthracene (0.8 g, 4.48 mmol), maleic anhydride (0.4 g, 4.08 mmol) and mesitylene (163 mg, 1.16 mmol) in DMF (10.0 mL). An 8 mL, stainless steel Harvard Apparatus syringe was filled with the solution and then attached to the flow apparatus according to setup C with BPR (100 psi). The tubing reactor (stainless steel, 1/16”, I.D. 0.03”, 762 cm, volume = 3.47 mL) was placed into an oil bath at 180 °C. The flow apparatus itself was set up as t_R = 15 mins, flow rate = 227.1 µL/min. After approximately 45 mins equilibration, the solution was collected for 30 mins, and poured into water, extracted twice with ether, and washed with water. The

organic phase was combined and concentrated in vacuo. The crude residue was analyzed via ^{1}H NMR, indicated a 56% yield of the Diels-Alder product.

Stop-Flow Reaction

A 10 mL volumetric flask was charged with a solution of anthracene (0.8 g, 4.48 mmol), maleic anhydride (0.4 g, 4.08 mmol) and mesitylene (163 mg, 1.16 mmol) in DMF (10.0 mL). An 8 mL, stainless steel Harvard Apparatus syringe was filled with the solution and then attached to the flow apparatus according to setup C with BPR (100 psi). The tubing reactor was filled and after approximately 2 mins equilibration, the valves were closed, and the tubing reactor (stainless steel, 1/16", I.D. 0.03", 762 cm, volume = 3.47 mL) was placed into oil bath at 180 ^{o}C for 15 mins. Then the solution in SFMT was washed into water, extracted twice with ether, and washed with water. The organic phase was combined and concentrated under vacuum. The crude residue was analyzed via ^{1}H NMR, indicated a 53% yield of the Diels-Alder product.

Jones Oxidation Reaction[5]

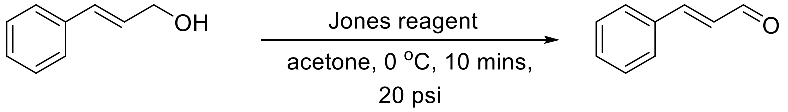

Batch Reaction

A 50 mL round-bottomed flask was charged with cinnamyl alcohol (250 mg 1.86 mmol) in acetone (5.0 mL) and cooled to 0 ^{o}C. A solution of Jones reagent (1.0 mL) in acetone (9.0 mL) was added in one portion. After stirring at 0°C for 10 mins, isopropyl alcohol was added dropwise to quench excess Jones reagent until the color of solution turned to deep green. The solvent was concentrated under reduced pressure, and the crude mixture was dissolved in distilled water and extracted twice with diethyl ether. The combined organic phase was washed with water, dried over anhydrous magnesium sulfate, and concentrated under vacuum. The product was purified by column chromatography to afford 172 mg cinnamaldehyde (70% yield).

Continuous-Flow Reaction

An 8 mL stainless steel syringe is filled with a solution of cinnamyl alcohol (250 mg 1.86 mmol) in acetone (5.0 mL). A separate 8 mL stainless steel syringe was filled with a solution of Jones reagent (1.0 mL) in acetone (9.0 mL). The two syringes were attached to the flow apparatus (two syringe pump) according to setup B with BPR (20 psi). The tubing reactor (HPFA, 1/16", I.D. 0.03", 400 cm, volume = 1.82 mL) was placed in an ice bath at 0 ^{o}C. The flow apparatus itself was set up as t_R = 10 mins, flow rate (pump 1, cinnamyl alcohol) at 60.8 µL/min and flow rate (pump 2, Jones reagent) at 121.6 µL/min. After approximately 20 mins to equilibrate, the solution was collected for 10 mins and quenched by isopropyl alcohol. After the solvent was concentrated under reduced pressure, the crude mixture was dissolved in distilled water and extracted twice with diethyl ether. The combined organic phase was washed with water, dried over anhydrous magnesium sulfate, and concentrated under vacuum. The product was purified by column chromatography to afford 22 mg cinnamaldehyde (76% yield).

Stop Flow Reaction

An 8 mL stainless steel syringe is filled with a solution of cinnamyl alcohol (250 mg 1.86 mmol) in acetone (5.0 mL). A separate 8 mL stainless steel syringe was filled with a solution of Jones reagent (1.0 mL, 10 mmol) in acetone (9.0 mL). The two syringes were attached to the flow apparatus (two syringe pump) according to setup B with BPR (20 psi). The tubing reactor (HPFA, 1/16", I.D. 0.03", 400 cm, volume = 1.82 mL) was placed into ice bath at 0 °C. The flow apparatus itself was set up as flow rate (pump 1, cinnamyl alcohol) at 60.8 µL/min and flow rate (pump 2, Jones reagent) at 121.6 µL/min. After the reactor was fully filled and approximate 1 min equilibration, the valves were closed. The tubing reactor (HPFA, 1/16", I.D. 0.03", 400 cm) was kept in an ice bath for 10 mins, before the solution in SFMT was quenched by isopropyl alcohol. After the solvent was concentrated under reduced pressure, the crude mixture was dissolved in distilled water and extracted twice with diethyl ether. The combined organic phase was washed with water, dried over anhydrous magnesium sulfate, and concentrated under vacuum. The product was purified by column chromatography to afford 23 mg cinnamaldehyde (77% yield).

Biginelli Reaction[6]

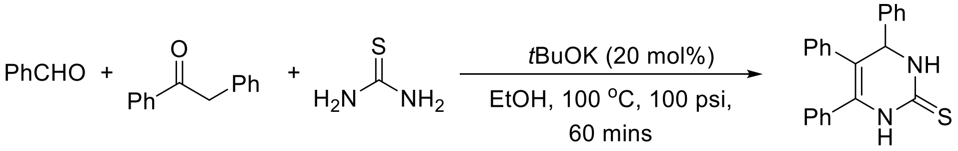

Batch Reaction

Under Ar atmosphere, a Parr reactor was charged with a solution of benzaldehyde (106 mg, 1 mmol), 2-phenylacetophenone (196 mg, 1.1 mmol), thiourea (152 mg, 2 mmol), and 1,3,5-trimethoxybenzene (55.5 mg, 0.33 mmol) in ethanol (5.0 mL). *t*BuOK (22 mg, 0.2 mmol) was added, and the reactor was pressurized to 100 psi with Ar and stirred at 100 °C for 60 min, then cooled in an ice bath and the pressure was released slowly. The resulting reaction mixture was collected into a vial and the solvent was removed under reduced pressure. The crude mixture was analyzed by ^{1}H NMR and indicated a 67% yield of the desired product.

Continuous-Flow Reaction

A 25 mL vial was charged with a solution of benzaldehyde (106 mg, 1 mmol), 2-phenylacetophenone (196 mg, 1.1 mmol), thiourea (152 mg, 2.0 mmol), and 1,3,5-trimethoxybenzene (55.5 mg, 0.33 mmol) in ethanol (5.0 mL). ^{t}BuOK (22 mg, 0.2 mmol) was added, then the vial was bubbled with Ar for 5 mins. An 8 mL, stainless steel Harvard Apparatus syringe was filled with the solution and then attached to the flow apparatus according to setup C with BPR (100 psi). The tubing reactor (HPFA, 1/16", I.D. 0.03", 400 cm, volume = 1.82 mL) was placed in an oil bath at 100 °C. The flow apparatus itself was set up at t_R = 60 mins, flow rate at 30.4 µL/min. After approximate 60 mins equilibration, the solution was collected for 60 mins. The solvent was removed

under reduced pressure. The crude mixture was analyzed by ^{1}H NMR to indicate a 49% yield of the desired product.

Stop-Flow Reaction

A 25 mL vial was charged with a solution of benzaldehyde (106 mg, 1 mmol), 2-phenylacetophenone (196 mg, 1.1 mmol), thiourea (152 mg, 2.0 mmol), and 1,3,5-trimethoxybenzene (55.5 mg, 0.33 mmol) in ethanol (5.0 mL). ^{t}BuOK (22 mg, 0.2 mmol) was added, and the vial was bubbled with Ar for 5 mins. An 8 mL stainless steel Harvard Apparatus syringe was filled with the solution and then attached to the flow apparatus according to setup C with BPR (100 psi). The flow apparatus itself was set up at flow rate 30.4 µL/min. After approximate 5 mins equilibration, the valves were closed, and the tubing reactor (HPFA, 1/16", I.D. 0.03", 400 cm, volume = 1.82 mL) was placed in an oil bath at 100 ^{o}C for 60 mins. Then the reaction mixture was cooled to rt and the solvent was removed under reduced pressure. The crude mixture was analyzed by ^{1}H NMR which indicated a 70% yield of the desired product.

Trans-isomerization Reaction[7]

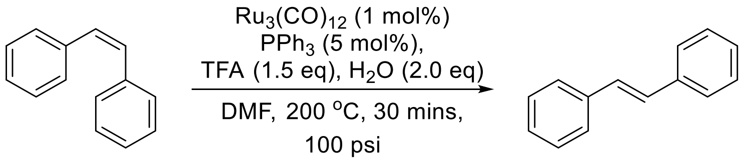

Batch Reaction

Under Ar atmosphere, a seal tube ractor was charged with a solution of $Ru_3(CO)_{12}$ (16 mg, 0.025 mmol), cis-stilbene (0.45 mL, 2.5 mmol), triphenylphosphine (33 mg, 0.125 mmol), trifluoroacetic acid (0.29 mL, 3.75 mmol), H_2O (90 mg, 5.0 mmol), 1,3,5-trimethoxybenzene (140 mg, 0.83 mmol) in DMF (5.0 mL), which was pressurized to 100 psi with Ar and stirred at 200 ^{o}C for 30 min. Then the reactor was cooled in an ice bath and the pressure was released slowly. The solution was poured into water and extracted twice with ether. The combined organic phase was concentrated in vacuo and analyzed by crude ^{1}H NMR, indicated a 72% yield of the desired product.

Continuous-Flow Reaction

A 10 mL vial was charged with a solution of $Ru_3(CO)_{12}$ (16 mg, 0.025 mmol), cis-stilbene (0.45 mL, 2.5 mmol), triphenylphosphine (33 mg, 0.125 mmol), trifluoroacetic acid (0.29 mL, 3.75 mmol), H_2O (90 mg, 5.0 mmol), 1,3,5-trimethoxybenzene (140 mg, 0.83 mmol) in DMF (5.0 mL). An 8 mL, stainless steel Harvard Apparatus syringe was filled with the solution and then attached to the flow apparatus according to setup C with BPR (100 psi). The tubing reactor (stainless steel, 1/16", I.D. 0.03", 150 cm, volume = 0.68 mL) was placed into oil bath at 200 ^{o}C. The flow apparatus itself was set up at t_R = 30 mins, flow rate = 22.8 µL/min. After approximate 90 mins equilibration, the solution was collected for 30 mins and poured into water and extracted twice with ether. The combined organic phase was concentrated in vacuo and analyzed by crude ^{1}H NMR, indicated a 73% yield of the desired product.

Stop-Flow Reaction

A 10 mL vial was charged with a solution of $Ru_3(CO)_{12}$ (16 mg, 0.025 mmol), cis-stilbene (0.45 mL, 2.5 mmol), triphenylphosphine (33 mg, 0.125 mmol), trifluoroacetic acid (0.29 mL, 3.75 mmol), H_2O (90 mg, 5.0 mmol), 1,3,5-trimethoxybenzene (140 mg, 0.83 mmol) in DMF (5.0 mL). An 8 mL, stainless steel Harvard Apparatus syringe was filled with the solution and then attached to the flow apparatus according to setup C with BPR (100 psi). The flow apparatus itself was set up at a flow rate = 22.8 µL/min. After approximate 2 mins equilibration, the valves were closed and the tubing reactor (stainless steel, 1/16", I.D. 0.03", 150 cm, volume = 0.68 mL) was placed in an oil bath at 200 ^{o}C for 30 mins. Then the solution in SFMT reactor was washed out with diethyl ether into water and extracted twice with ether. The combined organic phase was concentrated in vacuo and analyzed by crude ^{1}H NMR, indicated a 62% yield of the desired product.

Olefin Metathesis[8]

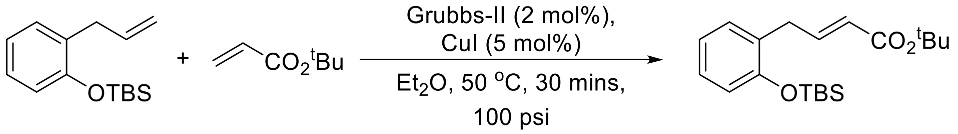

Batch Reaction

Under Ar atmosphere, a Parr reactor charged with a solution of tert-butyl(2-allylphenoxy)dimethylsilane (124 mg, 0.50 mmol), tert-butyl acrylate (192 mg, 1.50 mmol), Grubbs-II catalyst (8.5 mg, 10.0 µmol), CuI (2.9 mg, 15.0 µmol), mesitylene (20.4 mg, 0.17 mmol) in diethyl ether (5.0 mL) was pressurized to 100 psi with Ar and stirred at 50 ^{o}C for 30 mins. After cooling in an ice bath, water was added and the mixture was extracted twice with ether. The organic phase was combined and concentrated under reduced pressure, indicated the desired product in 60% yield according to crude ^{1}H NMR analysis.

Continuous-Flow Reaction

A 10 mL vial was charged with a solution of tert-butyl(2-allylphenoxy)dimethylsilane (124 mg, 0.50 mmol), tert-butyl acrylate (192 mg, 1.50 mmol), Grubbs-II catalyst (8.5 mg, 10.0 µmol), CuI (2.9 mg, 15.0 µmol), mesitylene (20.4 mg, 0.17 mmol) in diethyl ether (5.0 mL). An 8 mL, stainless steel Harvard Apparatus syringe was filled with the solution and then attached to the flow apparatus according to setup C with BPR (100 psi). The tubing reactor (HPFA, 1/16", I.D. 0.03", 105 cm, volume = 0.48 mL) was placed in an oil bath at 50 ^{o}C. The flow apparatus itself was set up with t_R = 30 mins, flow rate = 15.96 µL/min. After approximate 90 mins equilibration, the flow was collected for 30 mins. Then the product was poured into water and extracted twice with ether. The organic phase was combined and concentrated under reduced pressure. The crude ^{1}H NMR analysis indicated the desired product in a 73% yield.

Stop-Flow Reaction

A 10 mL vial was charged with a solution of tert-butyl(2-allylphenoxy)dimethylsilane (124 mg, 0.50 mmol), tert-butyl acrylate (192 mg, 1.50 mmol), Grubbs-II catalyst (8.5 mg, 10.0 μmol), CuI (2.9 mg, 15.0 μmol), mesitylene (20.4 mg, 0.17 mmol) in diethyl ether (5.0 mL). An 8 mL, stainless steel Harvard Apparatus syringe was filled with the solution and then attached to the flow apparatus according to setup C with BPR (100 psi). The microtubing was filled and after approximate 2 mins equilibration, the valves were closed. The tubing reactor (HPFA, 1/16", I.D. 0.03", 105 cm, volume = 0.48 mL) was placed in an oil bath at 50 °C for 30 mins. Then the solution in SFMT was poured into water and extracted twice with ether. The organic phase was combined and concentrated under reduced pressure, indicating that the desired product was obtained in 68% yield according to crude ^{1}H NMR analysis.

6. Sonogashira Coupling with Acetylene

6.1 Reaction optimization

Reaction optimization was conducted with the SFMT system.

Table S2. Reaction optimization of Sonogashira coupling with acetylene (with selected data).

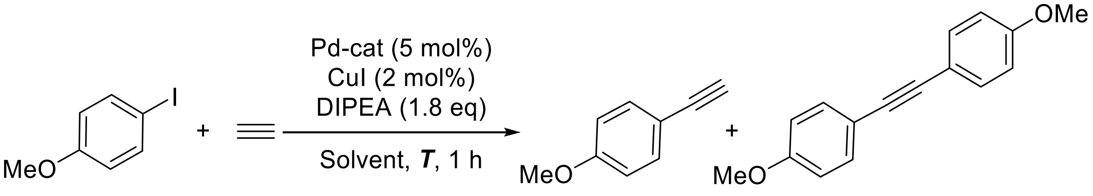

1a **2a** **3a**

Entry[a]	Pd-cat	Solvent	*T* [°C]	Yield of **2a** [%][b]	Yield of **3a** [%][b]
1	$Pd(PPh_3)_2Cl_2$	DMSO	100	73	3
2	$Pd(PPh_3)_2Cl_2$	DMF	100	20	<1
3	$Pd(PPh_3)_2Cl_2$	NMP	100	<1	<1
4[c]	$Pd(PPh_3)_2Cl_2$	DMSO	100	24	<1
5	$Pd(PPh_3)_4$	DMSO	100	73	3
6	$Pd(dppf)Cl_2$	DMSO	100	56	2
7[d]	$Pd(dba)_2/PPh_3$	DMSO	100	54	3
8	$Pd(PPh_3)_2Cl_2$	DMSO	60	80	4
9	$Pd(PPh_3)_2Cl_2$	DMSO	40	87	2
10	$Pd(PPh_3)_2Cl_2$	DMSO	rt	78	3
**11**[e]	**$Pd(PPh_3)_2Cl_2$**	**DMSO**	**rt**	**96**	**4**

[a] Reactions were carried out with **1a** (0.1 M). [b] Yields were determined by GC analysis with 1,3,5-trimthoxybenzene as the internal standard. [c] The reaction was carried out with 1 mol% $Pd(PPh_3)_2Cl_2$. [d] The reaction was carried out with 5 mol% $Pd(dba)_2$ and 10 mol% PPh_3. [e] The reaction was conducted for 2 h reaction time before quenching.

6.2 General procedure for batch, SFMT, and continuous-flow reactions

General procedure A (batch reaction)

Under N_2 atomsphere, a solution of aryl iodide (1.0 mmol), $Pd(PPh_3)_2Cl_2$ (35.1 mg, 0.05 mmol, 5 mol%), copper(I) iodide (3.8 mg, 0.02 mmol, 2 mol%), DIPEA (310 µL, 1.8 mmol, 1.8 equiv) and 1,3,5-trimethoxybenzene (84.0 mg, 0.5 mmol, 0.5 eq) in DMSO (10.0 mL) was bubbled with acetylene carefully for 5 min and stirred at r.t for 2 h. Then the mixture was quenched by 10 mL saturated NH_4Cl aqueous solution, extracted by diethyl ether (5 mL x 2), and the organic phase was combined to check the yield by GC analysis of the crude mixture (1,3,5-trimethoxybenzene as the internal standard).

General procedure B (using SFMT reactors)

The acetylene tank is pressurized to ~20 psi higher than the desired back-pressure (5 psi) of the system. A flame dried 10 mL round bottom flask was equipped with a rubber septum and magnetic stir bar and was charged with aryl iodide (0.25 mmol), $Pd(PPh_3)_2Cl_2$ (8.5 mg, 0.0125 mmol, 5 mol%), copper(I) iodide (1.0 mg, 0.005 mmol, 2 mol%), DIPEA (80 µL, 0.45 mmol, 1.8 eq) and 1,3,5-trimethoxybenzene (21.0 mg, 0.125 mmol, 0.5 eq) in DMSO (2.5 mL). The mixture was bubbled with argon for 15 min. A stainless steel syringe was filled with the solution and then attached to the flow apparatus (syringe pump). The stop-flow micro tubing reactor was made of HPFA tubing (O.D. 1/16", I.D. 0.03", 300 cm, volume = 1.37 mL). The flow apparatus itself was set up at a flow rate = 300 µL/min, and flow of acetylene was adjusted to create ~1:1 liquid/gas plugs. After approximately 2 min equilibration, the valves were closed, and the tubing was held at r.t or in the oil bath at 60 ^{o}C, and kept for 2 h. Then the mixture was quenched with saturated NH_4Cl aqueous solution (4.0 mL), extracted with DMSO (0.5 mL) and diethyl ether (1.5 mL) in sequence. After separation, the organic phase was collected for GC analysis to check the yield (1,3,5-trimethoxybenzene as the internal standard).

General procedure C (continuous-flow reactions)

The acetylene tank is pressurized to ~30 psi. A flame dried 10 mL round bottom flask was equipped with a rubber septum and a magnetic stir bar and was charged with aryl iodide (1.0 mmol), $Pd(PPh_3)_2Cl_2$ (35.1 mg, 0.05 mmol, 5 mol%), copper(I) iodide (3.8 mg, 0.02 mmol, 2 mol%), DIPEA (310 µL, 1.8 mmol, 1.8 eq) and 1,3,5-

trimethoxybenzene (84 mg, 0.5 mmol, 0.5 eq) in DMSO (10.0 mL). The mixture was vacuum degassed before adding DIPEA and argon protection. An Asia pump was applied to pump the reaction mixture. The tubing reactor (HPFA, O.D. 1/16", I.D. 0.03", 600 cm, volume = 2.74 mL) was placed at r.t. The flow apparatus itself was set up at a flow rate = 12 µL/min, t_R = 2 h and flow of acetylene was adjusted to create 1:1 liquid/gas plugs. After approximately 4 h for equilibration, the solution was collected for 30 mins, and quenched with saturated NH_4Cl aqueous solution (4.0 mL) and diethyl ether (1.5 mL). After separation, the organic phase was collected for GC analysis of the crude mixture to check the yield (1,3,5-trimethoxybenzene as the internal standard).

6.3 Large-scale continuous production

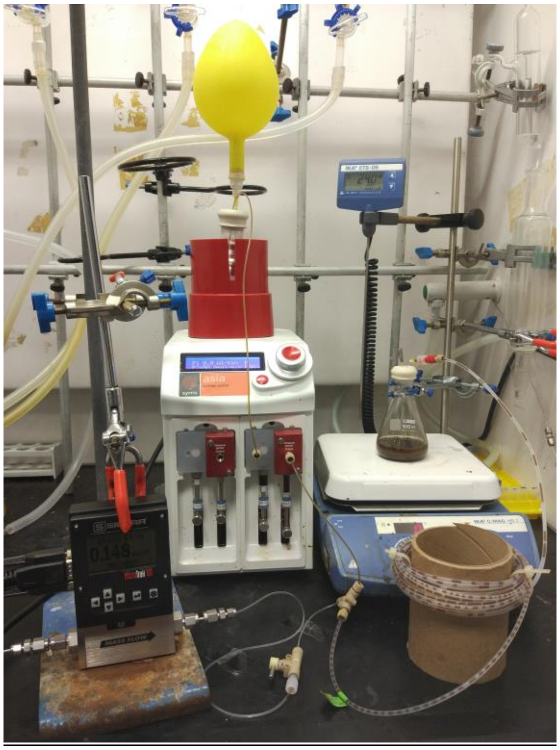

Figure S16. Continuous-flow setup for Sonogashira coupling with acetylene.

General Procedure:

The acetylene tank is pressurized to ~30 psi. A flame dried 200 mL round bottom flask was equipped with a rubber septum and magnetic stir bar and was charged with 4-iodoanisole (2.32 g, 10 mmol), $Pd(PPh_3)_2Cl_2$ (351 mg, 0.5 mmol, 5 mol%), copper(I) iodide (38 mg, 0.2 mmol, 2 mol%), DIPEA (3.1 mL, 18 mmol, 1.8 equiv) and 1,3,5-

trimethoxybenzene (840 mg, 5 mmol, 0.5 equiv) in DMSO (100 mL). The mixture was vacuum degassed before DIPEA was added and protected under argon. As shown in Figure S16, an Asia pump was filled with the starting material solution and then attached to the flow system. The tubing reactor (HPFA, O.D. 1/8”, I.D. 0.062”, 900 cm, volume = 17.53 mL) was placed at r.t. The flow apparatus itself was set up as flow rate = 50 μL/min, t_R approximate 2 h and flow of acetylene was controlled by mass flow controller to adjust to create 1:1 liquid/gas plugs. After approximately 4 h for equilibration, the solution was collected for 25 h, and quenched with saturated NH_4Cl aqueous solution (300 mL) and extrated with diethyl ether (100 mL x 4). After separation, the organic phase was collected to check GC yields, which indicated **2a** of 97% yield, and **3a** of 3% yield (1,3,5-trimethoxybenzene as the internal standard). The combined organic layers were dried with Na_2SO_4. After filtration and evaporation under vacuum at 0 °C, the crude residue was purified by flash chromatography (eluent: pentane), which afforded 797 mg products, in 81% yield.

6.4 One-pot Sonogashira coupling of bromoanisole with acetylene

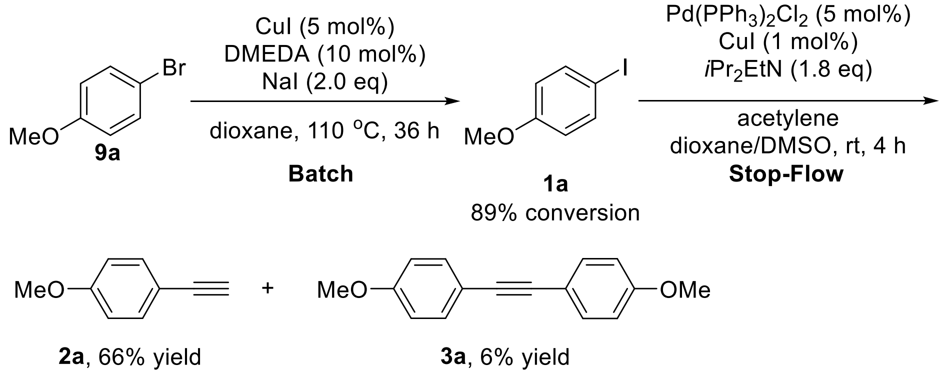

General Procedure[9]

Under N_2 atompshere, in a sealed tube, a solution of 4-bromoanisole **9a** (561 mg, 3.0 mmol, 1.0 eq), CuI (28.5 mg, 0.15 mmol, 5 mo%), *N*,*N*’-dimethylethylenediamine (DMEDA) (26.4 mg, 0.3 mmol, 10 mol%), NaI (899 mg, 0.6 mmol, 2.0 eq), 1,3,5-trimethoxybenzene (252 mg, 1.5 mmol, 0.5 eq) in dry dioxane (3.0 mL) was stirred at 110 °C for 36 h. After cooling to rt, taking the supernatant for analyzing and using for the

next step directly. The ^{1}H NMR analysis of the crude mixture indicated an 89% conversion.

In a flame dried 10 mL round bottom flask, the supernatant solution (0.8 mL, containing 4-iodoanisole (129 mg, 0.55 mmol, 1.0 eq) and 1,3,5-trimethoxybenzene (70 mg, 0.41 mmol, 0.75 eq) was added to a solution of $Pd(PPh_3)_2Cl_2$ (19.3 mg, 0.0275 mmol, 5 mol%), CuI (1.1 mg, 0.0055 mmol, 1 mol%) and DIPEA (176 µL, 0.99 mmol, 1.8 eq) in DMSO (2.0 mL). The mixture was bubbled with argon for 15 min. The acetylene tank was pressurized to ~20 psi higher than the desired back-pressure (5 psi) of the system. A stainless steel syringe was filled with the solution and then attached to the SFMT system. The stop-flow micro tubing reactor was made of HPFA tubing (O.D. 1/16", I.D. 0.03", 300 cm, volume = 1.37 mL). The flow apparatus itself was set up with a flow rate = 300 µL/min, and flow of acetylene was adjusted to create ~1:1 liquid/gas plugs. After approximately 2 min equilibration, the valves were closed, and the tubing was held at r.t, and kept for 4 h. Then the reaction mixture was washed out with DMSO (0.5 mL) and diethyl ether (1.5 mL) and quenched with saturated NH_4Cl aqueous solution (4.0 mL). After separation, the organic phase was collected for GC analysis, which indicated **2a** of 66% yield and **3a** of 6% yield (1,3,5-trimethoxybenzene as the internal standard).

6.5 New compound characterization

General method of GC yield calculation

According to GC spectrum of reaction product, we used calibration curve to calculate the GC yield. From the calibration curve, we derived a linear regression equation,

$$y = ax + b.$$

In this equation,

$$y = \frac{(integration\ of\ the\ product)}{(integration\ of\ internal\ standard)}$$

$$x = \ mass\ of\ the\ product\ (mg)$$

Here is an example of calculation yield of main product **2a** and side product **3a**. From Figure S19, we know that $y_{\mathbf{2a}}$ = 100%/98.25% =1.018, $y_{\mathbf{3a}}$ = 19.57%/98.25%=0.199. So $x_{\mathbf{2a}}$ = 31.78 mg and $x_{\mathbf{3a}}$ = 1.19 mg.

Therefore, the $yield_{\mathbf{2a}}$=31.78/33.04=96% and $yield_{\mathbf{3a}}$=1.19/29.79=4%.

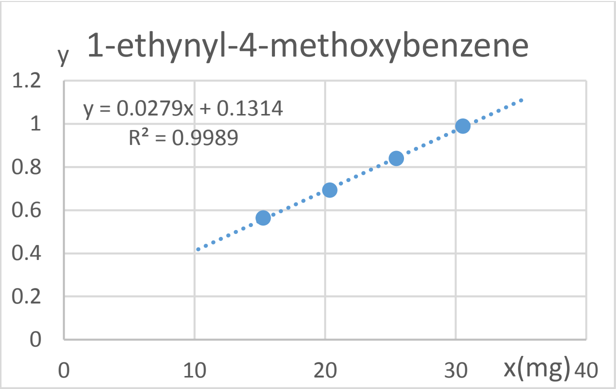

Figure S17. Calibration curve of main product

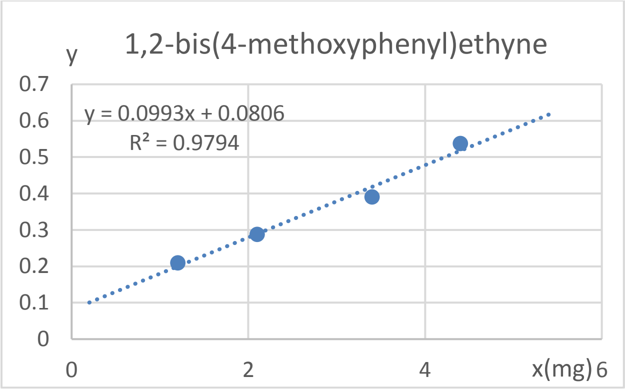

Figure S18. Calibration curve of the byproduct

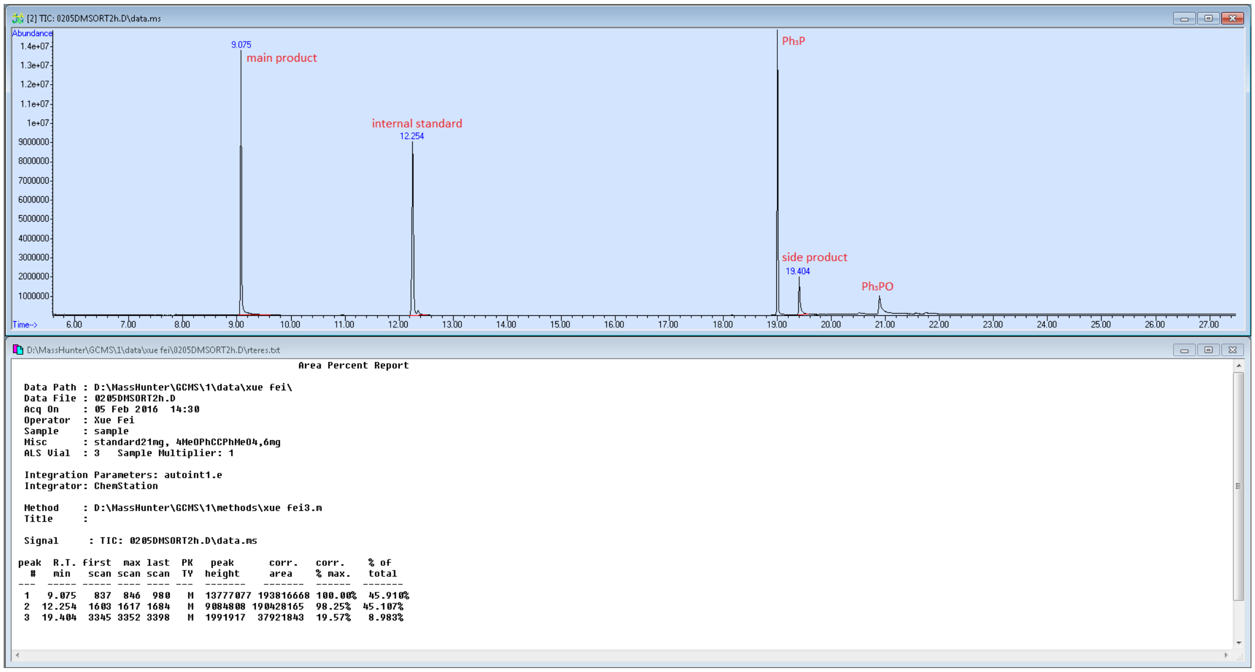

Figure S19. GC Spectrum

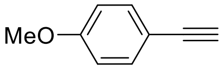**1-ethynyl-4-methoxybenzene (2a)**

Batch: Followed the general procedure **A** in batch reaction with 4-iodoanisole (234.0 mg, 1.00 mmol) at rt. The GC analysis of the crude mixture indicated **2a** of 45% yield with **3a** 14% yield.

Stop-flow: Followed the general procedure **B** in SFMT reactors with 4-iodoanisole (58.5 mg, 0.25 mmol) at rt. The GC analysis of the crude mixture indicated **2a** of 96% yield together with 4% of **3a**. ^{1}H NMR (400 MHz, $CDCl_3$) δ 7.44 (d, *J* = 9.2 Hz, 2H), 6.85 (d, *J* = 9.2 Hz, 2H), 3.80 (s, 3H), 3.01 (s, 1H).

Characterization of compound **2a** has been reported in previous literature.[10]

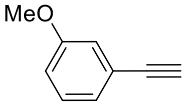**1-ethynyl-3-methoxybenzene (2b)**

Followed the general procedure **B** in SFMT with 3-iodoanisole (58.5 mg, 0.25 mmol) at rt. The GC analysis of the crude mixture indicated **2b** of 97% yield together with **3b** of 2% yield.

Characterization of compound **2b** has been reported in previous literature. [11]

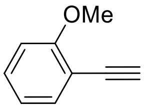**1-ethynyl-2-methoxybenzene (2c)**

Followed the general procedure **B** in SFMT with 2-iodoanisole (58.5 mg, 0.25 mmol) at 60 ^{o}C. The GC analysis of the crude mixture indicated **2c** of 47% yield together with **3c** of 2% yield.

Characterization of compound **2c** has been reported in previous literature. [12]

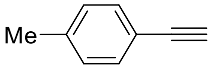**1-ethynyl-4-methylbenzene (2d)**

Followed the general procedure **B** in SFMT with 1-iodo-4-methylbenzene (57.0 mg, 0.25 mmol) at rt. The GC analysis of the crude mixture indicated **2d** of 75% yield together with **3d** of 3% yield.

Characterization of compound **2d** has been reported in previous literature. [10]

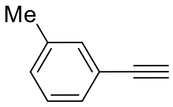

1-ethynyl-3-methylbenzene (2e)

Followed the general procedure **B** in SFMT with 1-iodo-3-methylbenzene (57.0 mg, 0.25 mmol) at rt. The GC analysis of the crude mixture indicated **2e** of 86% yield together with **3e** of 5% yield.

Characterization of compound **2e** has been reported in previous literature. [13]

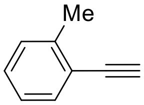

1-ethynyl-2-methylbenzene (2f)

Followed the general procedure **B** in SFMT with 1-iodo-2-methylbenzene (57.0 mg, 0.25 mmol) at 60 ^{o}C. The GC analysis of the crude mixture indicated **2f** of 48% yield together with **3f** of 2% yield.

Characterization of compound **2f** has been reported in previous literature. [13]

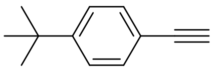

1-(tert-butyl)-4-ethynylbenzene (2g)

Followed the general procedure **B** in SFMT with 1-(tert-butyl)-4-iodobenzene (65.0 mg, 0.25 mmol) at rt. The GC analysis of the crude mixture indicated **2g** of 80% yield together with **3g** of 6% yield.

Characterization of compound **2g** has been reported in previous literature. [14]

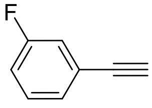

1-ethynyl-3-fluorobenzene (2h)

Followed the general procedure **B** in SFMT with 1-fluoro-3-iodobenzene (55.5 mg, 0.25 mmol) at rt. The GC analysis of the crude mixture indicated **2h** of 68% yield together with **3h** of 3% yield.

Characterization of compound **2h** has been reported in previous literature. [15]

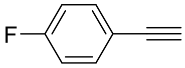 **1-ethynyl-4-fluorobenzene (2i)**

Stop-flow: Followed the general procedure **B** in SFMT with 1-fluoro-4-iodobenzene (55.5 mg, 0.25 mmol) at rt. The GC analysis of the crude mixture indicated **2i** of 70% yield together with **3i** of 3% yield.

Continuous-flow: Followed the general procedure **C** in continuous-flow with 1,-fluoro-4-iodobenzene (166.5 mg, 0.75 mmol). GC analysis of the crude mixture indicated **2i** of 62% yield together with **3i** of 5% yield.

Characterization of compound **2i** has been reported in previous literature. [13]

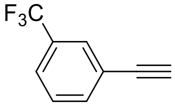 **1-ethynyl-3-(trifluoromethyl)benzene (2j)**

Followed the general procedure **B** in SFMT with 1-iodo-3-(trifluoromethyl)benzene (68.0 mg, 0.25 mmol) at rt. The GC analysis of the crude mixture indicated **2j** of 67% yield together with **3j** of 5% yield.

Characterization of compound **2j** has been reported in previous literature. [15]

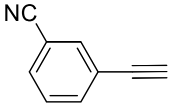 **3-ethynylbenzonitrile (2k)**

Followed the general procedure **B** in SFMT with 14-iodobenzonitrile (57.3 mg, 0.25 mmol) at rt. The GC analysis of the crude mixture indicated **2k** of 80% yield together with **3k** of 10% yield.

Characterization of compound **2k** has been reported in previous literature. [16]

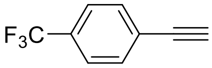 **1-ethynyl-4-(trifluoromethyl)benzene (2l)**

Stop-flow: Followed the general procedure **B** in SFMT with 1-iodo-4-(trifluoromethyl)benzene (68.0 mg, 0.25 mmol) at rt. The GC analysis of the crude mixture indicated **2l** of 70% yield together with **3l** of 6% yield.

Continuous-flow: Followed the general procedure **C** in continuous-flow with 1-iodo-4-(trifluoromethyl)benzene (204.0 mg, 0.75 mmol). GC analysis of the crude mixture indicated **2l** of 85% yield together with **3l** of 5% yield.

Characterization of compound **2l** has been reported in previous literature.[15]

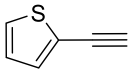

2-ethynylthiophene (2m)

Followed the general procedure **B** in SFMT with 2-iodothiophene (52.0 mg, 0.25 mmol) at rt. The GC analysis of the crude mixture indicated **2m** of 65% yield together with **3m** of 3% yield.

Characterization of compound **2m** has been reported in previous literature.[17]

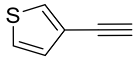

3-ethynylthiophene (2n)

Followed the general procedure **B** in SFMT with 3-iodothiophene (52.0 mg, 0.25 mmol) at rt. The GC analysis of the crude mixture indicated **2n** of 83% yield together with **3n** of 2% yield.

Characterization of compound **2n** has been reported in previous literature.[12]

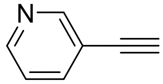

3-ethynylpyridine (2o)

Stop-flow: Followed the general procedure **B** in SFMT with 3-iodopyridine (51.3 mg, 0.25 mmol) at rt. The GC analysis of the crude mixture indicated **2o** of 86% yield together with **3o** of 6% yield.

Continuous-flow: Followed the general procedure **C** in continuous-flow with 3-iodopyridine (153.8 mg, 0.75 mmol). GC analysis of the crude mixture indicated **2o** of 90% yield together with **3o** of 4% yield.

Characterization of compound **2o** has been reported in previous literature.[10]

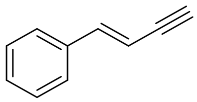

(*E*)-but-1-en-3-yn-1-ylbenzene (2p)

Followed the general procedure **B** in SFMT with trans-β-bromostyrene (45.8 mg, 0.25 mmol), $Pd(PPh_3)_2Cl_2$ (17 mg, 0.025 mmol, 10 mol%) at rt for 4 h. The GC analysis of the crude mixture indicated **2p** of 79% yield together with **3p** of 2% yield.
Characterization of compound **2p** has been reported in previous literature. [18]

7. Fulvene Synthesis

7.1 Reaction optimization

Reaction optimization was carried out with the SFMT platform.

Table S3. Reaction optimization of fulvene synthesis (with selected data).

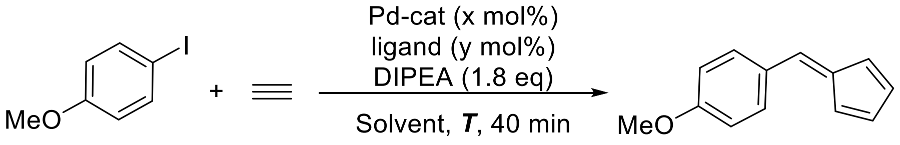

Entry [a]	Catalyst (x mol%)	Ligand (y mol%)	Solvent	T [°C]	Yield [%][b]
1	$Pd(PPh_3)_2Cl_2$ (5 mol%)	-	DMF	100	34
2	$Pd(PPh_3)_2Cl_2$ (5 mol%)	-	DMF	120	42
3	$Pd(PPh_3)_2Cl_2$ (5 mol%)	-	DMF	140	37
4	$Pd(PPh_3)_4$ (5 mol%)	-	DMF	120	47
5	$Pd(dba)_2$ (5 mol%)	PPh_3 (10 mol%)	DMF	120	48
6	$Pd(OAc)_2$ (5 mol%)	PPh_3 (10 mol%)	DMF	120	-
7	$Pd(OAc)_2$ (5 mol%)	dppe (10 mol%)	DMF	120	-
8	$Pd(CH_3CN)_2Cl_2$ (5 mol%)	PPh_3 (10 mol%)	DMF	120	43
9[c]	$Pd(dba)_2$ (5 mol%)	PPh_3 (10 mol%)	DMF	120	45
10	$Pd(dba)_2$ (5 mol%)	PPh_3 (10 mol%)	DMSO	120	52
11	$Pd(dba)_2$ (5 mol%)	PPh_3 (10 mol%)	NMP	120	52
12	$Pd(dba)_2$ (5 mol%)	PPh_3 (10 mol%)	$PhCF_3$	120	21
13	$Pd(dba)_2$ (5 mol%)	PPh_3 (10 mol%)	Xylene	120	5
14	$Pd(dba)_2$ (5 mol%)	dppe (10 mol%)	DMSO	120	-
15	$Pd(dba)_2$ (5 mol%)	dppp (10 mol%)	DMSO	120	52
16	$Pd(dba)_2$ (5 mol%)	dppb (10 mol%)	DMSO	120	57
17	$Pd(dba)_2$ (5 mol%)	dppf (10 mol%)	DMSO	120	49
18	$Pd(dba)_2$ (5 mol%)	Johnphos (10 mol%)	DMSO	120	51
19	$Pd(dba)_2$ (5 mol%)	Xantphos (10 mol%)	DMSO	120	52
20	$Pd(dba)_2$ (5 mol%)	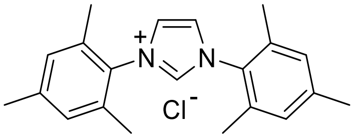 IMes-HCl (10 mol%)	DMSO	120	52

21	$Pd(dba)_2$ (5 mol%)	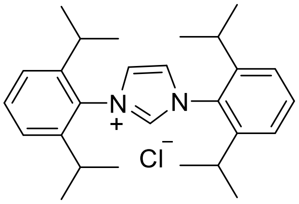 IPr-HCl (10 mol%)	DMSO	120	45
22	$Pd(dba)_2$ (5 mol%)	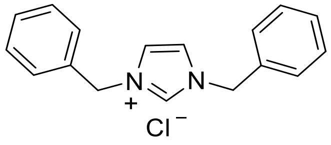 IBn-HCl (10 mol%)	DMSO	120	60
23	$Pd(dba)_2$ (5 mol%)	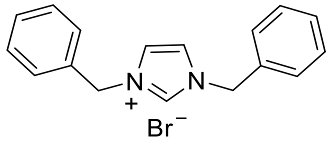 IBn-HBr (10 mol%)	DMSO	120	63
24	$Pd(dba)_2$ (5 mol%)	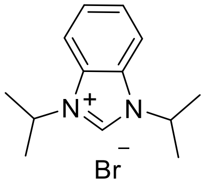 (10 mol%)	DMSO	120	60
25	$Pd(dba)_2$ (2 mol%)	IBn-HBr (4 mol%)	DMSO	120	65
**26**	**$Pd(dba)_2$ (1 mol%)**	**IBn-HBr (2 mol%)**	**DMSO**	**120**	**66**
27[d]	$Pd(dba)_2$ (1 mol%)	IBn-HBr (2 mol%)	DMSO	120	62

[a] Reactions were carried out with **1a** (0.1 M). [b] Yields were determined by GC analysis with biphenyl as the internal standard. [c] Reaction was carried out for 2 h. [d] Reaction was carried out with **1a** (0.05 M).

7.2 General procedure for batch, SFMT, and continuous-flow reactions

General procedure A for batch reactions

Under N_2 atomsphere, a solution of 4-Iodoanisole (57 mg, 0.25 mmol), $Pd(dba)_2$ (1.5 mg, 0.0025 mmol, 1 mol%), 1,3-bisbenzyl-imidazole bromide salt (1.6 mg, 0.005 mmol, 2 mol%), DIPEA (57 mg, 0.375 mmol, 1.8 equiv) and biphenyl (25 mg, 0.16 mmol) in DMSO (2.5 mL) was stirred at rt, and the solution was vacuumed and refilled with acetylene balloon. Then the reaction mixture was stirred at 120 ^{o}C for 40 mins. Subsequently, the mixture was cooled to rt. 1.0 mL of the solution was taken and quenched by distilled water (0.5 mL), extracted with ethyl acetate (1.0 mL), and the

organic phase was collected for GC analysis, which indicated the corresponding product **4a** in a 42% yield.

General procedure B for SFMT reactions

The acetylene tank is pressurized to ~15 psi higher than the desired back-pressure (10 psi) of the system. A flame dried 10 mL round bottom flask was equipped with a rubber septum and magnetic stir bar and was charged with aryl iodide (0.25 mmol), $Pd(dba)_2$ (1.5 mg, 0.0025 mmol, 1 mol%), 1,3-bisbenzyl-imidazole bromide salt (1.6 mg, 0.005 mmol, 2 mol%), DIPEA (57 mg, 0.375 mmol, 1.8 equiv) and biphenyl (25 mg, 0.16 mmol) in DMSO (2.5 mL). The mixture was bubble with Ar for 15 mins. An 8 mL, stainless steel Harvard Apparatus syringe was filled with the solution and then attached to the flow apparatus (syringe pump). The flow apparatus itself was set up at flow rate = 300 µL/min, and flow of acetylene was adjusted to create ~1:1 liquid/gas plugs. After approximate 2 mins equilibration, valves were closed, and the tubing (stainless steel, 1/16”, I.D. 0.03”, 300 cm, volume = 1.37 mL) was placed into the oil bath at 120 ^{o}C for 40 mins. Then the mixture in the tubing reactor was cooled to rt, and was washed out using DMSO (3.0 mL). The reaction mixture was added to water and extracted with ethyl acetate. After separation, the organic phase was collected for GC analysis to check the product yield (biphenyl as the internal standard).

General procedure C for continuous-flow reactions

The acetylene tank is pressurized to ~25 psi. A flame dried 10 mL round bottom flask was equipped with a rubber septum and magnetic stir bar and was charged with aryl iodide (1.0 mmol), $Pd(dba)_2$ (5.7 mg, 0.01 mmol, 1 mol%), 1,3-bisbenzyl-imidazole bromide salt (6.6 mg, 0.02 mmol, 2 mol%), DIPEA (228 mg, 1.8 mmol, 1.8 equiv) and biphenyl (100 mg, 0.64 mmol) in DMSO (5.0 mL). The mixture was bubbled with Ar for 15 mins. An 8 mL, stainless steel Harvard Apparatus syringe was filled with the solution and then attached to the continuous flow system. The tubing (stainless steel, SS-T4-S-035-6ME, O.D. 1/4", I.D. 4.57 mm, 20 cm) was packed with stainless steel powder and placed into the oil bath at 120 ^{o}C. The flow apparatus itself was set up as t_R = 40 mins, flow rate = 50 µL/min, and flow of acetylene was adjusted to create 1:1 liquid/gas plugs.

After approximately 120 mins for equilibration, the solution was collected for 1 h. The reaction mixture was poured into water (2 mL) and extract with ethyl acetate (1.0 mL X 2). After separation, the organic phase was collected for GC analysis to check the yield (biphenyl as the internal standard).

7.3 Large-scale continuous production

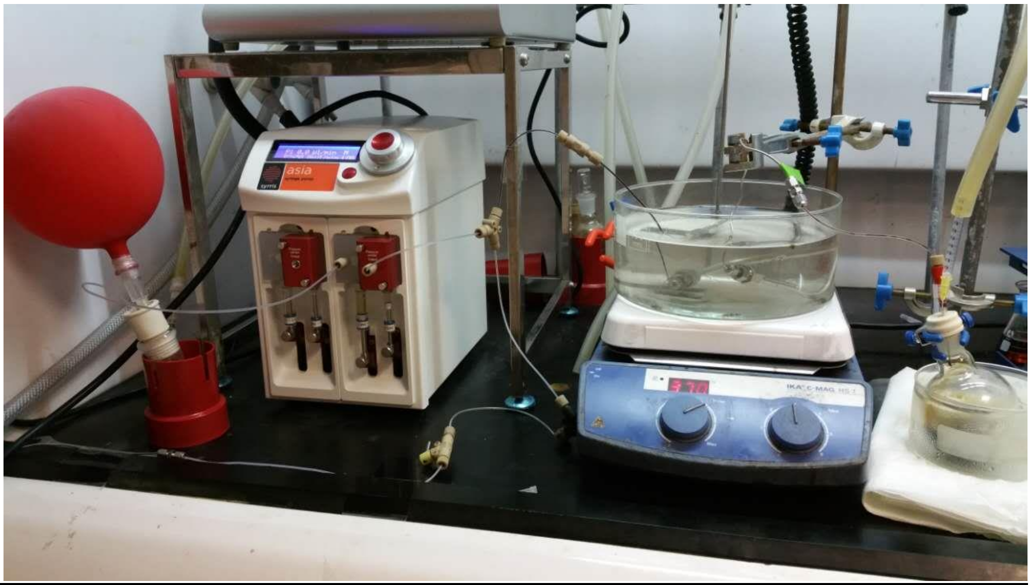

Figure S20. Continuous-flow setup for fulvene preparation.

The acetylene tank is pressurized to ~25 psi. A flame dried 250 mL round bottom flask was equipped with a rubber septum and magnetic stir bar and was charged with 4-iodoanisole (1.14 g, 5.0 mmol), $Pd(dba)_2$ (30 mg, 0.05 mmol, 1 mol%), 1,3-bisbenzyl-imidazole bromide salt (33 mg, 0.1 mmol, 2 mol%), DIPEA (1.16 g, 9 mmol, 1.8 equiv) and biphenyl (0.49 g, 3.2 mmol) in DMSO (50.0 mL). The mixture was bubbled with acetylene for 15 mins. The same solution was made twice. When the solution in RBF was nearly pumped empty, the fresh prepared solution was refilled to avoiding decomposition of Pd catalyst during their stay at room temperature. As shown in Figure S20, an Asia pump was filled with the solution and then attached to the flow apparatus (syringe pump). The tubing (stainless steel, SS-T4-S-035-6ME, O.D. 1/4", I.D. 4.57 mm, 20 cm) was packed with stainless steel powder and placed into the oil bath at 120 ^{o}C. The flow apparatus itself was set up with t_R = 40 mins, flow rate = 50 μL/min, and flow of

acetylene was adjusted to create 1:1 liquid/gas plugs. After approximately 120 mins of equilibration, the solution was collected for 36 h, quenched with distilled water (100 mL), and extracted by ether (150 mL x 3). The combined organic phase was dried with $MgSO_4$. After evaporation with reduced pressure below 20 °C, the red residue was purified by flash chromatography (eluent: hexane), affording the red solid product 1.1 g, in 55% yield.

7.4 New compound characterization

Example of calibration of GC yields

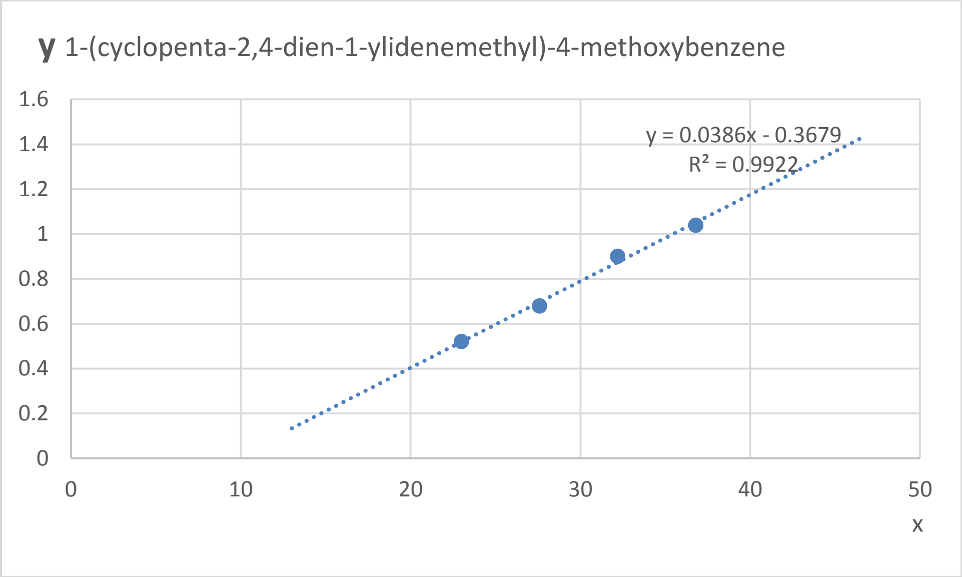

Figure S21. Calibration curve of 1-(cyclopenta-2,4-dien-1-ylidenemethyl)-4-methoxybenzene.

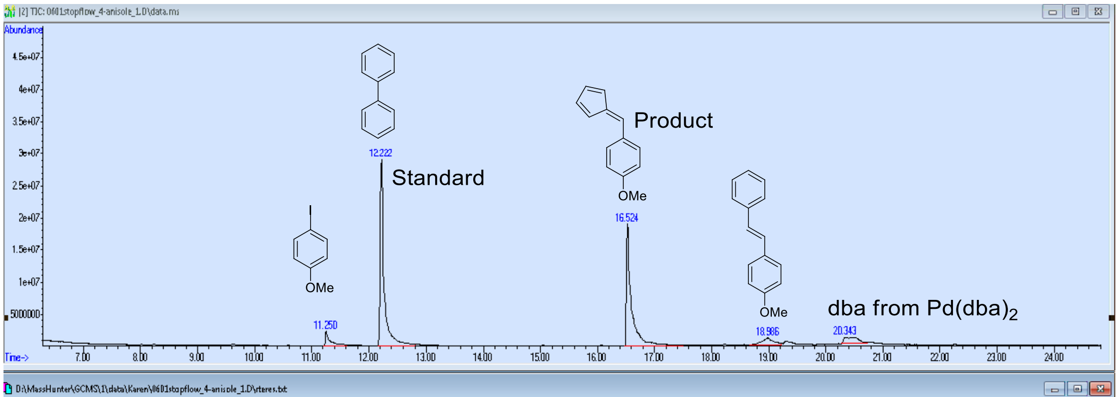

Area Percent Report

Data Path : D:\MassHunter\GCMS\1\data\Karen\
Data File : 0601stopflow_4-anisole_1.D
Acq On : 01 Jun 2016 14:25

peak #	R.T. min	first scan	max scan	last scan	PK TY	peak height	corr. area	corr. % max.	% of total
1	11.250	1195	1204	1289	M2	2270592	136205846	9.73%	4.497%
2	12.222	1422	1440	1580	M2	29135227	1399507659	100.00%	46.209%
3	16.524	2472	2483	2720	M	19198527	1120228345	80.04%	36.988%
4	18.986	3001	3081	3138	M4	1210079	182416306	13.03%	6.023%
5	20.343	3391	3410	3503	M4	1052321	190274706	13.60%	6.283%

Sum of corrected areas: 3028632941

Figure S22. GC spectrum of the crude mixture.

Follow the same method as mention in Figure S19, based on Figure S22, it was calculated y_{4a} = 0.805, x_{4a} = 30.4. Therefore, the yield of **4a** = 30.4/46 = 66%.

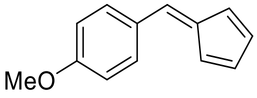

1-(cyclopenta-2,4-dien-1-ylidenemethyl)-4-methoxybenzene (4a)

Batch: Followed the general procedure **A** in batch, obtained **4a** in 42% yield.

Stop-flow : Followed the general procedure **B** in SFMT reactors with 4-iodoanisole (58.5 mg, 0.25 mmol), GC analysis of the crude product mixture indicated a 66% yield of **4a**.

^{1}H NMR ($CDCl_3$, 400 M) δ 7.60 (d, *J* = 8.8 Hz, 2H), 7.18 (s, 1H), 6.86 (d, *J* = 8.8 Hz, 2H), 6.76-6.75 (m, 1H), 6.70-6.68 (m, 1H), 6.52-6.50 (m, 1H), 6.36-6.34 (m, 1H), 3.86 (s, 3H).

Characterization data of compound **4a** have been reported in previous literature.[19]

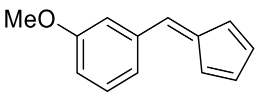

1-(cyclopenta-2,4-dien-1-ylidenemethyl)-3-methoxybenzene (4b)

Followed the general procedure **B** in SFMT reactors with 3-iodoanisole (58.5 mg, 0.25 mmol), GC analysis of the crude product mixture indicated a 42% yield of **4b**.

Characterization data of compound **4b** have been reported in previous literature.[19]

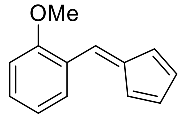

1-(cyclopenta-2,4-dien-1-ylidenemethyl)-2-methoxybenzene (4c)

Followed the general procedure **B** in SFMT reactors with 2-iodoanisole (58.5 mg, 0.25 mmol), GC analysis of the crude product mixture indicated a 60% yield of **4c**.

Characterization data of compound **4c** have been reported in previous literature.[20]

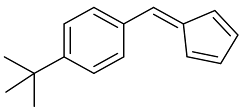

1-(tert-butyl)-4-(cyclopenta-2,4-dien-1-ylidenemethyl)benzene (4d)

Followed the general procedure **B** in SFMT reactors with 1-iodo-4-(tert-butyl)benzene (65.0 mg, 0.25 mmol), GC analysis of the crude product mixture indicated a 56% yield of **4d**.

Characterization data of compound **4d** have been reported in previous literature.[21]

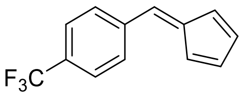

1-(cyclopenta-2,4-dien-1-ylidenemethyl)-4-(trifluoromethyl) benzene (4e)

Followed the general procedure **B** in SFMT reactors with 1-iodo-4-(trifluoromethyl)benzene (68.0 mg, 0.25 mmol), GC analysis of the crude product mixture indicated a 40% yield of **4e**.

Characterization data of compound **4e** have been reported in previous literature.[19]

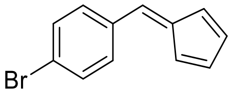

1-(cyclopenta-2,4-dien-1-ylidenemethyl)-4-bromobenzene (4f)

Followed the general procedure **B** in SFMT reactors with 1-bromo-4-iodobenzene (70.7 mg, 0.25 mmol), GC analysis of the crude product mixture indicated a 62% yield of **4f**.

^{1}H NMR ($CDCl_3$, 400 M) δ 7.55 (d, J = 8.4 Hz, 2H), 7.44 (d, J = 8.8 Hz, 2H), 7.12 (s, 1H), 6.70-6.68 (m, 1H), 6.64-6.62 (m, 1H), 6.54-6.52 (m, 1H), 6.32-6.30 (m, 1H) ; ^{13}C NMR ($CDCl_3$, 100 M) δ 145.8, 136.5, 135.9, 135.7, 132.0, 131.9, 131.2, 127.1, 123.5, 120.0. GC/MS (m/z, relative intensity) : 232 (M+, 25),153 (100).

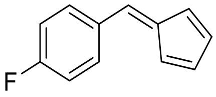 **1-(cyclopenta-2,4-dien-1-ylidenemethyl)-4-fluorobenzene (4g)**

Followed the general procedure **B** in SFMT reactors with 1-fluoro-4-iodobenzene (70.7 mg, 0.25 mmol), GC analysis of the crude product mixture indicated a 50% yield of **4g**. Characterization data of compound **4g** have been reported in previous literature.[19]

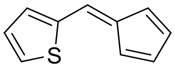 **2-(cyclopenta-2,4-dien-1-ylidenemethyl)thiophene (4h)**

Followed the general procedure **B** in SFMT reactors with 2-iodothiophene (52.5 mg, 0.25 mmol), GC analysis of the crude product mixture indicated a 48% yield of **4h**. Characterization data of compound **4h** have been reported in previous literature.[22]

8. Visible-Light Promoted Synthesis of Styrene Derivatives Using Acetylene

8.1 SFMT Reactor Setup

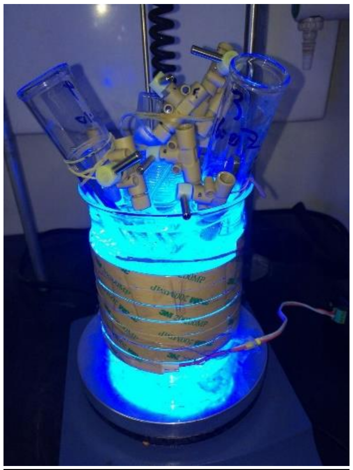

Figure S23. Stop-flow setup for fluorinated styrene screening and preparation.

8.2 Reaction optimization

Table S4. Reaction optimization of synthesis of styrene derivatives (catalyst, base and solvent screening).

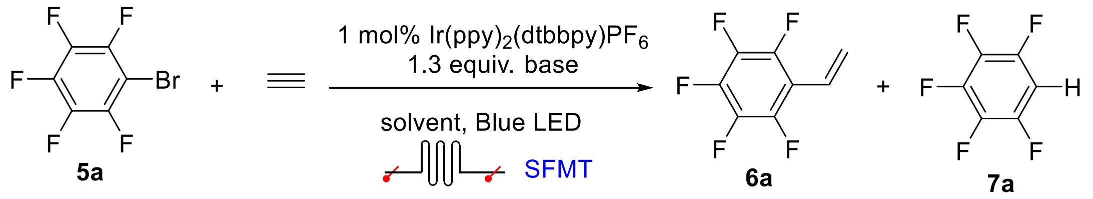

Entry	Catalyst	Base	Solvent	Conversion [%][a]	Ratio (**6a**:**7a**)[a]
1	$Ir(ppy)_2(dtbbpy)PF_6$	*i*Pr_2EtN	CH_3CN	94	1.8 : 1
2	$Ir(ppy)_3$	*i*Pr_2EtN	CH_3CN	69	1.8 : 1
3	Ir(dF-CF_3-ppy)$_2$(dtbbpy)PF_6	*i*Pr_2EtN	CH_3CN	73	1.6 : 1
4	$Ru(bpy)_3Cl_2$	*i*Pr_2EtN	CH_3CN	51	1.4 : 1
5	eosin Y	*i*Pr_2EtN	CH_3CN	55	1.4 : 1
6	[Acr^+-Mes]ClO_4^-	*i*Pr_2EtN	CH_3CN	0	-
7	$Ir(ppy)_2(dtbbpy)PF_6$	Et_3N	CH_3CN	80	1 : 1
8	$Ir(ppy)_2(dtbbpy)PF_6$	DBU	CH_3CN	62	1 : 4.2
9	$Ir(ppy)_2(dtbbpy)PF_6$	DMEDA	CH_3CN	92	1 : 2.1
10	$Ir(ppy)_2(dtbbpy)PF_6$	Bu_3N	CH_3CN	100	1 : 11
11	$Ir(ppy)_2(dtbbpy)PF_6$	TMEDA	CH_3CN	100	1 : 20.5
12	$Ir(ppy)_2(dtbbpy)PF_6$	DABCO	CH_3CN	23	1 : 4.8
13	$Ir(ppy)_2(dtbbpy)PF_6$	*t*Butylamine	CH_3CN	0	-
14	$Ir(ppy)_2(dtbbpy)PF_6$	*i*Pr_2EtN	THF	100	0 : 1
15	$Ir(ppy)_2(dtbbpy)PF_6$	*i*Pr_2EtN	DMF	100	0 : 1

[a] Conversions and ratios were determined based on ^{19}F NMR analysis of the crude product mixtures.

Table S5. Reaction optimization of synthesis of styrene derivatives (with TEMPO)

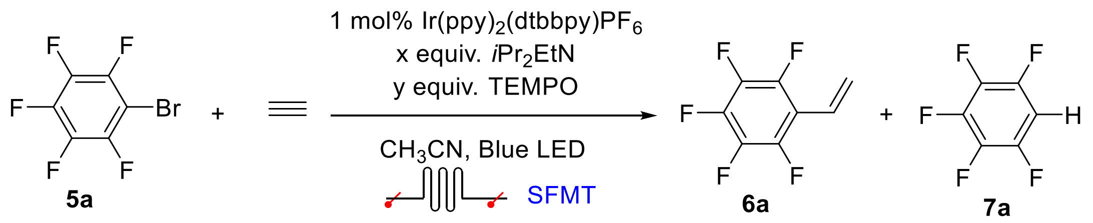

Entry	TEMPO [y]	*i*Pr_2NEt [x]	t [h]	*T* [°C]	Conversion [%][a]	Ratio [**6a**:**7a**][a]
1	1.0	1.3	3	50	80	3.2 : 1
2[b]	1.0	1.3	3	50	44	2.8 : 1
3	1.0	1.3	1	60	74	3.8 : 1
4	1.0	1.3	3	60	93	3.2 : 1
5	0.2	1.3	3	60	100	4 : 5
6	0.5	1.3	3	60	100	1.6 : 1
7	1.2	1.3	3	60	78	3 : 1
8	2.0	1.3	3	60	23	2.3 : 1
9	1.0	1.5	3	60	100	1.9 : 1

10	1.0	2.0	3	60	100	1 : 5
**11[c]**	**1.0**	**1.3**	**3**	**60**	**97**	**3.6 : 1**

[a] Conversions and ratios were determined based on ^{19}F NMR analysis of the crude product mixtures. [b] Without degassing. [c] with 20 Psi BPR.

8.3 General procedure for batch, and SFMT reactions

General procedure A for batch reaction.

Under Argon atomsphere, a solution of bromopentafluorobenzene (31.2 ul, 0.25 mol), $Ir(ppy)_2(dtbbpy)PF_6$ (2.3 mg, 0.0025 mmol, 1 mol%), TEMPO (39.0 mg, 0.25 mmol) and DIPEA (56.0 µL, 0.325 mmol, 1.3 equiv) with CH_3CN (2.5 mL) were introduced into a 15 mL-Schlenk tube equipped with a magnetic stir bar. The mixtures were bubbled with argon balloon and acetylene balloon carefully in an ice-water bath for 10 and 2 minutes respectively, and then placed in a water bath with acetylene balloon for 3 h at 60 °C under a blue LED source. The residue was then diluted with $CDCl_3$, and directly analyzed by GC-Ms and ^{19}F NMR.

General procedure B for SFMT reaction.

Under Argon atomsphere, a solution of fluorinated aryl bromide **5** (0.30 mol), $Ir(ppy)_2(dtbbpy)PF_6$ (2.8 mg, 0.003 mmol, 1 mol%) and TEMPO (46.8 mg, 0.30 mmol) with CH_3CN (3.0 mL) were introduced into a 10 mL silicon septa vial. The mixtures were bubbled with argon balloon carefully in an ice-water bath for 10 minutes. DIPEA (56.0 µL, 0.325 mmol, 1.3 equiv) was then added into the mixture via Argon bubbling for another 5 mintues. An 8 mL, stainless steel Harvard Apparatus syringe was filled with the solution and then attached to the flow apparatus (syringe pump). The acetylene tank is pressurized to ~10 psi higher than the desired back-pressure (20 psi) of the system. The flow apparatus itself was set up at flow rate = 100 µL/min, and flow of acetylene was adjusted to create around 2:1 gas/liquid plugs. After approximate 3 minutes, both shut-off valves were closed, and the stop-flow micro tubing reactor (total volume 0.65 ml, 0.065 mmol) which was made of HPFA tubing (O.D. 1/16", I.D. 0.03", 300 cm, volume = 1.37 mL) was placed under a blue LED source in the water bath at 60 ^{o}C for 3 h. The same procedure was applied for three other micro tubing reactors. Then the mixtures of four stop-flow micro tubing reactors (total volume 2.6 ml, 0.26 mmol) were combineed

together. The solvent was carefully removed under reduced pressure due to volatile of products, and the residue was firstly purified by flash column chromatography on silica gel, and then further purified by GPC (Gel Permeation Chromatography).

8.4 New compound characterization

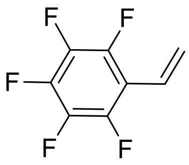

Compound 2,3,4,5,6-Pentafluorostyrene (6a): Prepared according to procedure B for SFMT reactions: (35 mg, 70% yield). Characterization of compound **6a** has been reported in previous literature.[23]

Notes: Carefully removed the solvent due to the volatility of **6a.**

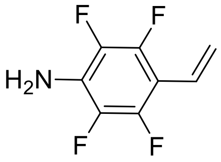

Compound 2,3,5,6-tetrafluoro-4-vinylaniline (6b): Prepared according to procedure B for SFMT reactions: (26 mg, 52% yield). ^{1}H NMR (400 MHz, $CDCl_3$) δ 6.59 (dd, *J* = 18.0, 12.0 Hz, 1H), 5.91 (d, *J* = 18.0 Hz, 1H), 5.49 (d, *J* = 12.0 Hz, 1H), 4.01 (bs, 2H); ^{19}F NMR (376 MHz, $CDCl_3$) δ -146.28 – -146.37 (m, 2F), -163.17 – -163.26 (m, 2F). ^{13}C NMR (125 MHz, $CDCl_3$) δ 145.08 (dddd, *J* = 245.0, 11.9, 8.3, 3.6 Hz), 136.59 (dddd, *J* = 236.2, 9.4, 5.6, 3.9 Hz), 125.06 (tt, *J* = 14.4, 3.7 Hz), 122.49 (t, *J* = 2.0 Hz), 119.67 (t, *J* = 7.7 Hz), 104.96 (t, *J* = 13.8 Hz). HRMS (ESI) m/z calcd for $C_8H_4F_4N$ $[M - H]^-$ 190.0258, found: 190.0289.

Notes: Carefully removed the solvent due to the volatility of **6b.**

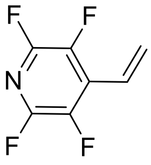

Compound 2,3,5,6-tetrafluoro-4-vinylpyridine (6c): Prepared according to procedure B for SFMT reactions: (22 mg, 48% yield). Characterization of compound **6c** has been reported in previous literature.[24]

Notes: Carefully removed the solvent due to the volatility of **6c.**

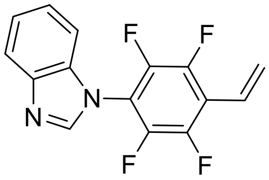

Compound 1-(2,3,5,6-tetrafluoro-4-vinylphenyl)-1*H*-benzo[*d*]imidazole) (6d): Prepared according to procedure B for SFMT reactions: (46 mg, 61% yield). ^{1}H NMR (400 MHz, $CDCl_3$) δ 8.03 (s, 1H), 8.02-7.88 (m, 1H), 7.41-7.35 (m, 2H), 7.28-7.25 (m, 1H), 6.76 (dd, *J* = 18.0, 12.0 Hz, 1H), 6.24 (d, *J* = 18.0 Hz, 1H), 5.85 (d, *J* = 12.0 Hz,

1H). ^{19}F NMR (376 MHz, CDCl3) δ -141.70 – -146.80 (m, 2F), -147.11 – -147.21 (m, 2F). ^{13}C NMR (125 MHz, $CDCl_3$) δ 145.05 (dddd, *J* = 250.5, 12.4, 7.2, 3.2 Hz), 143.10, 142.36, 142.28 (ddt, *J* = 251.3, 15.5, 4.0 Hz), 133.37, 125.30 (t, *J* = 7.9 Hz), 124.46, 123.48, 121.63, 120.80, 117.39 (t, *J* = 13.6 Hz), 113.83 (tt, *J* = 14.4, 2.6 Hz), 110.4. HRMS (ESI) m/z calcd for $C_{15}H_9F_4N$ $[M + H]^+$, 293.0696, found: 293.0700.

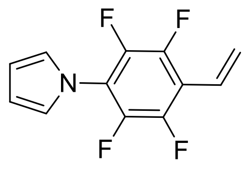

Compound 1-(2,3,5,6-tetrafluoro-4-vinylphenyl)-1*H*-pyrrole) (6e): Prepared according to procedure B for SFMT reactions: (39.5 mg, 63% yield). ^1H NMR (400 MHz, $CDCl_3$) δ 6.96 (q, *J* = 2.0, 2H), 6.72 (dd, *J* = 18.0, 12.0 Hz, 1H), 6.42 (t, *J* = 2.0 Hz, 2H), 6.16 (d, *J* = 18.0 Hz, 1H), 5.76 (d, *J* = 12.0 Hz, 1H). ^{19}F NMR (376 MHz, $CDCl_3$) δ -143.22 – -143.31 (m, 2F), -151.10 – -151.19 (m, 2F). ^{13}C NMR (125 MHz, $CDCl_3$) δ 145.16 (dddd, *J* = 248.7, 12.6, 7.6, 4.3 Hz), 141.40 (ddt, *J* = 248.0, 15.7, 3.4 Hz), 123.94 (t, *J* = 7.8 Hz), 122.31, 121.85, 119.13 (t, *J* = 12.8 Hz), 114.59 (t, *J* = 13.7 Hz), 110.60. HRMS (APCI) m/z calcd for $C_{12}H_7F_4N$ 241.0509, found: 241.0506.

Notes: Carefully removed the solvent due to the volatility of **6e.**

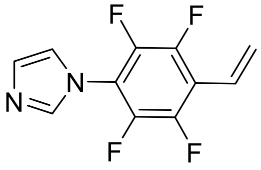

Compound 1-(2,3,5,6-tetrafluoro-4-vinylphenyl)-1*H*-imidazole) (6f): Prepared according to procedure B for SFMT reactions: (41 mg, 65% yield). ^1H NMR (500 MHz, $CDCl_3$) δ 7.81 (s, 1H), 7.30-7.26 (m, 2H)), 6.71 (dd, *J* = 18.0, 12.0 Hz, 1H), 6.19 (d, *J* = 18.0 Hz, 1H), 5.81 (d, *J* = 12.0 Hz, 1H), 1.88 (s, 1H). ^{19}F NMR (376 MHz, $CDCl_3$) δ -141.99 – -142.09 (m, 2F), -149.92 – -150.02 (m, 2F). ^{13}C NMR (125 MHz, $CDCl_3$) δ 145.05 (dddd, *J* = 250.4, 12.5, 7.2, 4.3 Hz), 141.12 (dddd, *J* = 249.6, 7.0, 4.1, 2.9 Hz), 137.67, 130.06, 125.02 (t, *J* = 7.9 Hz), 121.54, 120.01, 116.36 (t, *J* = 13.6 Hz), 115.40 (t, *J* = 12.7 Hz). HRMS (ESI) m/z calcd for $C_{11}H_7F_4N$ $[M + H]^+$, 243.0540, found: 243.0543.

Notes: Compound of **6f** is easy to isomerize under acidic conditions.

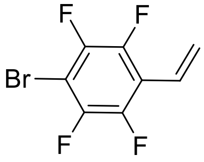

Compound 4-Bromo-2,3,5,6-tetrafluorostyrene (6g): Prepared according to procedure B for SFMT reaction: (34 mg, 51% yield). Characterization of compound **6g** has been reported in previous

literature.[25]

Notes: Carefully removed the solvent due to the volatility of **6g.**

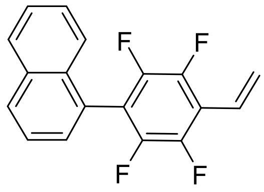

Compound 1-(2,3,5,6-tetrafluoro-4-vinylphenyl)naphthalene (6h): Prepared according to procedure B for SFMT reactions: (35 mg, 45% yield). ^{1}H NMR (500 MHz, $CDCl_3$) δ 8.02-7.94 (m, 2H), 7.61-7.48 (m, 5H), 6.83 (dd, *J* = 18.0, 12.0 Hz, 1H), 6.23 (d, *J* = 18.0 Hz, 1H), 5.80 (d, *J* = 12.0 Hz, 1H). ^{19}F NMR (376 MHz, $CDCl_3$) δ -141.42 – -141.51 (m, 2F), -143.94 – -144.03 (m, 2F). ^{13}C NMR (125 MHz, $CDCl_3$) δ 144.76 (dddd, *J* = 248.9, 10.5, 6.3, 3.9 Hz), 144.31 (dddd, *J* = 244.6, 9.7, 5.8, 3.9 Hz), 133.65 131.51, 129.89, 128.77, 128.54, 126.89, 126.28, 125.17, 124.87, 123.80 (t, *J* = 7.7 Hz), 122.52, 117.81 (t, *J* = 19.2 Hz), 116.42 (t, *J* = 13.4 Hz). HRMS (APCI) m/z calcd for $C_{18}H_{10}F_4$ 302.0713, found: 302.0716.

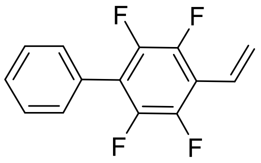

Compound 1-(2,3,5,6-tetrafluoro-4-vinylphenyl)benzene (6i): Prepared according to procedure B for SFMT reactions: (35 mg, 53% yield). ^{1}H NMR (500 MHz, $CDCl_3$) δ 7.53-7.44 (m, 5H), 6.76 (dd, *J* = 18.0, 12.0 Hz, 1H), 6.17 (d, *J* = 18.0 Hz, 1H), 5.75 (d, *J* = 12.0 Hz, 1H). ^{19}F NMR (376 MHz, $CDCl_3$) δ -144.27 – -144.36 (m, 2F), -145.35 – -145.44 (m, 2F). ^{13}C NMR (125 MHz, $CDCl_3$) δ 144.90 (dddd, *J* = 248.1, 10.4, 6.5, 3.9 Hz), 143.83 (dddd, *J* = 244.4, 9.8, 5.3, 4.0 Hz), 130.13(t, *J* = 1.7 Hz), 129.09, 128.56, 127.44, 123.57 (t, *J* = 7.8 Hz), 122.45, 119.11 (t, *J* = 16.8 Hz), 115.73 (t, *J* = 13.5 Hz). HRMS (APCI) m/z calcd for $C_{14}H_8F_4$ 252.0557, found: 252.0560.

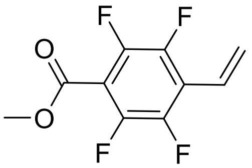

Compound methyl 2,3,5,6-tetrafluoro-4-vinylbenzoate (6j): Prepared according to procedure B for SFMT reactions: (26 mg, 43% yield). ^{1}H NMR (500 MHz, $CDCl_3$) δ 6.70 (dd, *J* = 18.0, 12.0 Hz, 1H), 6.21 (d, *J* = 18.0 Hz, 1H), 5.82 (d, *J* = 12.0 Hz, 1H), 3.98 (s, 3H). ^{19}F NMR (376 MHz, $CDCl_3$) δ -140.43 – -140.53 (m, 2F), -142.72 – -142.82 (m, 2F). ^{13}C NMR (125 MHz, $CDCl_3$) δ 160.21, 145.88-145.46 (m), 143.84-143.46 (m),

125.72 (t, J = 7.9 Hz), 121.94, 119.72 (t, J = 13.2 Hz), 110.58 (t, J = 15.7 Hz). HRMS (APCI) m/z calcd for $C_{10}H_6F_4N$ 362.1671, found: 362.1673.

Notes: Carefully removed the solvent due to the volatility of **6j.**

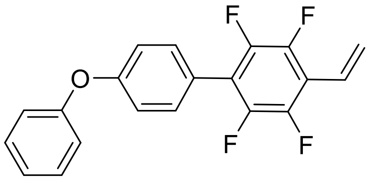

Compound 2,3,5,6-tetrafluoro-4'-phenoxy-4-vinyl-1,1'-biphenyl (6k): Prepared according to procedure B for SFMT reactions: (33 mg, 37% yield). ^{1}H NMR (500 MHz, $CDCl_3$) δ 7.46-7.37 (m, 4H), 7.19-7.15 (m, 1H), 7.11-7.08 (m, 4H), 6.75 (dd, J = 18.0, 12.0 Hz, 1H), 6.16 (d, J = 18.0 Hz, 1H), 5.74 (d, J = 12.0 Hz, 1H). ^{19}F NMR (376 MHz, $CDCl_3$) δ -144.28 – -144.37 (m, 2F), -145.52 – -145.61 (m, 2F). ^{13}C NMR (125 MHz, $CDCl_3$) δ 158.36, 156.22, 144.93 (dddd, J = 248.3, 10.5, 6.2, 4.5 Hz), 143.84 (ddt, J = 244.1, 14.6, 4.4 Hz), 131.65, 129.92, 124.03, 123.51(t, J = 7.8 Hz), 122.46, 121.74, 119.73, 118.56 (t, J = 16.6 Hz), 118.18, 115.50 (t, J = 13.5 Hz). HRMS (APCI) m/z calcd for $C_{20}H_{12}F_4O$ 344.0819, found: 344.0820.

9. Trouble Shooting: Frequently Asked Questions

9.1 The SFMT reactor platform

Question 1: What is an SFMT reactor platform?

The "stop-flow" micro-tubing (SFMT) reactor platform is a design adapted from the continuous flow system, with added batch elements. Instead of making the reaction mixture flow continuously, the SFMT platform allows it to pause at will. This is achieved by the usage of two shut-off valves. The system includes several parts. One syringe pump is used for pumping the constant reagents during screening. Another syringe pump is applied for pumping variable reagents for screening, with switchable syringes. A gas line is applied if gaseous reagents are involved in the reaction. All the reagents will meet at a mixer and flow into the micro-tubing reactor with two shut-off valves equipped at the two ends of each reactor. A back-pressure regulator (BPR) is used to control the system pressure. A heating bath will be utilized as the off-line heating source. This platform is designed for the reaction screening purpose for the continuous-flow synthesis.

Question 2: How does the SFMT reactor platform works?

The reagents will be pumped into the micro-tubing reactors by the flow system. After the reagents pass through the BPR, the shut-off valves at the two ends of the reactor will be closed, and the reactor holds almost the same pressure as indicated by the BPR. The reactor will then be disconnected from the flow system. The next empty micro-tubing reactor will be connected to the flow system to fill in the reagents. After all the micro-tubings have been filled with planned reagents, they will be moved to an off-line heating bath for parallel heating. Finally, after desired heating period, the reaction mixture in

each micro-tubing will be washed out by organic solvents to individual sample vials. The crude mixture will be subjected to GC, LC, and NMR analysis to identify the reaction results.

Question 3: What kind of pumps should I choose for the SFMT reactor platform?

Different pumps can be chosen for different reaction patterns. In our study, we used Harvard or KDS syringe pumps in our SFMT reactor system as they can work precisely under high-pressure, and syringes with several volume sizes (2.5 mL, 8 mL, 20 mL, 50 mL, and 100 mL) are available for choosing. Peristaltic pumps will be good for the SFMT system for reactions at ambient pressure. We are targeting to use MilliGat pump for a proposed automated SFMT system to switch reagents and solvents.

Question 4: How to introduce gas reagents into a SFMT reactor platform?

Gaseous reagents will be supplied from gas tanks with regulators. The gas is introduced into the flow system using the Swagelok stainless steel tubing connected to a Y mixer. The flow of gas is regulated with a needle valve (IDEX) and is visually controlled to 1:1 to 2:1 gas to liquid volume slugs. If a precise control of gas flow rate is required, a digital Mass-Flow controller (SmartTrak 100) is applied.

Question 5: What kind of mixers should I choose for my reactions?

In our study, the T or Y mixers with thru-hole 0.02 inch from IDEX (P-727, P-512) worked well for most of cases. For homogeneous reactions, to attempt improved mixing efficiency, more advanced micro-mixers such as the IMM single mixer or Yamatake YM-1 mixer can be utilized.[26]

Question 6: What kind of reactors should I choose for my reactions?

Micro-tubings made by any material can potentially be applied to the SFMT platform, such as polymers, stainless-steel (SS), glass, etc. In our study, SS-tubings were used for reactions with temperatures above 100 ^{o}C as it can withstand the heat and pressure. HPFA tubings were used for ambient/ cold/ temperatures up to 120 ^{o}C reactions. The HPFA tubing is also applied for photoredox reactions due to its transparency and high surface volume of reagents exposed to the light source. Glass tubings can also be applied to light-promoted reactions. For gas/liquid reactions, gases will pass through the HPFA tubing during heating (e.g. 100 ^{o}C) at high pressure (e.g. 100 psi). If gas leaking is observed, HPFA tubing can be replaced with SS-tubing. Glass tubing and TEFZEL tubing can be used if light reactions are involved to stand higher temperature and pressure.

Question 7: What is the size of the micro-tubing reactor?

The inner diameter of the micro-tubing reactor is normally less than 1mm. The volume of the tubing can be calculated by $V=\pi r^2 L$, and can be any volume in principle. For the reaction screening purpose in our study, we normally used tubings with approximate 1.37 mL volume.

Question 8: What is the reaction scale in the SFMT reactor?

The scale of one reaction in the SFMT reactor is determined by the reactor size, the reaction nature, and concentration. For instance, in the case of homogeneous liquid

reaction, to achieve a 1 mmol production (based on 100% yield) with a 0.2 M concentration reaction mixture, ~ 6.17 meter of micro-tubing with 0.04 inch I.D. is needed. If the tubing I.D. is 0.062 inch, ~ 2.56 meter of the micro-tubing is needed. In the case of gas/liquid reaction, the tubing length normally needs to be doubled. However, reactions at 0.2 mmol scale are instructive enough for evaluation. However, the SFMT reactor can be used for gram scale synthesis. The reaction scale can also be increased by numbering-up strategy.

Question 9: What kind of BPRs should I choose for my reactions?

BPRs from IDEX were utilized in our study as there are many choices (5, 20, 40, 75, 100, 250, 500, 750, 1000 psi), and they can be used jointed to achieve a certain amount of back-pressure. Zaiput BPR is used for tunable pressure. Swagelok BPR can also be used for this purpose.

Question 10: How to proceed the off-line heating or cooling?

If the screening is conducted at high temperature, all the micro-tubing reactors can be filled with reagents to be tested at room temperature first, and then heated together in an oil bath. If the screening is conducted at low temperature, the reagents will be filled into the micro-tubing reactor in a cooling bath at the required reaction temperature with reaction time started to be calculated once the micro-reactor was fully filled.

Question 11: How do I monitor the reaction in a SFMT reactor?

During the screening period, the SFMT reactor is not monitored. After reactions are quenched, the crude mixtures will be subjected to TLC, NMR, and GC/LC-MS analysis.

Question 12: What is the exact pressure in a SFMT reactor?

The pressure in a SFMT reactor should be roughly equal to the set back-pressure after the shut-off valves are closed at room temperature. During heating, the pressure inside the tubing will increase. It can be roughly calculated by $P_1/T_1 = P_2/T_2$ according to Amontons's Law.

Question 13: Why continuous flow resulted in slightly better yields than SFMT reactors?

This can be explained by the excellent mixing efficiency due to the toroidal vortices generated in continuous heterogeneous segmented flow. However, results from SFMT reactors are instructive enough for the purpose of reaction discovery/optimization.

Question 14: Do I need to wash the system between two different reactions?

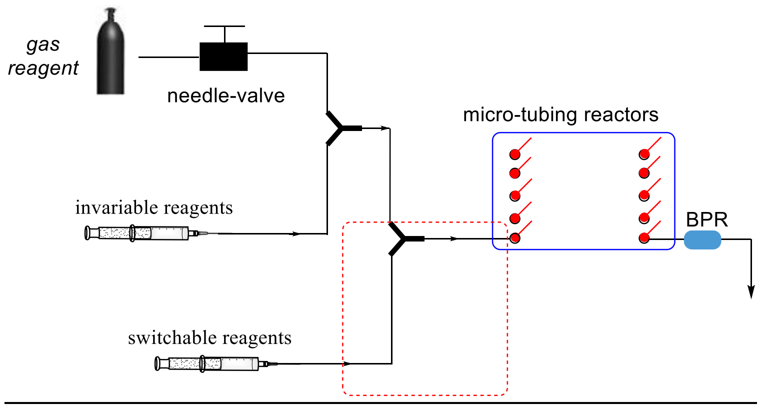

The part in red rectangle needs to be washed every time between two different reactions. You can design this part of tubing as short as possible.

Question 15: Can the SFMT system become automatic?

Yes. We are currently proposed an automated prototype of the SFMT system. A typical system will include: one syringe pump for constant reagents/solvents, and one MilliGat pump for switchable reagents/catalysts/solvents, one gas mass flow controller, a four-way mixer, two multi-channel valves, one set of parallel stop-flow micro-tubing reactors, one oil tank or cooler to control temperature, one pressure sensor, one back pressure regulator, and a vial holder for sample vials. The MilliGat pump will be used for switchable reagents as it is able to achieve automated pumping among different reagents at high pressure avoiding refilling or cross-contamination.[27] All the shut-off valves for stop-flow reactors will be mounted on a stainless steel mantle, and the tubing reactors will be connected and immersed in the oil tank for heating or a cooling bath for low temperature reactions. A computer system will be used to control the automated system.

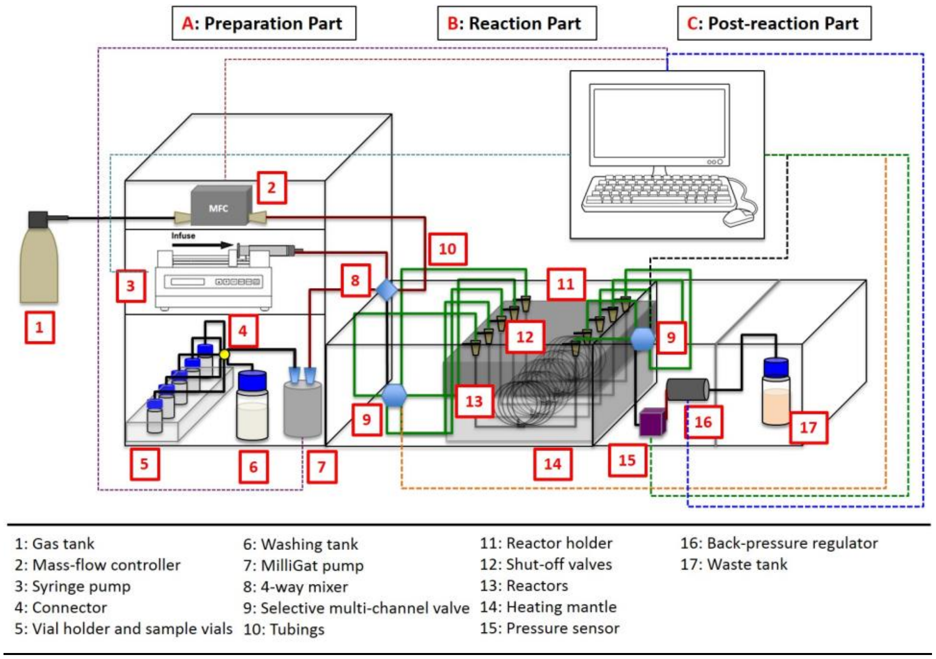

9.2 Sonogashira coupling

Question 1: What are other methods to make terminal alkynes?

Terminal alkynes can also be generated from aldehyde by Corey-Fuchs reaction or using the Ohira-Bestmann reagent. They also can be synthesized from elimination of di-halogenated alkanes. However, these transformations require expensive starting materials or reagents, which makes the large-scale industrial production of terminal alkynes through these methods impossible. To prepare terminal alkynes or unsymmetric internal alkynes from halogenated precursors, a protected acetylene, such as trimethylsilyl-acetylene or 2-methyl-3-butyn-2-ol,[28] was normally applied, followed by the release of the protecting group.

Question 2: What if the reaction does not proceed to completion?

If there is still starting bromide or iodide compounds left, the reaction can be proceeded with longer reaction time or with slightly higher temperature to push to a full conversion.

Question 3: Is there a trend to predict the reaction selectivity of terminal alkynes vs internal alkynes?

Aryl iodides with electron-donating aryl rings and vinyl bromides generally afforded very good selectivity. However, aryl iodides with electron-withdrawing group afforded lower selectivity. This is probably because the generated terminal alkynes with electron-withdrawing group is more acidic and easier to generate the copper intermediate than acetylene to undergo further Sonogashira coupling with aryl iodides again.

Question 4: What if the reaction selectivity is not good?

Reducing the reaction temperature or the concentration of starting halogen compounds should help to increase the selectivity.

Question 5: How sensitive is this transformation towards air?

The reactions were not performed strictly anhydrously. AR ACS grade DMSO was used in our study.

Question 6: What is the concentration of starting materials for the success of the selective coupling?

0.1 M concentration was applied in our “stop-flow” or “continuous-flow” process. Compared to Vasilevsky’s batch study at 0.033 M, our concentration is much higher and more practical.

9.3 Fulvene synthesis

Question 1: How stable are these fulvene products?

Fulvenes are oxygen and heat-sensitive compounds. However, they can be purified by a quick flash column chromatography over silica gel.

Question 2: Any care needs to be taken during product purification and storage to avoid possible decomposition?

All fulvene compounds should be stored in freezer under Ar or N_2.

Question 3: Why is the fulvene compound the sole product instead of Sonogashira coupling products without the copper co-catalyst?

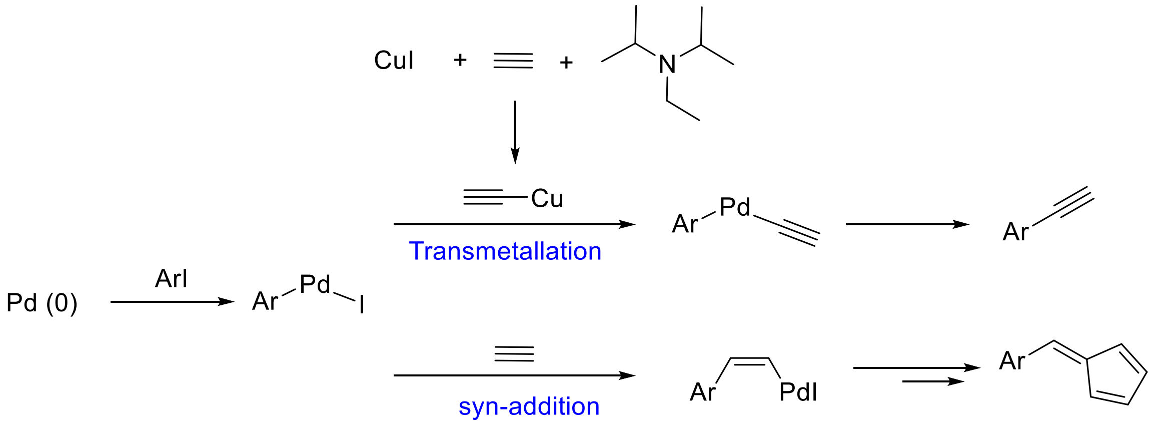

In the presence of copper catalyst and a base, acetylene will generate the acetylene copper complex, which is nucleophilic enough to undergo transmetallation with the aryl palladium intermediate to achieve the Sonogashira coupling products. Without the copper catalyst, the acetylene is not nucleophilic enough and *syn* addition will take place, which will eventually lead to fulvene products.

Question 4: Why is a packed-bed reactor used in the continuous-flow synthesis instead of a tubing reactor?

The outlet pressure of acetylene is < 30 psi. When we conducted the fulvene synthesis in continuous micro-tubing reactors with a back pressure regulator (5 psi) at 120 ^{o}C, the pressure cumulation can be higher than 30 psi which resulted in back flow in the continuous-flow system. We anticipated that the pressure drop will be much smaller in a packed-bed reactor instead of a micro-tubing reactor. This was true in that a packed-bed reactor filled with stainless steel powder was able to overcome the back-flow problem in our studies.

9.4 Fluorinated styrene synthesis

Question 1: What are the by-products in this reaction?

The common byproduct from this photo-initiated fluorinated styrene synthesis was the debrominated fluorinated arene. If the conversion did not reach 100%, the unreacted aryl bromide starting material was detected in the crude mixture as well.

Question 2: How to purify the desired product?

The crude mixtures, which may contain the debrominated arene, the aryl bromide starting material and the styrene product, were difficult to be separated by silica-gel based chromatography purification, because most of these compounds were un-polar, and co-spotted on the silica TLC. However, they can be effectively separated by Recycling Preparative HPLC equipped with JAIGEL -2H columns (GPC, $CHCl_3$, as an eluent) from

Japan Analytical Industry Co.,Ltd. We also expect that the reverse-phase (C18-based) HPLC or chromatography should achieve pure product separation as well.

Question 3: What can these fluorinated styrene products be used for?

These fluorinated styrene products can be used in a wide range of applications in fluorinated polymer synthesis.

10. References and Notes:

[1] A. U. Meyer, T. Slanina, C.-J. Yao, B. König, *ACS Catal.* **2016**, *6*, 369.

[2] J. A. Kozak, J. Wu, X. Su, F. Simeon, T. A. Hatton, T. F. Jamison, *J. Am. Chem. Soc*. **2013**, *135*, 18497.

[3] A. C. Spivey, C. G. Manas, I. Mann, *Chem. Commun.* **2005**, 4426.

[4] G. Majetich, R. Hicks, *J. Microw. Power Electromagn. Energy* **1995**, *30*, 27.

[5] K. E. Harding, L. M. May, K. F. Dick, *J. Org. Chem.* **1975**, *40*, 1664.

[6] Z.-L. Shen, X.-P. Xu, S.-J. Ji, *J. Org. Chem.* **2010**, *75*, 1162.

[7] J. Li, R. Hua, *Chem. Eur. J.* **2011**, *17*, 8462.

[8] K. Voigtritter, S. Ghorai, B. H. Lipshutz, *J. Org. Chem.* **2011**, *76*, 4697.

[9] A. Klapars, S. L. Buchwald, *J. Am. Chem. Soc.* **2002**, *124*, 14844.

[10], Y.-S. Feng C.-Q. Xie, W.-L. Qiao, H.-J. Xu, *Org. Lett*. **2013**, *15*, 936.

[11] M. Ghaffarzadeh, M. Bolourtchian, Z. H. Fard, M. R. Halvagar, F. Mohsenzadeh, *Syn. Comm.* **2006**, *36*, 1973.

[12] M. Beshai, B. Dhudshia, R. Mills, A. N. Thadani, *Tetrahedron Lett.* **2008**, *49*, 6794.

[13] T. Yasukawa, H. Miyamura, S. Kobayashi, *Org. Biomol. Chem.* **2011**, ***9***, 6208.

[14] M. A. Reddy, A. Thomas, G. Mallesham, B. Sridhar, V. J. Rao, K. Bhanuprakash, *Tetrahedron Lett.* **2011**, *52*, 6942.

[15] K. Kodaira,, K. Okuhara *Bull. Chem. Soc. Jpn.* **1988**, *61*, 1625.

[16] H. C. Bertrand, M. Schaap, L. Baird, N. D. Georgakopoulos, A. Fowkes, C. Thiollier, H. Kachi, A. T. Dinkova-Kostova, G. Wells, *J. Med. Chem.* **2015**, *58*, 7186.

[17] H. Liu, C. Chen, L. Wang, X. Tong, *Org. Lett.* **2011**, *13*, 5072.

[18] N. T. Patil, V. Singh, *Chem. Comm.* **2011**, *47*, 11116.

[19] N. Coçkun, I. Erden, *Tetrahedron* **2011**, *67*, 8607.

[20] M. Cini, T. D. Bradshaw, W. Lewis, S. Woodward, *Eur. J. Org. Chem.* **2013**, 3997.

[21] H. Müller-Bunz *et al. Zeitschrift fuer Kristallographie*, **2007**, *222*, 376.

[22] F.-J. Rehmann, L. P. Cuffe, O. Mendoza, D. K. Rai, N. Sweeney, K. Strohfeldt, W. M. Gallagher, M. Tacke, *Appl. Organometal. Chem.* **2005**, *19*, 293.

[23] N. Carrera, E. Gutiérrez, R. Benavente, M. M. Villavieja, Ana C. Albéniz, P. Espinet, *Chem. Eur. J.* **2008**, *14*, 10141.

[24] T. Braun, J. Izundu, A. Steffen, B. Neumann, H.-G. Stammler, *Dalton Trans*. **2006**, 5118

[25] K. Yuhsuke, K. Hiroo, A. Toshiki, Y. Yoshihiro, H. Hirofumi, Y. Yuya, *Polymer J.* **1985**, 1159.

[26] H. Wakami, J.-I. Yoshida, *Org. Process Res. Dev.* **2005**, *9*, 781-791.

[27] https://www.globalfia.com/store/view/productdetails/virtuemart_product_id/91/virtuemart_category_id/13

[28] Z. Novák, P. Nemes, A. Kotschy, *Org. Lett.* **2004**, *6*, 4917.

11. NMR Spectra for New Compounds

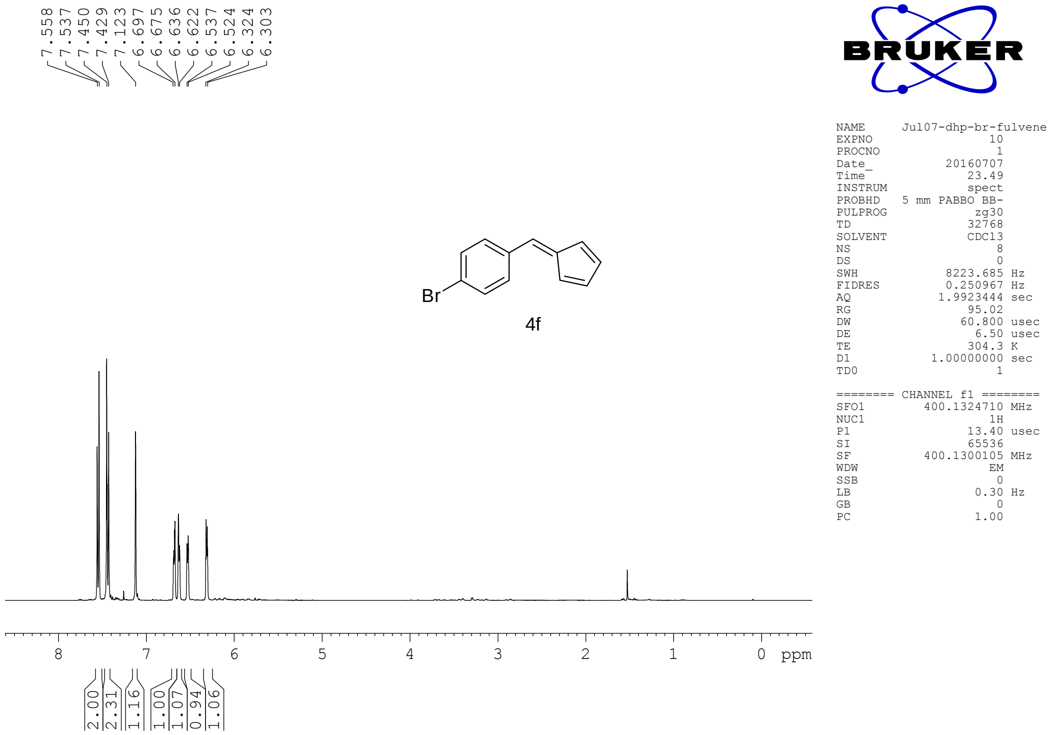

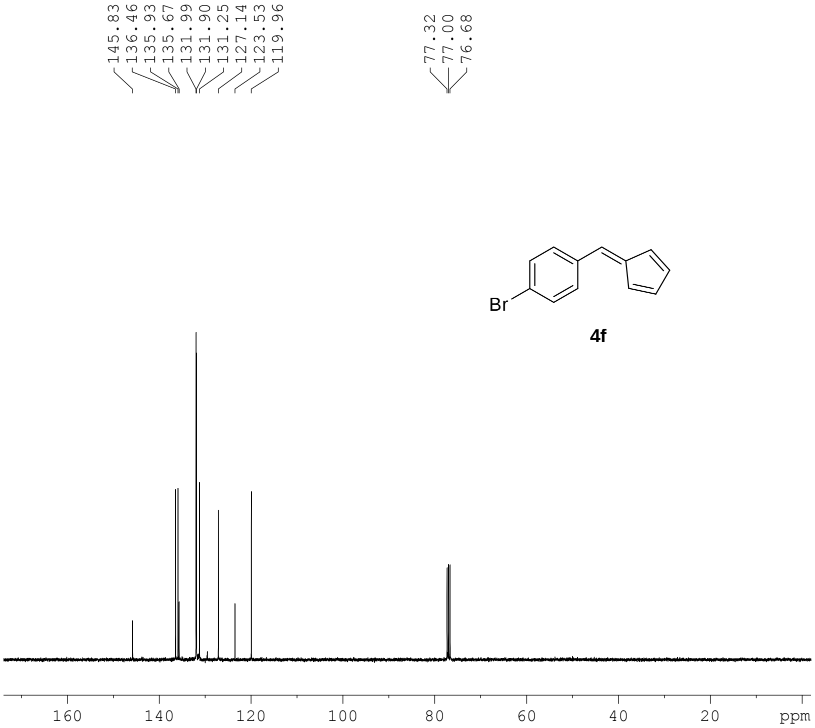


```
NAME       Jul07-dhp-br-fulvene
EXPNO                        11
PROCNO                        1
Date_                  20160708
Time                       0.14
INSTRUM                   spect
PROBHD     5 mm PABBO BB-
PULPROG                 zgpg30
TD                        65536
SOLVENT                   CDCl3
NS                          500
DS                            4
SWH                  24038.461 Hz
FIDRES                0.366798 Hz
AQ                   1.3631988 sec
RG                       194.02
DW                       20.800 usec
DE                         6.50 usec
TE                        305.1 K
D1                   1.50000000 sec
D11                  0.03000000 sec
TD0                           1

======== CHANNEL f1 ========
SFO1               100.6238364 MHz
NUC1                        13C
P1                         9.40 usec
SI                        32768
SF                 100.6127707 MHz
WDW                          EM
SSB                           0
LB                         1.00 Hz
GB                            0
PC                         1.40
```

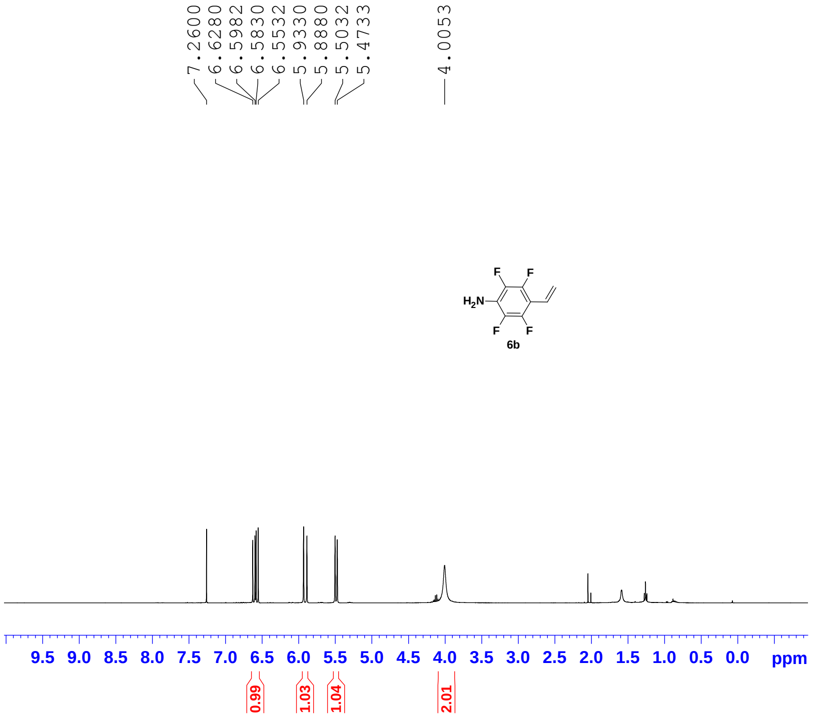


```
NAME       Aug08xf-NH2-GPC
EXPNO                        1
PROCNO                       1
Date_                 20160808
Time                     10.19
INSTRUM                  spect
PROBHD     5 mm PABBO BB-
PULPROG                   zg30
TD                       32768
SOLVENT                  CDCl3
NS                           8
DS                           0
SWH                   8223.685 Hz
FIDRES                0.250967 Hz
AQ                   1.9923444 sec
RG                      194.02
DW                      60.800 usec
DE                        6.50 usec
TE                       298.8 K
D1                  1.00000000 sec
TD0                          1

======== CHANNEL f1 ========
SFO1               400.1324710 MHz
NUC1                        1H
P1                        13.40 usec
SI                        65536
SF                 400.1300100 MHz
WDW                          EM
SSB                           0
LB                         0.30 Hz
GB                            0
PC                         1.00
```

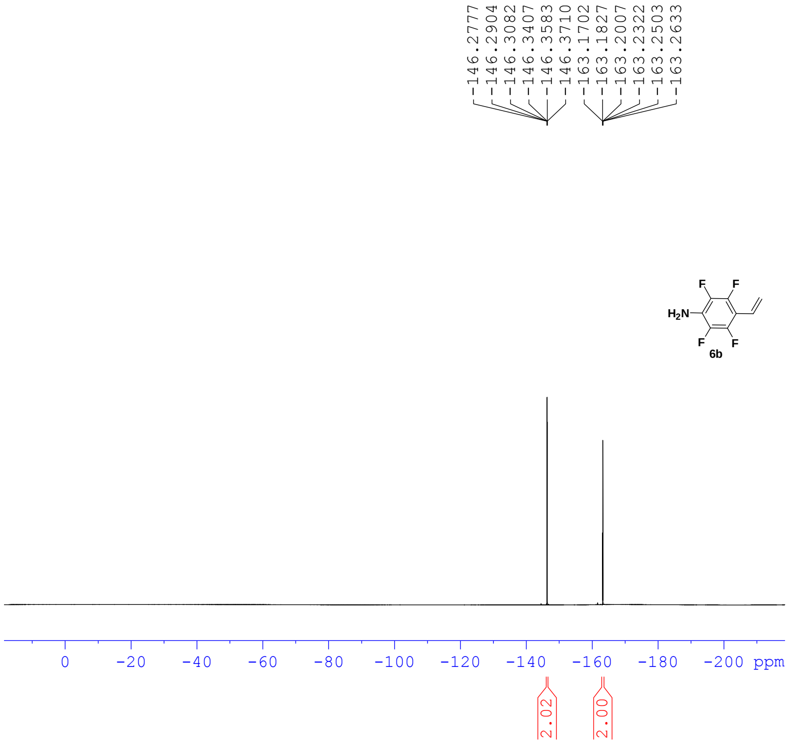

NAME Aug08xf-NH2-GPC
EXPNO 2
PROCNO 1
Date_ 20160808
Time 10.22
INSTRUM spect
PROBHD 5 mm PABBO BB-
PULPROG zgfhigqn.2
TD 131072
SOLVENT CDCl3
NS 32
DS 4
SWH 89285.711 Hz
FIDRES 0.681196 Hz
AQ 0.7340532 sec
RG 194.02
DW 5.600 usec
DE 6.50 usec
TE 299.1 K
D1 1.00000000 sec
D11 0.03000000 sec
D12 0.00002000 sec
TD0 1

======== CHANNEL f1 ========
SFO1 376.4607164 MHz
NUC1 19F
P1 13.40 usec
SI 65536
SF 376.4983660 MHz
WDW EM
SSB 0
LB 0.30 Hz
GB 0
PC 1.00

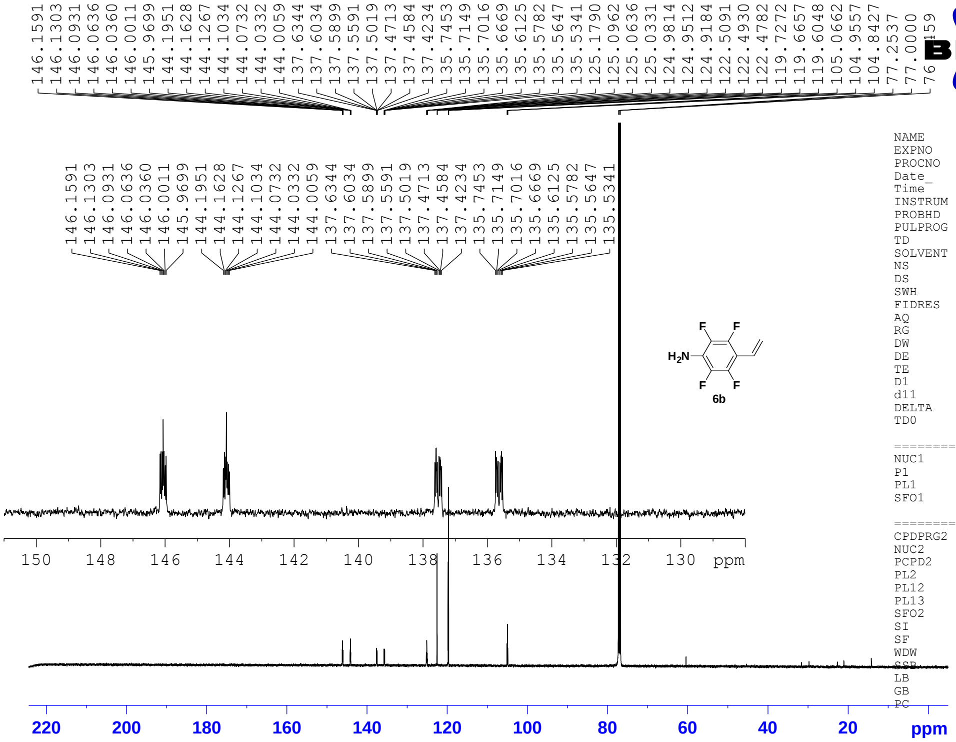

NAME xf0803-NH2-GPC
EXPNO 2
PROCNO 1
Date_ 20160803
Time 20.57
INSTRUM spect
PROBHD 5 mm PABBO BB/
PULPROG zgpg30
TD 65536
SOLVENT CDCl3
NS 13720
DS 0
SWH 30030.029 Hz
FIDRES 0.458222 Hz
AQ 1.0912410 sec
RG 16384
DW 16.650 usec
DE 6.00 usec
TE 295.7 K
D1 2.00000000 sec
d11 0.03000000 sec
DELTA 1.89999998 sec
TD0 1000

======== CHANNEL f1 ========
NUC1 13C
P1 8.90 usec
PL1 0.00 dB
SFO1 125.7709936 MHz

======== CHANNEL f2 ========
CPDPRG2 waltz16
NUC2 1H
PCPD2 80.00 usec
PL2 0.25 dB
PL12 17.89 dB
PL13 15.83 dB
SFO2 500.1320005 MHz
SI 32768
SF 125.7577903 MHz
WDW EM
SSB 0
LB 1.00 Hz
GB 0
PC 0.50

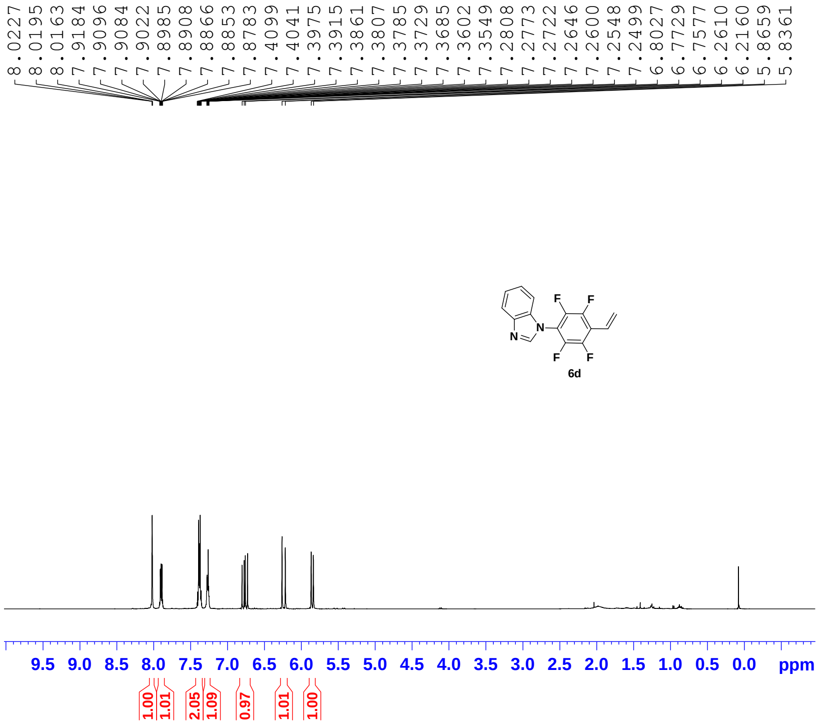

6d

NAME	Aug01xf-benimi-GPC
EXPNO	1
PROCNO	1
Date_	20160801
Time	11.52
INSTRUM	spect
PROBHD	5 mm PABBO BB-
PULPROG	zg30
TD	32768
SOLVENT	CDCl3
NS	8
DS	0
SWH	8223.685 Hz
FIDRES	0.250967 Hz
AQ	1.9923444 sec
RG	120.14
DW	60.800 usec
DE	6.50 usec
TE	299.3 K
D1	1.00000000 sec
TD0	1

======== CHANNEL f1 ========

SFO1	400.1324710 MHz
NUC1	1H
P1	13.40 usec
SI	65536
SF	400.1300100 MHz
WDW	EM
SSB	0
LB	0.30 Hz
GB	0
PC	1.00

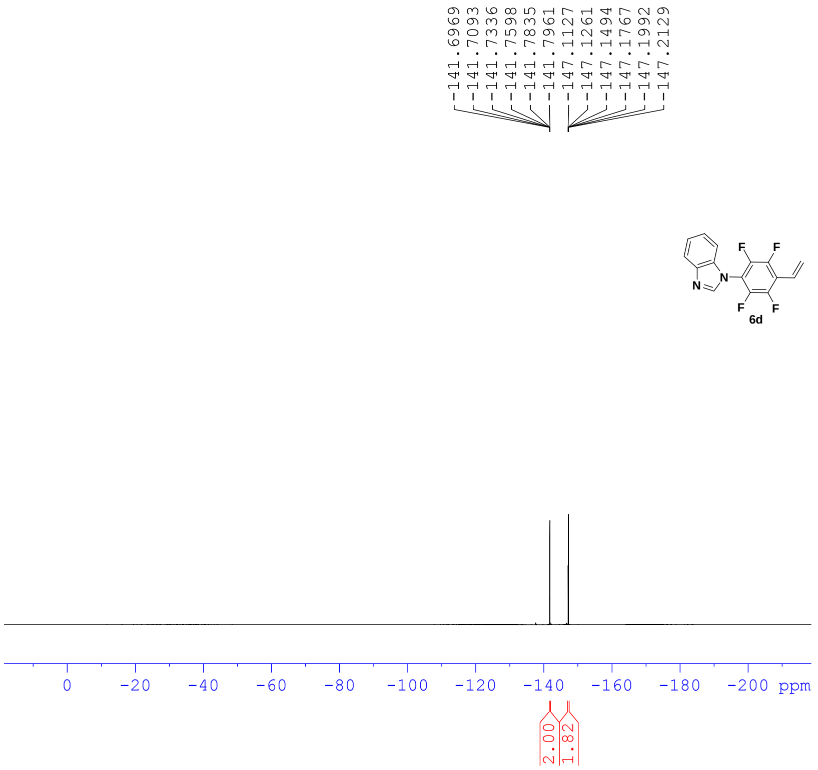

6d

NAME	Aug01xf-benimi-GPC
EXPNO	2
PROCNO	1
Date_	20160801
Time	11.54
INSTRUM	spect
PROBHD	5 mm PABBO BB-
PULPROG	zgfhigqn.2
TD	131072
SOLVENT	CDCl3
NS	32
DS	4
SWH	89285.711 Hz
FIDRES	0.681196 Hz
AQ	0.7340532 sec
RG	194.02
DW	5.600 usec
DE	6.50 usec
TE	299.6 K
D1	1.00000000 sec
D11	0.03000000 sec
D12	0.00002000 sec
TD0	1

======== CHANNEL f1 ========

SFO1	376.4607164 MHz
NUC1	19F
P1	13.40 usec
SI	65536
SF	376.4983660 MHz
WDW	EM
SSB	0
LB	0.30 Hz
GB	0
PC	1.00

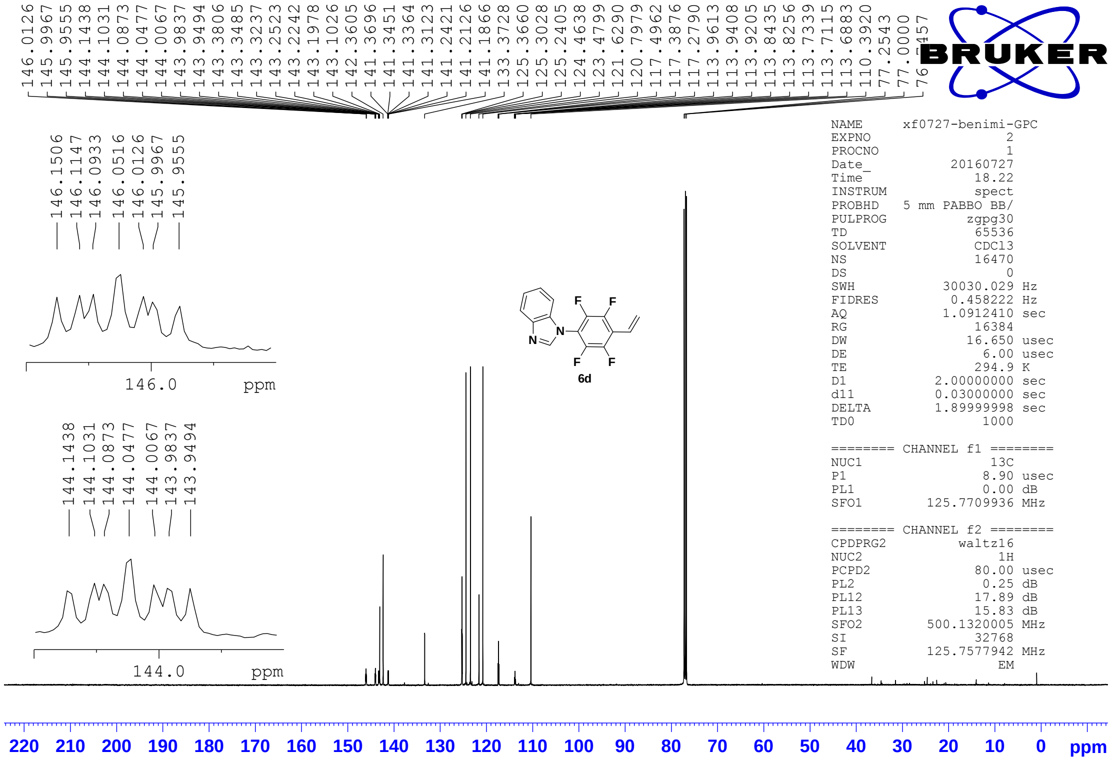
146.0126
145.9967
145.9555
144.1438
144.1031
144.0873
144.0477
144.0067
143.9837
143.9494
143.3806
143.3485
143.3237
143.2523
143.2242
143.1978
143.1026
142.3605
141.3696
141.3451
141.3364
141.3123
141.2421
141.2126
141.1866
133.3728
125.3660
125.3028
125.2405
124.4638
123.4799
121.6290
120.7979
117.4962
117.3876
117.2790
113.9613
113.9408
113.9205
113.8435
113.8256
113.7339
113.7115
113.6883
110.3920
77.2543
77.0000
BRUKER
146.1506
146.1147
146.0933
146.0516
146.0126
145.9967
145.9555
146.0
ppm
144.1438
144.1031
144.0873
144.0477
144.0067
143.9837
143.9494
144.0
ppm
F
F
N
N
F
F
6d
NAME xf0727-benimi-GPC
EXPNO 2
PROCNO 1
Date_ 20160727
Time 18.22
INSTRUM spect
PROBHD 5 mm PABBO BB/
PULPROG zgpg30
TD 65536
SOLVENT CDCl3
NS 16470
DS 0
SWH 30030.029 Hz
FIDRES 0.458222 Hz
AQ 1.0912410 sec
RG 16384
DW 16.650 usec
DE 6.00 usec
TE 294.9 K
D1 2.00000000 sec
d11 0.03000000 sec
DELTA 1.89999998 sec
TD0 1000
======== CHANNEL f1 ========
NUC1 13C
P1 8.90 usec
PL1 0.00 dB
SFO1 125.7709936 MHz
======== CHANNEL f2 ========
CPDPRG2 waltz16
NUC2 1H
PCPD2 80.00 usec
PL2 0.25 dB
PL12 17.89 dB
PL13 15.83 dB
SFO2 500.1320005 MHz
SI 32768
SF 125.7577942 MHz
WDW EM
220 210 200 190 180 170 160 150 140 130 120 110 100 90 80 70 60 50 40 30 20 10 0 ppm

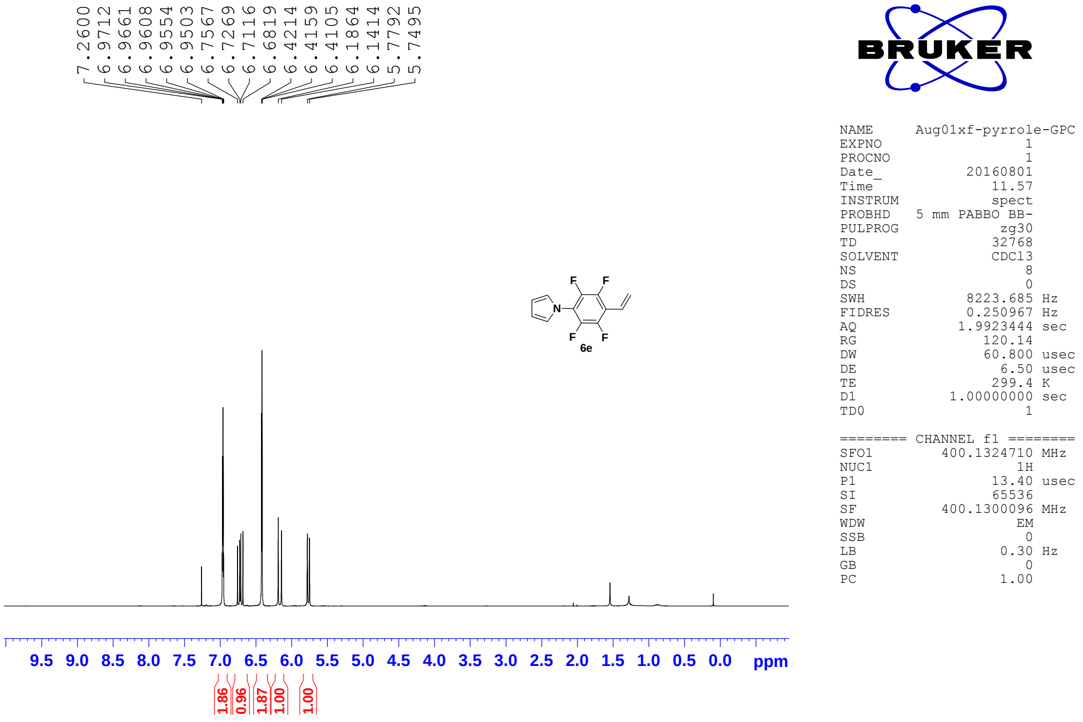
7.2600
6.9712
6.9661
6.9608
6.9554
6.9503
6.7567
6.7269
6.7116
6.6819
6.4214
6.4159
6.4105
6.1864
6.1414
5.7792
5.7495
BRUKER
F
F
N
F
F
6e
NAME Aug01xf-pyrrole-GPC
EXPNO 1
PROCNO 1
Date_ 20160801
Time 11.57
INSTRUM spect
PROBHD 5 mm PABBO BB-
PULPROG zg30
TD 32768
SOLVENT CDCl3
NS 8
DS 0
SWH 8223.685 Hz
FIDRES 0.250967 Hz
AQ 1.9923444 sec
RG 120.14
DW 60.800 usec
DE 6.50 usec
TE 299.4 K
D1 1.00000000 sec
TD0 1
======== CHANNEL f1 ========
SFO1 400.1324710 MHz
NUC1 1H
P1 13.40 usec
SI 65536
SF 400.1300096 MHz
WDW EM
SSB 0
LB 0.30 Hz
GB 0
PC 1.00
9.5 9.0 8.5 8.0 7.5 7.0 6.5 6.0 5.5 5.0 4.5 4.0 3.5 3.0 2.5 2.0 1.5 1.0 0.5 0.0 ppm
1.86
0.96
1.87
1.00
1.00

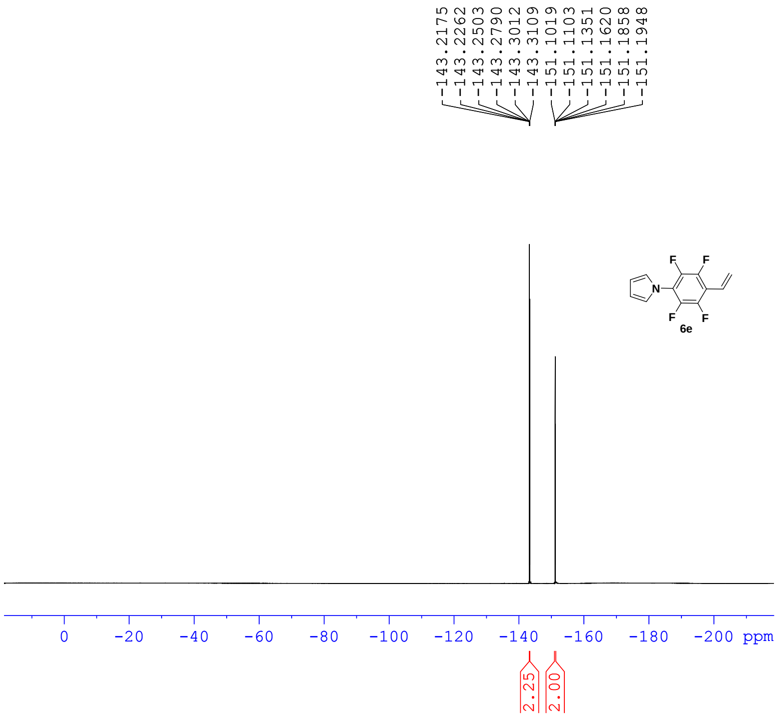

BRUKER

NAME	Aug01xf-pyrrole-GPC
EXPNO	2
PROCNO	1
Date_	20160801
Time	11.58
INSTRUM	spect
PROBHD	5 mm PABBO BB-
PULPROG	zgfhigqn.2
TD	131072
SOLVENT	CDCl3
NS	32
DS	4
SWH	89285.711 Hz
FIDRES	0.681196 Hz
AQ	0.7340532 sec
RG	194.02
DW	5.600 usec
DE	6.50 usec
TE	299.7 K
D1	1.00000000 sec
D11	0.03000000 sec
D12	0.00002000 sec
TD0	1

======== CHANNEL f1 ========

SFO1	376.4607164 MHz
NUC1	19F
P1	13.40 usec
SI	65536
SF	376.4983660 MHz
WDW	EM
SSB	0
LB	0.30 Hz
GB	0
PC	1.00

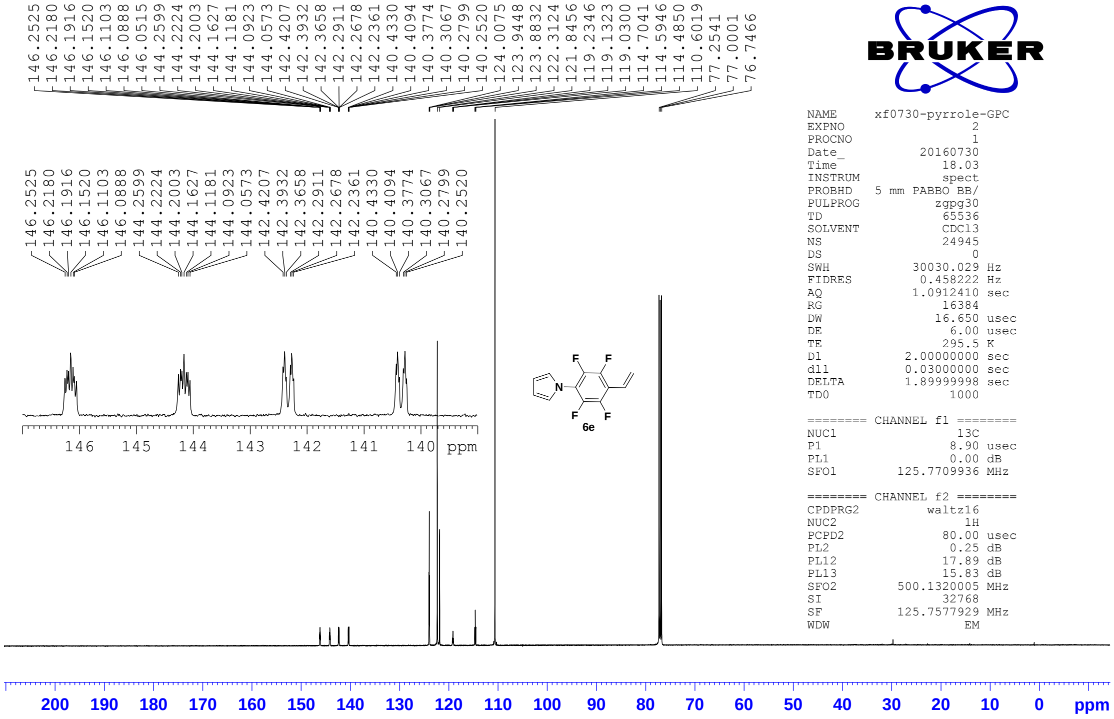

BRUKER

NAME	xf0730-pyrrole-GPC
EXPNO	2
PROCNO	1
Date_	20160730
Time	18.03
INSTRUM	spect
PROBHD	5 mm PABBO BB/
PULPROG	zgpg30
TD	65536
SOLVENT	CDCl3
NS	24945
DS	0
SWH	30030.029 Hz
FIDRES	0.458222 Hz
AQ	1.0912410 sec
RG	16384
DW	16.650 usec
DE	6.00 usec
TE	295.5 K
D1	2.00000000 sec
d11	0.03000000 sec
DELTA	1.89999998 sec
TD0	1000

======== CHANNEL f1 ========

NUC1	13C
P1	8.90 usec
PL1	0.00 dB
SFO1	125.7709936 MHz

======== CHANNEL f2 ========

CPDPRG2	waltz16
NUC2	1H
PCPD2	80.00 usec
PL2	0.25 dB
PL12	17.89 dB
PL13	15.83 dB
SFO2	500.1320005 MHz
SI	32768
SF	125.7577929 MHz
WDW	EM

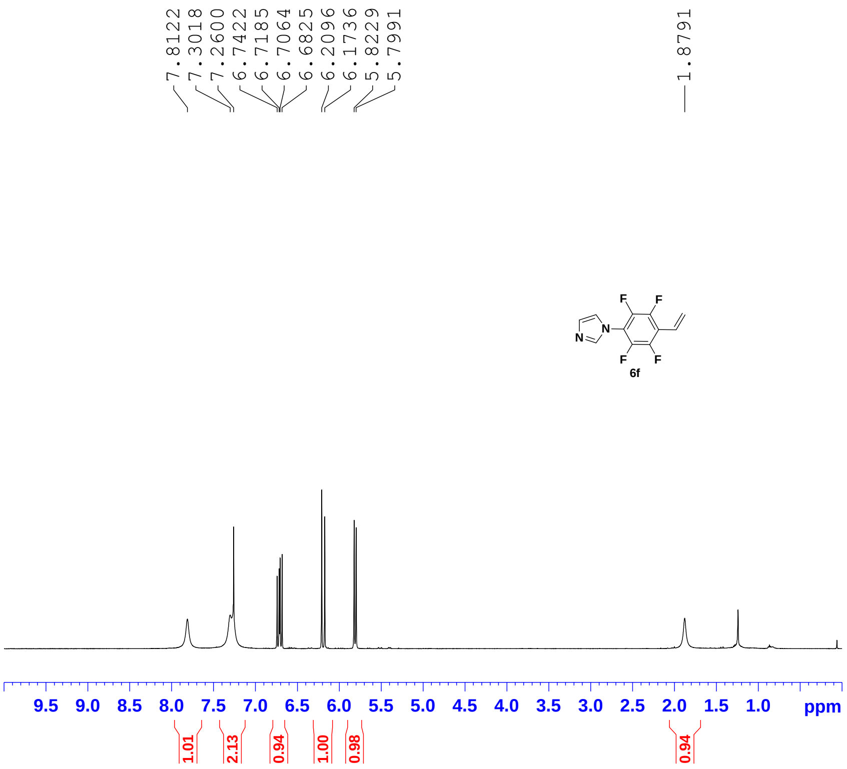


```
NAME      xf0725-imi-GPC
EXPNO                  1
PROCNO                 1
Date_           20160725
Time               18.03
INSTRUM            spect
PROBHD   5 mm PABBO BB/
PULPROG             zg30
TD                 32768
SOLVENT            CDCl3
NS                     8
DS                     0
SWH            10330.578 Hz
FIDRES          0.315264 Hz
AQ             1.5860696 sec
RG                 228.1
DW                48.400 usec
DE                  6.00 usec
TE                 292.5 K
D1            1.00000000 sec
TD0                    1

======== CHANNEL f1 ========
NUC1                  1H
P1                 10.50 usec
PL1                 0.25 dB
SFO1         500.1330885 MHz
SI                 16384
SF           500.1300135 MHz
WDW                   EM
SSB                    0
LB                  0.30 Hz
GB                     0
PC                  1.00
```

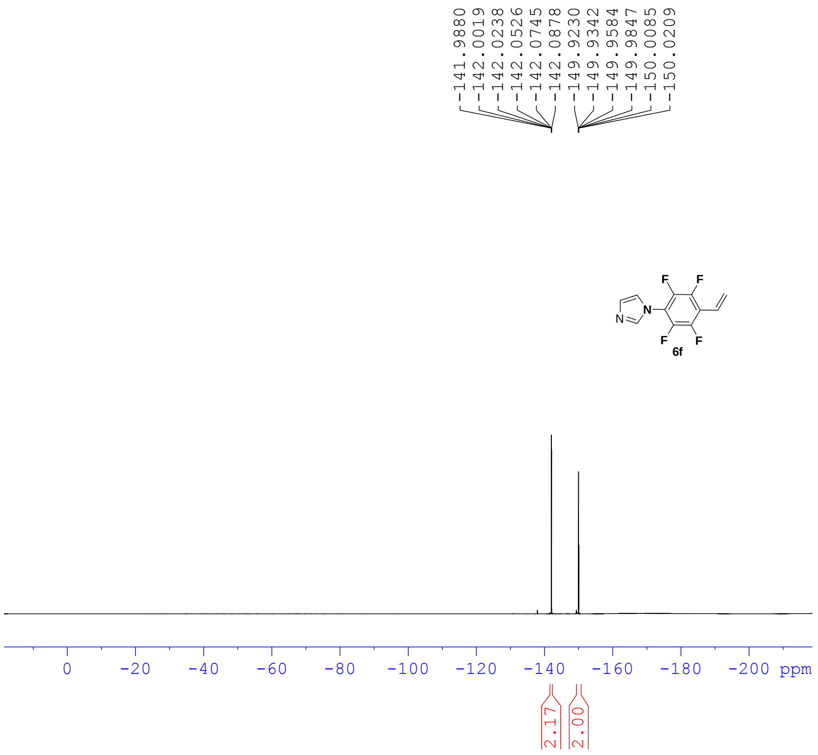


```
NAME      Aug03xf-imi-GPC
EXPNO                  2
PROCNO                 1
Date_           20160804
Time                5.54
INSTRUM            spect
PROBHD   5 mm PABBO BB-
PULPROG       zgfhigqn.2
TD                131072
SOLVENT            CDCl3
NS                    32
DS                     4
SWH            89285.711 Hz
FIDRES          0.681196 Hz
AQ             0.7340532 sec
RG                194.02
DW                 5.600 usec
DE                  6.50 usec
TE                 299.0 K
D1            1.00000000 sec
D11           0.03000000 sec
D12           0.00002000 sec
TD0                    1

======== CHANNEL f1 ========
SFO1         376.4607164 MHz
NUC1                 19F
P1                 13.40 usec
SI                 65536
SF           376.4983660 MHz
WDW                   EM
SSB                    0
LB                  0.30 Hz
GB                     0
PC                  1.00
```

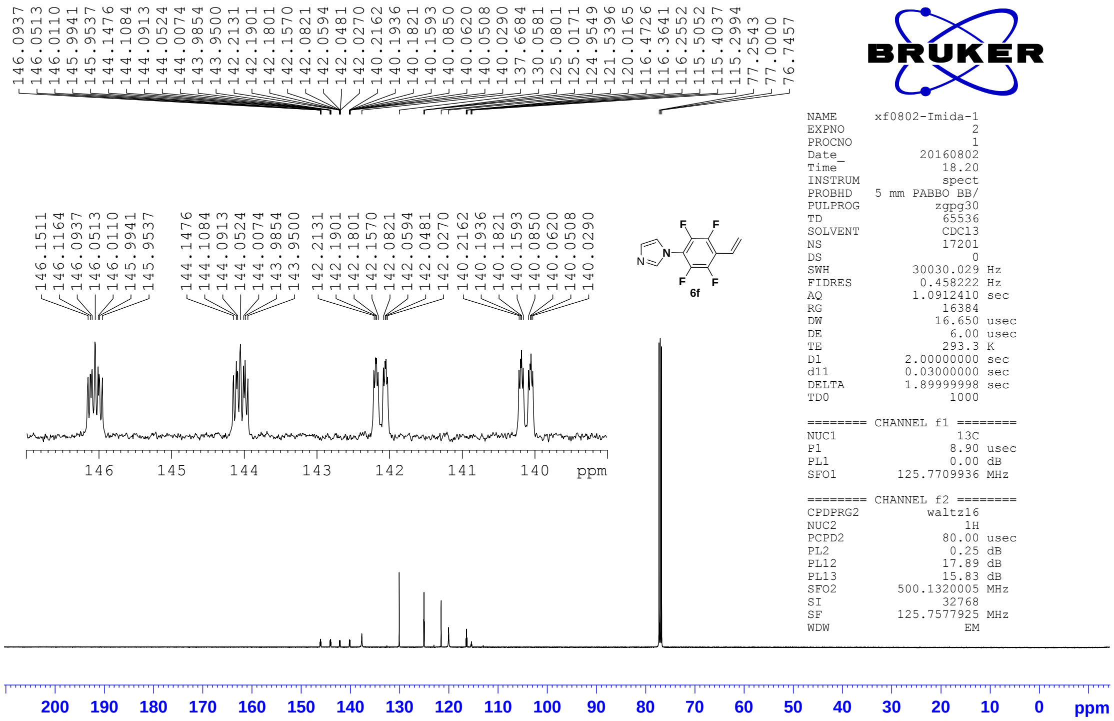
146.0937
146.0513
146.0110
145.9941
145.9537
144.1476
144.1084
144.0913
144.0524
144.0074
143.9854
143.9500
142.2131
142.1901
142.1801
142.1570
142.0821
142.0594
142.0481
142.0270
140.2162
140.1936
140.1821
140.1593
140.0850
140.0620
140.0508
140.0290
137.6684
130.0581
125.0801
125.0171
124.9549
121.5396
120.0165
116.4726
116.3641
116.2552
115.5052
115.4037
115.2994
77.2543
77.0000
76.7457
146.1511
146.1164
146.0937
146.0513
146.0110
145.9941
145.9537
144.1476
144.1084
144.0913
144.0524
144.0074
143.9854
143.9500
142.2131
142.1901
142.1801
142.1570
142.0821
142.0594
142.0481
142.0270
140.2162
140.1936
140.1821
140.1593
140.0850
140.0620
140.0508
140.0290
146 145 144 143 142 141 140 ppm
6f
BRUKER
NAME xf0802-Imida-1
EXPNO 2
PROCNO 1
Date_ 20160802
Time 18.20
INSTRUM spect
PROBHD 5 mm PABBO BB/
PULPROG zgpg30
TD 65536
SOLVENT CDCl3
NS 17201
DS 0
SWH 30030.029 Hz
FIDRES 0.458222 Hz
AQ 1.0912410 sec
RG 16384
DW 16.650 usec
DE 6.00 usec
TE 293.3 K
D1 2.00000000 sec
d11 0.03000000 sec
DELTA 1.89999998 sec
TD0 1000
======== CHANNEL f1 ========
NUC1 13C
P1 8.90 usec
PL1 0.00 dB
SFO1 125.7709936 MHz
======== CHANNEL f2 ========
CPDPRG2 waltz16
NUC2 1H
PCPD2 80.00 usec
PL2 0.25 dB
PL12 17.89 dB
PL13 15.83 dB
SFO2 500.1320005 MHz
SI 32768
SF 125.7577925 MHz
WDW EM
200 190 180 170 160 150 140 130 120 110 100 90 80 70 60 50 40 30 20 10 0 ppm

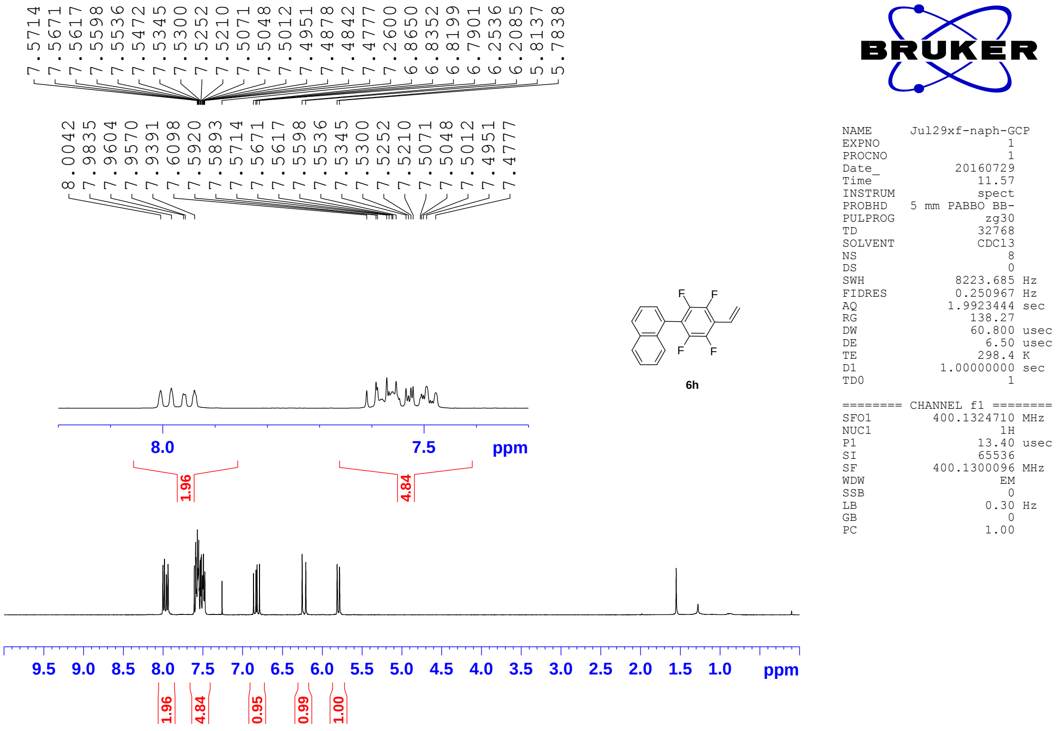
7.5714
7.5671
7.5617
7.5598
7.5536
7.5472
7.5345
7.5300
7.5252
7.5210
7.5071
7.5048
7.5012
7.4951
7.4878
7.4842
7.4777
7.2600
6.8650
6.8352
6.8199
6.7901
6.2536
6.2085
5.8137
5.7838
8.0042
7.9835
7.9604
7.9570
7.9391
7.6098
7.5920
7.5893
7.5714
7.5671
7.5617
7.5598
7.5536
7.5345
7.5300
7.5252
7.5210
7.5071
7.5048
7.5012
7.4951
7.4777
6h
8.0 7.5 ppm
1.96
4.84
BRUKER
NAME Jul29xf-naph-GCP
EXPNO 1
PROCNO 1
Date_ 20160729
Time 11.57
INSTRUM spect
PROBHD 5 mm PABBO BB-
PULPROG zg30
TD 32768
SOLVENT CDCl3
NS 8
DS 0
SWH 8223.685 Hz
FIDRES 0.250967 Hz
AQ 1.9923444 sec
RG 138.27
DW 60.800 usec
DE 6.50 usec
TE 298.4 K
D1 1.00000000 sec
TD0 1
======== CHANNEL f1 ========
SFO1 400.1324710 MHz
NUC1 1H
P1 13.40 usec
SI 65536
SF 400.1300096 MHz
WDW EM
SSB 0
LB 0.30 Hz
GB 0
PC 1.00
9.5 9.0 8.5 8.0 7.5 7.0 6.5 6.0 5.5 5.0 4.5 4.0 3.5 3.0 2.5 2.0 1.5 1.0 ppm
1.96
4.84
0.95
0.99
1.00

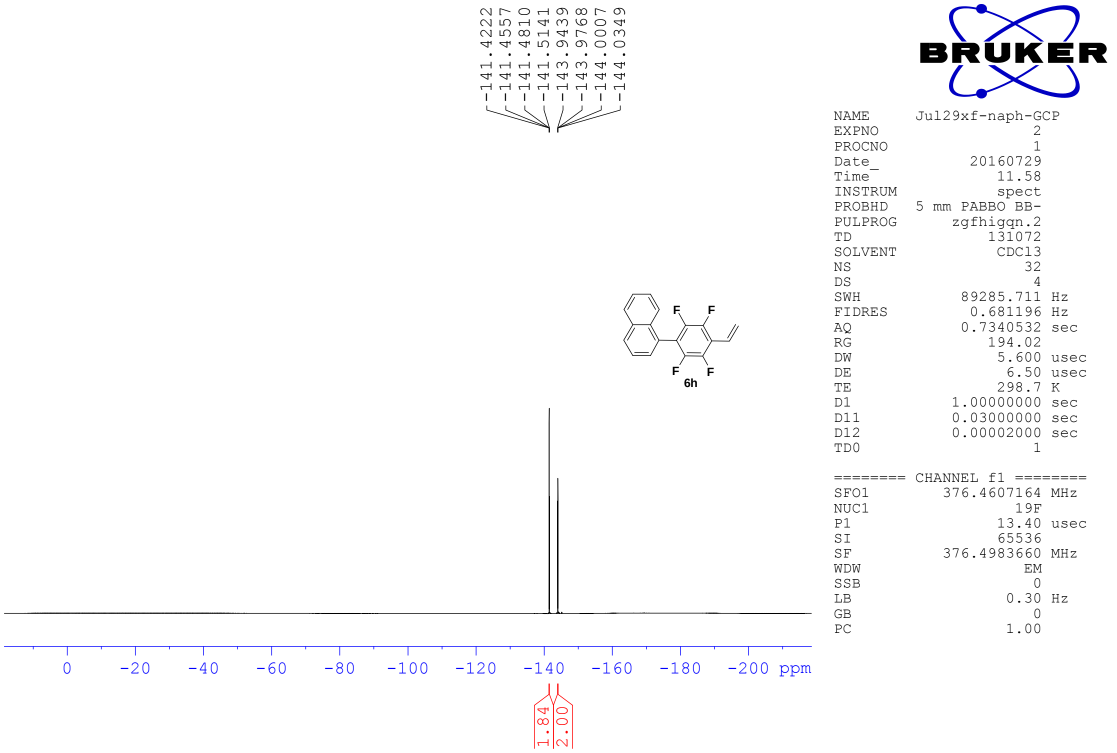

-141.4222
-141.4557
-141.4810
-141.5141
-143.9439
-143.9768
-144.0007
-144.0349
6h
NAME Jul29xf-naph-GCP
EXPNO 2
PROCNO 1
Date_ 20160729
Time 11.58
INSTRUM spect
PROBHD 5 mm PABBO BB-
PULPROG zgfhigqn.2
TD 131072
SOLVENT CDCl3
NS 32
DS 4
SWH 89285.711 Hz
FIDRES 0.681196 Hz
AQ 0.7340532 sec
RG 194.02
DW 5.600 usec
DE 6.50 usec
TE 298.7 K
D1 1.00000000 sec
D11 0.03000000 sec
D12 0.00002000 sec
TD0 1
======== CHANNEL f1 ========
SFO1 376.4607164 MHz
NUC1 19F
P1 13.40 usec
SI 65536
SF 376.4983660 MHz
WDW EM
SSB 0
LB 0.30 Hz
GB 0
PC 1.00
0 -20 -40 -60 -80 -100 -120 -140 -160 -180 -200 ppm
1.84
2.00

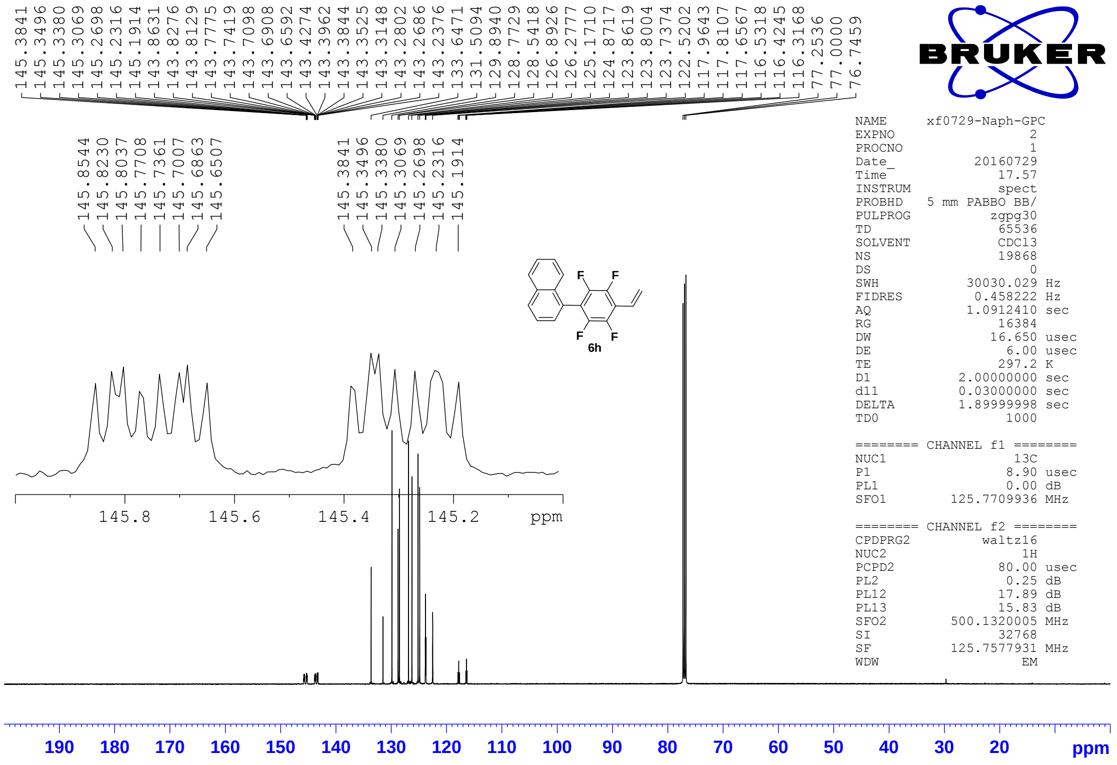

145.3841
145.3496
145.3380
145.3069
145.2698
145.2316
145.1914
143.8631
143.8276
143.8129
143.7775
143.7419
143.7098
143.6908
143.6592
143.4274
143.3962
143.3844
143.3525
143.3148
143.2802
143.2686
143.2376
133.6471
131.5094
129.8940
128.7729
128.5418
126.8926
126.2777
125.1710
124.8717
123.8619
123.8004
123.7374
122.5202
117.9643
117.8107
117.6567
116.5318
116.4245
116.3168
77.2536
77.0000
76.7459
145.8544
145.8230
145.8037
145.7708
145.7361
145.7007
145.6863
145.6507
145.3841
145.3496
145.3380
145.3069
145.2698
145.2316
145.1914
6h
145.8 145.6 145.4 145.2 ppm
NAME xf0729-Naph-GPC
EXPNO 2
PROCNO 1
Date_ 20160729
Time 17.57
INSTRUM spect
PROBHD 5 mm PABBO BB/
PULPROG zgpg30
TD 65536
SOLVENT CDCl3
NS 19868
DS 0
SWH 30030.029 Hz
FIDRES 0.458222 Hz
AQ 1.0912410 sec
RG 16384
DW 16.650 usec
DE 6.00 usec
TE 297.2 K
D1 2.00000000 sec
d11 0.03000000 sec
DELTA 1.89999998 sec
TD0 1000
======== CHANNEL f1 ========
NUC1 13C
P1 8.90 usec
PL1 0.00 dB
SFO1 125.7709936 MHz
======== CHANNEL f2 ========
CPDPRG2 waltz16
NUC2 1H
PCPD2 80.00 usec
PL2 0.25 dB
PL12 17.89 dB
PL13 15.83 dB
SFO2 500.1320005 MHz
SI 32768
SF 125.7577931 MHz
WDW EM
190 180 170 160 150 140 130 120 110 100 90 80 70 60 50 40 30 20 ppm

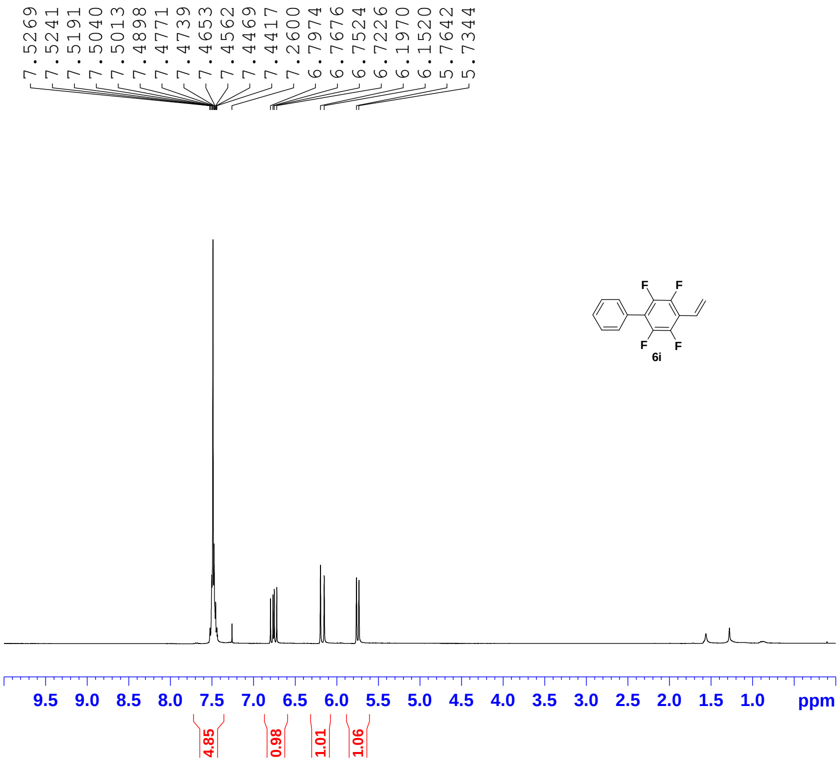


```
NAME      Aug03xf-ph-GPC
EXPNO                  1
PROCNO                 1
Date_           20160804
Time                5.45
INSTRUM            spect
PROBHD   5 mm PABBO BB-
PULPROG             zg30
TD                 32768
SOLVENT            CDCl3
NS                     8
DS                     0
SWH             8223.685 Hz
FIDRES          0.250967 Hz
AQ             1.9923444 sec
RG                 95.02
DW                60.800 usec
DE                  6.50 usec
TE                 298.9 K
D1            1.00000000 sec
TD0                    1

======== CHANNEL f1 ========
SFO1         400.1324710 MHz
NUC1                  1H
P1                 13.40 usec
SI                 65536
SF           400.1300099 MHz
WDW                   EM
SSB                    0
LB                  0.30 Hz
GB                     0
PC                  1.00
```

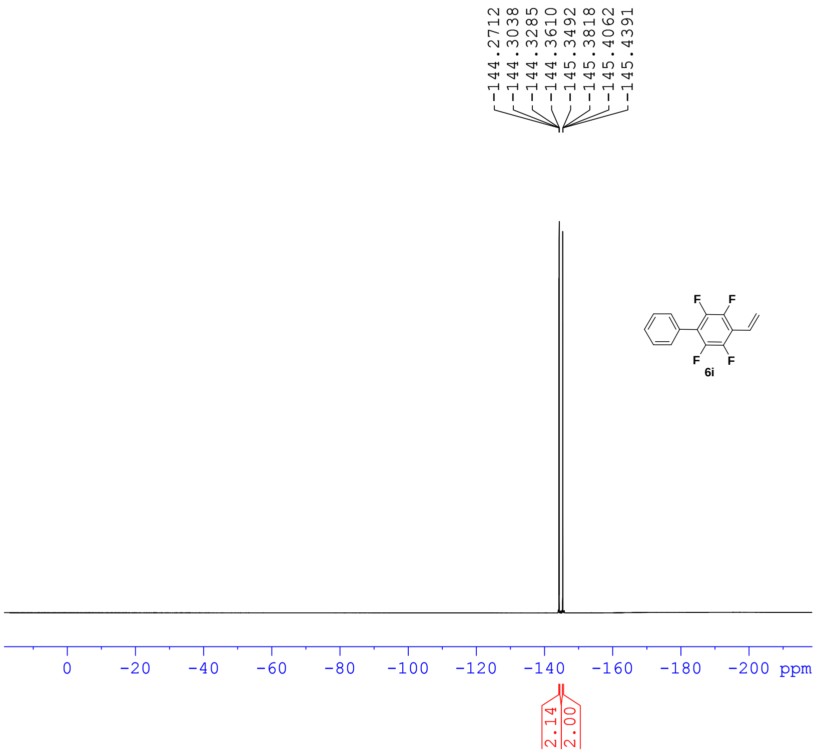


```
NAME      Aug03xf-ph-GPC
EXPNO                  2
PROCNO                 1
Date_           20160804
Time                5.48
INSTRUM            spect
PROBHD   5 mm PABBO BB-
PULPROG       zgfhigqn.2
TD                131072
SOLVENT            CDCl3
NS                    32
DS                     4
SWH            89285.711 Hz
FIDRES          0.681196 Hz
AQ             0.7340532 sec
RG                194.02
DW                 5.600 usec
DE                  6.50 usec
TE                 299.1 K
D1            1.00000000 sec
D11           0.03000000 sec
D12           0.00002000 sec
TD0                    1

======== CHANNEL f1 ========
SFO1         376.4607164 MHz
NUC1                 19F
P1                 13.40 usec
SI                 65536
SF           376.4983660 MHz
WDW                   EM
SSB                    0
LB                  0.30 Hz
GB                     0
PC                  1.00
```

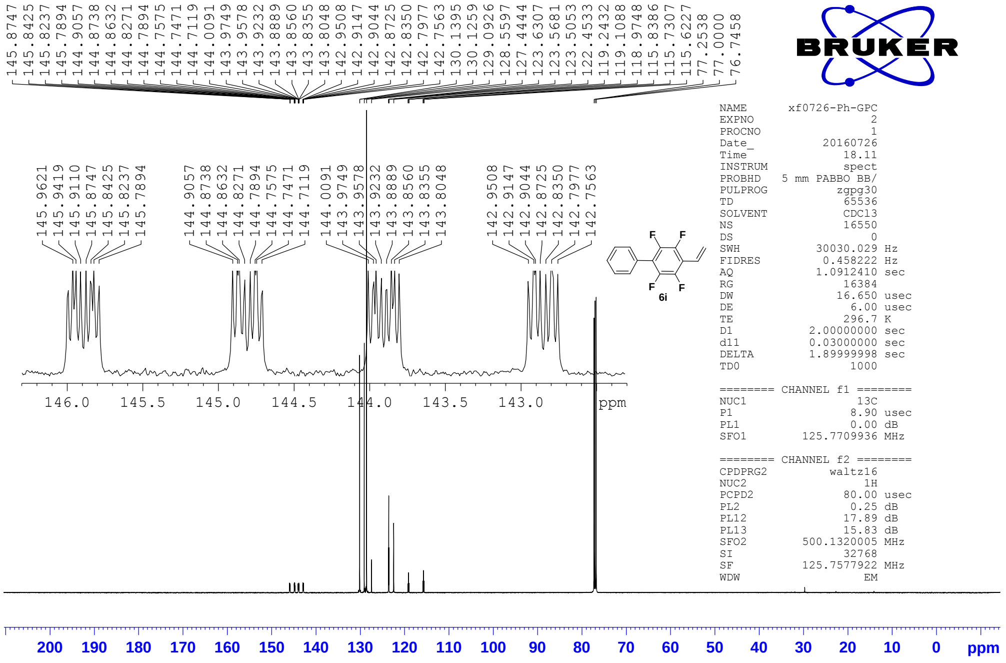
145.8747
145.8425
145.8237
145.7894
144.9057
144.8738
144.8632
144.8271
144.7894
144.7575
144.7471
144.7119
144.0091
143.9749
143.9578
143.9232
143.8889
143.8560
143.8355
143.8048
142.9508
142.9147
142.9044
142.8725
142.8350
142.7977
142.7563
130.1395
130.1259
129.0926
128.5597
127.4444
123.6307
123.5681
123.5053
122.4533
119.2432
119.1088
118.9748
115.8386
115.7307
115.6227
77.2538
77.0000
76.7458
145.9621
145.9419
145.9110
145.8747
145.8425
145.8237
145.7894
144.9057
144.8738
144.8632
144.8271
144.7894
144.7575
144.7471
144.7119
144.0091
143.9749
143.9578
143.9232
143.8889
143.8560
143.8355
143.8048
142.9508
142.9147
142.9044
142.8725
142.8350
142.7977
142.7563
146.0 145.5 145.0 144.5 144.0 143.5 143.0 ppm
6i
BRUKER
NAME xf0726-Ph-GPC
EXPNO 2
PROCNO 1
Date_ 20160726
Time 18.11
INSTRUM spect
PROBHD 5 mm PABBO BB/
PULPROG zgpg30
TD 65536
SOLVENT CDCl3
NS 16550
DS 0
SWH 30030.029 Hz
FIDRES 0.458222 Hz
AQ 1.0912410 sec
RG 16384
DW 16.650 usec
DE 6.00 usec
TE 296.7 K
D1 2.00000000 sec
d11 0.03000000 sec
DELTA 1.89999998 sec
TD0 1000
======== CHANNEL f1 ========
NUC1 13C
P1 8.90 usec
PL1 0.00 dB
SFO1 125.7709936 MHz
======== CHANNEL f2 ========
CPDPRG2 waltz16
NUC2 1H
PCPD2 80.00 usec
PL2 0.25 dB
PL12 17.89 dB
PL13 15.83 dB
SFO2 500.1320005 MHz
SI 32768
SF 125.7577922 MHz
WDW EM
200 190 180 170 160 150 140 130 120 110 100 90 80 70 60 50 40 30 20 10 0 ppm

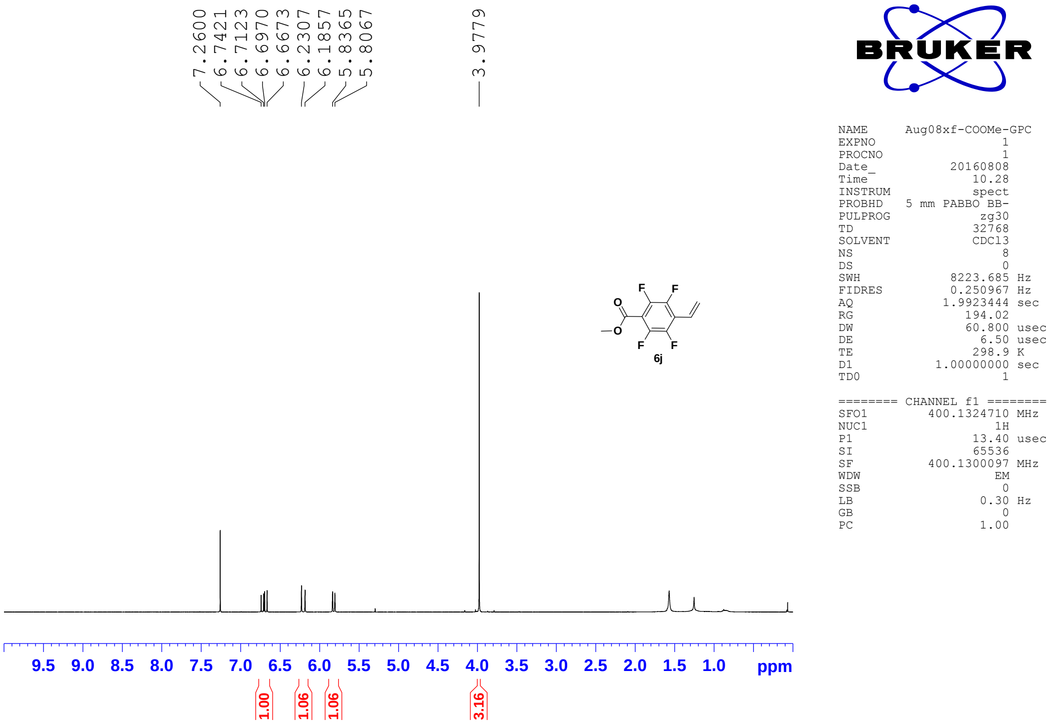
7.2600
6.7421
6.7123
6.6970
6.6673
6.2307
6.1857
5.8365
5.8067
3.9779
BRUKER
NAME Aug08xf-COOMe-GPC
EXPNO 1
PROCNO 1
Date_ 20160808
Time 10.28
INSTRUM spect
PROBHD 5 mm PABBO BB-
PULPROG zg30
TD 32768
SOLVENT CDCl3
NS 8
DS 0
SWH 8223.685 Hz
FIDRES 0.250967 Hz
AQ 1.9923444 sec
RG 194.02
DW 60.800 usec
DE 6.50 usec
TE 298.9 K
D1 1.00000000 sec
TD0 1
======== CHANNEL f1 ========
SFO1 400.1324710 MHz
NUC1 1H
P1 13.40 usec
SI 65536
SF 400.1300097 MHz
WDW EM
SSB 0
LB 0.30 Hz
GB 0
PC 1.00
6j
9.5 9.0 8.5 8.0 7.5 7.0 6.5 6.0 5.5 5.0 4.5 4.0 3.5 3.0 2.5 2.0 1.5 1.0 ppm
1.00
1.06
1.06
3.16

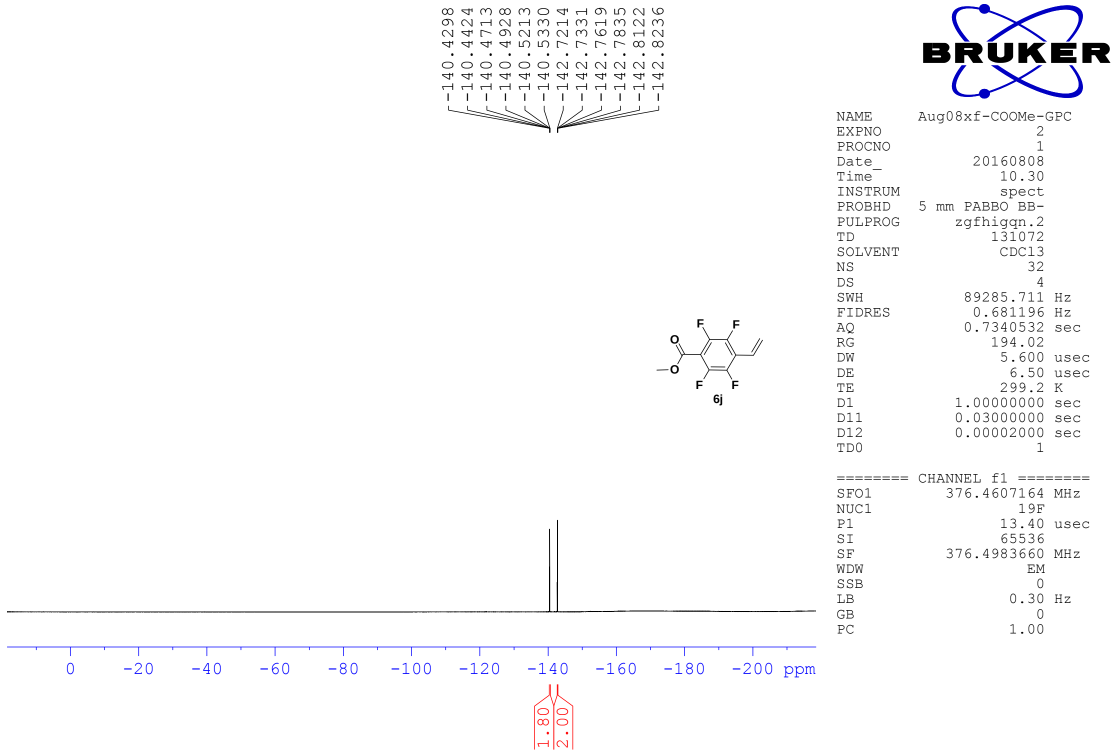
-140.4298
-140.4424
-140.4713
-140.4928
-140.5213
-140.5330
-142.7214
-142.7331
-142.7619
-142.7835
-142.8122
-142.8236
BRUKER
NAME Aug08xf-COOMe-GPC
EXPNO 2
PROCNO 1
Date_ 20160808
Time 10.30
INSTRUM spect
PROBHD 5 mm PABBO BB-
PULPROG zgfhigqn.2
TD 131072
SOLVENT CDCl3
NS 32
DS 4
SWH 89285.711 Hz
FIDRES 0.681196 Hz
AQ 0.7340532 sec
RG 194.02
DW 5.600 usec
DE 6.50 usec
TE 299.2 K
D1 1.00000000 sec
D11 0.03000000 sec
D12 0.00002000 sec
TD0 1
======== CHANNEL f1 ========
SFO1 376.4607164 MHz
NUC1 19F
P1 13.40 usec
SI 65536
SF 376.4983660 MHz
WDW EM
SSB 0
LB 0.30 Hz
GB 0
PC 1.00
6j
0 -20 -40 -60 -80 -100 -120 -140 -160 -180 -200 ppm
1.80
2.00

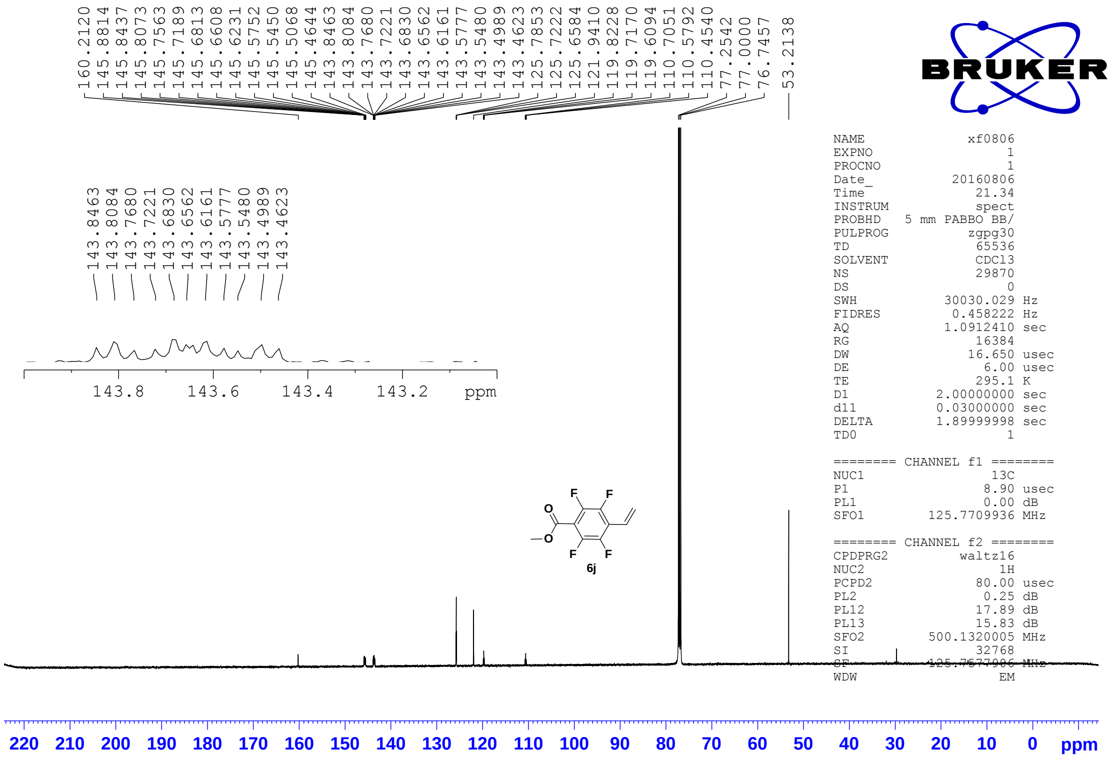
160.2120
145.8814
145.8437
145.8073
145.7563
145.7189
145.6813
145.6608
145.6231
145.5752
145.5450
145.5068
145.4644
143.8463
143.8084
143.7680
143.7221
143.6830
143.6562
143.6161
143.5777
143.5480
143.4989
143.4623
125.7853
125.7222
125.6584
121.9410
119.8228
119.7170
119.6094
110.7051
110.5792
110.4540
77.2542
77.0000
76.7457
53.2138
143.8 143.6 143.4 143.2 ppm
BRUKER
NAME xf0806
EXPNO 1
PROCNO 1
Date_ 20160806
Time 21.34
INSTRUM spect
PROBHD 5 mm PABBO BB/
PULPROG zgpg30
TD 65536
SOLVENT CDCl3
NS 29870
DS 0
SWH 30030.029 Hz
FIDRES 0.458222 Hz
AQ 1.0912410 sec
RG 16384
DW 16.650 usec
DE 6.00 usec
TE 295.1 K
D1 2.00000000 sec
d11 0.03000000 sec
DELTA 1.89999998 sec
TD0 1
======== CHANNEL f1 ========
NUC1 13C
P1 8.90 usec
PL1 0.00 dB
SFO1 125.7709936 MHz
======== CHANNEL f2 ========
CPDPRG2 waltz16
NUC2 1H
PCPD2 80.00 usec
PL2 0.25 dB
PL12 17.89 dB
PL13 15.83 dB
SFO2 500.1320005 MHz
SI 32768
SF 125.7577906 MHz
WDW EM
6j
220 210 200 190 180 170 160 150 140 130 120 110 100 90 80 70 60 50 40 30 20 10 0 ppm

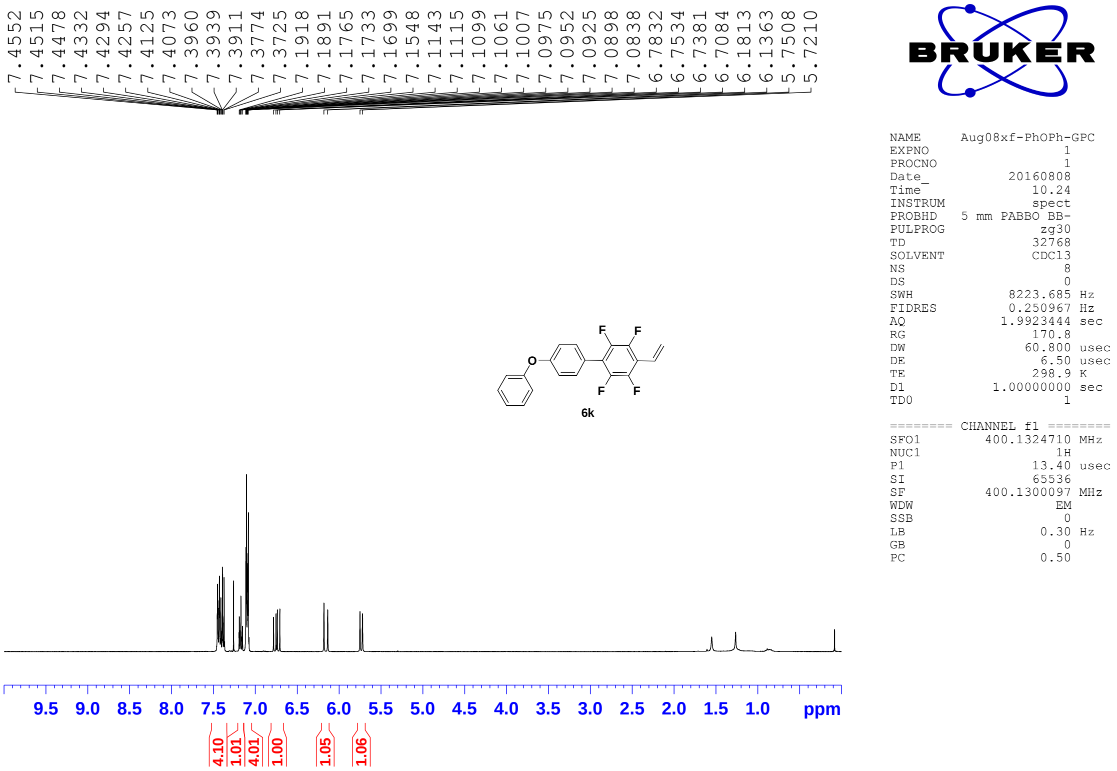

7.4552
7.4515
7.4478
7.4332
7.4294
7.4257
7.4125
7.4073
7.3960
7.3939
7.3911
7.3774
7.3725
7.1918
7.1891
7.1765
7.1733
7.1699
7.1548
7.1143
7.1115
7.1099
7.1061
7.1007
7.0975
7.0952
7.0925
7.0898
7.0838
6.7832
6.7534
6.7381
6.7084
6.1813
6.1363
5.7508
5.7210
BRUKER
NAME Aug08xf-PhOPh-GPC
EXPNO 1
PROCNO 1
Date_ 20160808
Time 10.24
INSTRUM spect
PROBHD 5 mm PABBO BB-
PULPROG zg30
TD 32768
SOLVENT CDCl3
NS 8
DS 0
SWH 8223.685 Hz
FIDRES 0.250967 Hz
AQ 1.9923444 sec
RG 170.8
DW 60.800 usec
DE 6.50 usec
TE 298.9 K
D1 1.00000000 sec
TD0 1
======== CHANNEL f1 ========
SFO1 400.1324710 MHz
NUC1 1H
P1 13.40 usec
SI 65536
SF 400.1300097 MHz
WDW EM
SSB 0
LB 0.30 Hz
GB 0
PC 0.50
O
F
F
F
F
6k
9.5 9.0 8.5 8.0 7.5 7.0 6.5 6.0 5.5 5.0 4.5 4.0 3.5 3.0 2.5 2.0 1.5 1.0 ppm
4.10
1.01
4.01
1.00
1.05
1.06

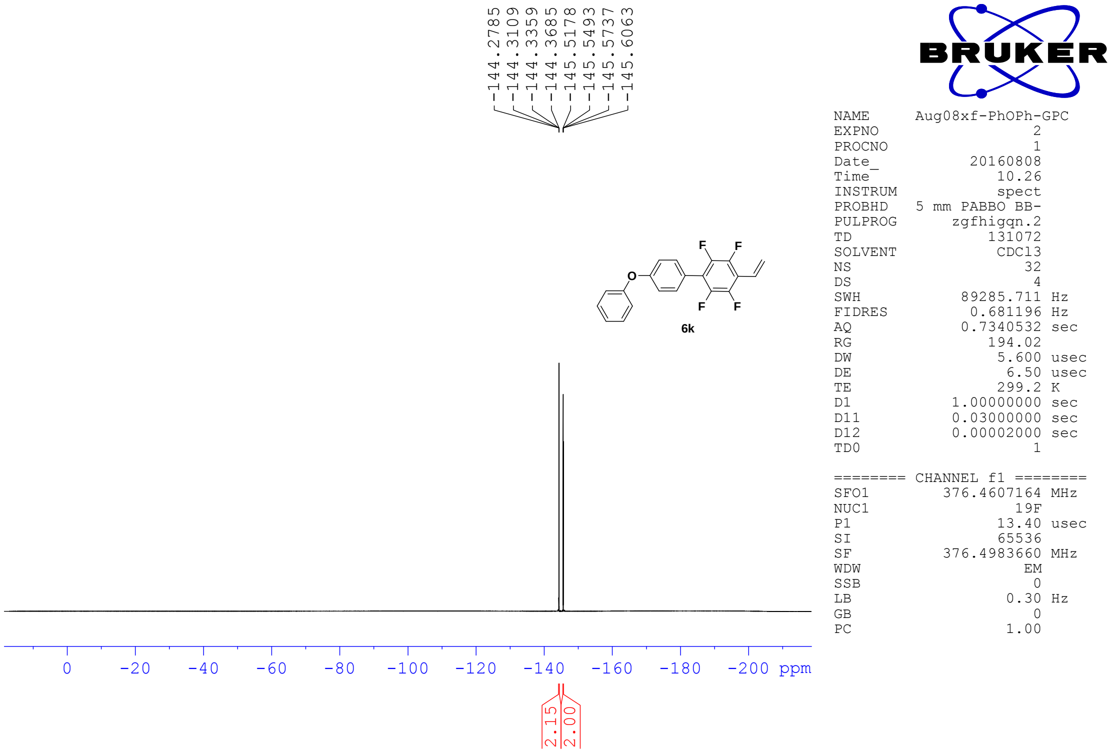

-144.2785
-144.3109
-144.3359
-144.3685
-145.5178
-145.5493
-145.5737
-145.6063
BRUKER
NAME Aug08xf-PhOPh-GPC
EXPNO 2
PROCNO 1
Date_ 20160808
Time 10.26
INSTRUM spect
PROBHD 5 mm PABBO BB-
PULPROG zgfhigqn.2
TD 131072
SOLVENT CDCl3
NS 32
DS 4
SWH 89285.711 Hz
FIDRES 0.681196 Hz
AQ 0.7340532 sec
RG 194.02
DW 5.600 usec
DE 6.50 usec
TE 299.2 K
D1 1.00000000 sec
D11 0.03000000 sec
D12 0.00002000 sec
TD0 1
======== CHANNEL f1 ========
SFO1 376.4607164 MHz
NUC1 19F
P1 13.40 usec
SI 65536
SF 376.4983660 MHz
WDW EM
SSB 0
LB 0.30 Hz
GB 0
PC 1.00
O
F
F
F
F
6k
0 -20 -40 -60 -80 -100 -120 -140 -160 -180 -200 ppm
2.15
2.00

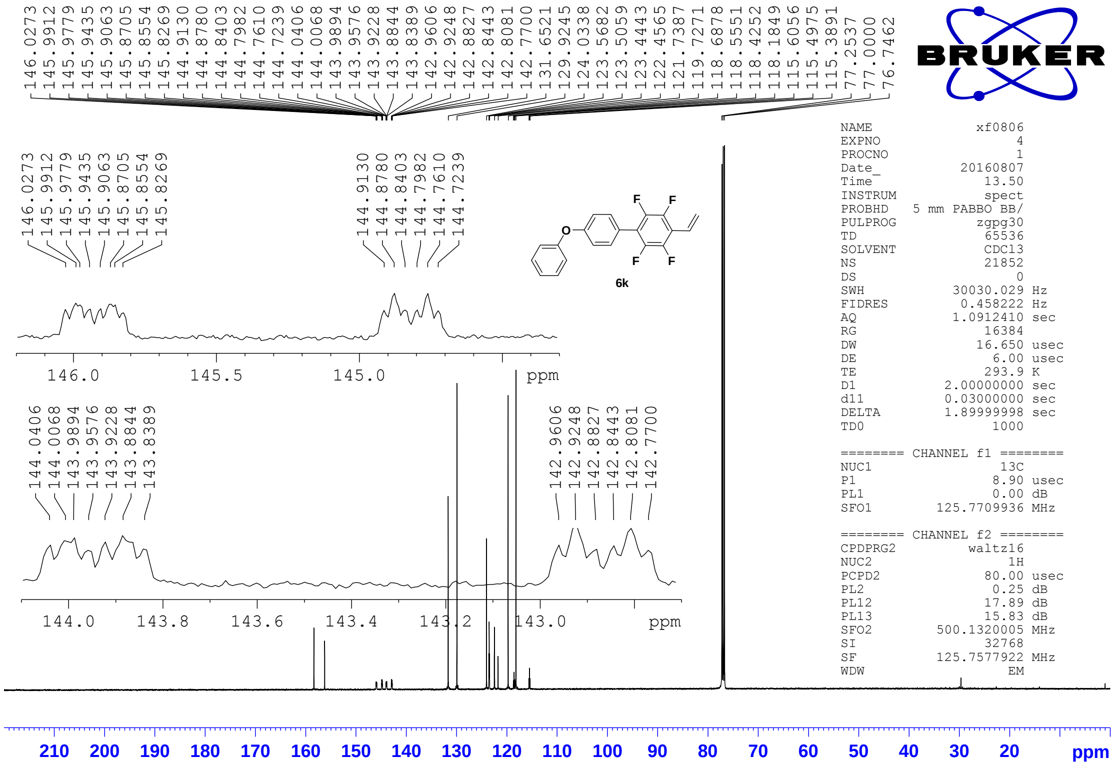
146.0273
145.9912
145.9779
145.9435
145.9063
145.8705
145.8554
145.8269
144.9130
144.8780
144.8403
144.7982
144.7610
144.7239
144.0406
144.0068
143.9894
143.9576
143.9228
143.8844
143.8389
142.9606
142.9248
142.8827
142.8443
142.8081
142.7700
131.6521
129.9245
124.0338
123.5682
123.5059
123.4443
122.4565
121.7387
119.7271
118.6878
118.5551
118.4252
118.1849
115.6056
115.4975
115.3891
77.2537
77.0000
76.7462
BRUKER
F
F
F
F
O
6k
NAME xf0806
EXPNO 4
PROCNO 1
Date_ 20160807
Time 13.50
INSTRUM spect
PROBHD 5 mm PABBO BB/
PULPROG zgpg30
TD 65536
SOLVENT CDCl3
NS 21852
DS 0
SWH 30030.029 Hz
FIDRES 0.458222 Hz
AQ 1.0912410 sec
RG 16384
DW 16.650 usec
DE 6.00 usec
TE 293.9 K
D1 2.00000000 sec
d11 0.03000000 sec
DELTA 1.89999998 sec
TD0 1000
======== CHANNEL f1 ========
NUC1 13C
P1 8.90 usec
PL1 0.00 dB
SFO1 125.7709936 MHz
======== CHANNEL f2 ========
CPDPRG2 waltz16
NUC2 1H
PCPD2 80.00 usec
PL2 0.25 dB
PL12 17.89 dB
PL13 15.83 dB
SFO2 500.1320005 MHz
SI 32768
SF 125.7577922 MHz
WDW EM
146.0 145.5 145.0 ppm
144.0 143.8 143.6 143.4 143.2 143.0 ppm
210 200 190 180 170 160 150 140 130 120 110 100 90 80 70 60 50 40 30 20 ppm
